# Immune surveillance on the insect body surface recognizes a pathogen-derived fungal protease to activate defenses

**DOI:** 10.1038/s41467-026-72836-4

**Published:** 2026-05-09

**Authors:** Jun Li, Qi Xiao, Petros Ligoxygakis, Yuxian Xia

**Affiliations:** 1 Genetic Engineering Research Centre, School of Life Sciences, Chongqing University, Chongqing, China; 2 National Engineering Research Center of Microbial Pesticides (Joint institute-Chongqing University), Chongqing Engineering Research Center for Fungal Insecticides, Chongqing, China; 3 Key Laboratory of Gene Function and Regulation Technology, Chongqing University, Chongqing, China; 4 Institute of Advanced Interdisciplinary Studies, Chongqing University, Chongqing, China; 5 Department of Biochemistry, South Parks Rd, University of Oxford, Oxford, United Kingdom

**Keywords:** Innate immunity, Fungal host response

## Abstract

Immune systems must distinguish between pathogens and commensals to mount effective responses. However, how hosts discriminate among fungi at surface barriers is largely unknown. Here, we report a surveillance mechanism on the locust body surface that couples general fungal recognition with pathogen-specific activation. The host immulectin-1 (IML1) binds surface-exposed fungal mannans, but immune activation requires cleavage by fungal protease SP28 to release bioactive peptide. The protease is found in most fungi, but high evolutionary divergence confers its host-specific activity. Disrupting the IML1-SP28 interaction, either by deleting fungal *SP28*, silencing host *IML1*, or blocking IML1 with excess mannan, abrogates immune responses and accelerates host mortality. This protease-gated checkpoint suggests an evolutionarily conserved principle in insect-fungal interactions, with potential implications for developing novel biopesticides and antifungal agents.

## Abstract (web summary)

Early immune surveillance promotes effective antifungal defense. Here, the authors show that a secreted lectin, IML1, binds diverse fungal spores on the cuticle, but immunity is triggered only upon cleavage by a pathogen specific protease, SP28.

## Introduction

Fungi are ubiquitous in the environment, colonizing the surfaces of plants and animals as both symbionts and pathogens^1–3^. While hosts have evolved complex defenses against invasive fungi^4–6^, they are also perpetually exposed to non-pathogenic spores that pose no threat^7^. Discriminating between these two groups *before* fungal penetration is critical to avoid wasteful immune activation and maintain homeostasis^8^. Such discrimination is exemplified by commensal fungi *Malassezia* spp., which persistently colonize human skin typically without provoking inflammation^9^, and non-entomopathogenic fungi *Fusarium oxysporum* and *Leucoagaricus gongylophorus,* which cannot provoke an acute immune response in leaf beetles and leaf-cutter ants, respectively^10,11^. These observations suggest that hosts tolerate non-invasive fungi by discriminating them from invasive pathogens. Hosts can detect on their surface pathogen-specific danger signals to selectively activate defenses. In plants, rice rapidly responds (within 5-hours) to *Magnaporthe oryzae* spores^12^. In animals, *Candida auris* rapidly induces IL-17A production in murine skin through T cell activation^13^, and *Metarhizium acridum* conidia activate pro-phenoloxidase (a key zymogen in melanization and defense) transcription within 2 hours of attachment to locust cuticles^14^. However, the mechanisms enabling *pre*-*invasion* discrimination, particularly on body surfaces like skin, leaves, or exoskeletons, remain unresolved.

Current paradigms focus on intracellular or systemic responses *post*-invasion, such as β-glucan recognition by Toll-like receptors/Dectin-1 in mammals^15^, GNBP3 in insects^16^, or LysM receptor in plants^17^. However, the shared surface molecules (e.g., mannans, β-glucans) between pathogenic and non-pathogenic fungi^18^ obscure surface-level discrimination mechanisms.

Here, we identify IML1 on the body surface of the locust by conidial affinity purification, mass spectrometry, and recombinant expression. IML1 is a lectin receptor that integrates microbe-associated molecular pattern (MAMP) recognition with pathogen-derived protease activity to trigger an immune response. Unlike canonical pattern recognition, IML1 requires proteolytic cleavage by fungal protease SP28 with host-specific activity to initiate defense. This mechanism likely preserves host immune resources for genuine threats. This work reveals a conserved strategy for immune discrimination at the host surface, suggesting potential relevance for managing fungal infections in the clinic, agriculture, and microbiome engineering.

## Results

### Locust innate immunity discriminates between pathogenic and non-pathogenic fungi

The entomopathogenic fungi *M. acridum* and *Metarhizium anisopliae* readily adhere to the locust cuticle, initiating invasion^19^. To confirm the development of non-entomopathogens on the host surface, the conidia from non-entomopathogenic fungi, including *Candida albicans*, *Aspergillus flavus*, and *Alternaria alternata*, were inoculated and allowed to attach to the cuticle. In contrast to entomopathogenic fungal spores^19^, these non-entomopathogenic spores failed to germinate on the locust cuticle and were incapable of host invasion (Supplementary Fig. 1). Since both entomopathogenic and non-entomopathogenic fungi co-exist on the body surface, we compared the immune responses of locusts inoculated with spores of entomopathogenic fungi (*M. acridum*, and *M. anisopliae*) and non-entomopathogenic fungi (*C. albicans*, *A. flavus*, and *A. alternata*) to determine whether the host innate immunity can discriminate between these two groups of fungi prior to conidial germination.

**Fig. 1:**
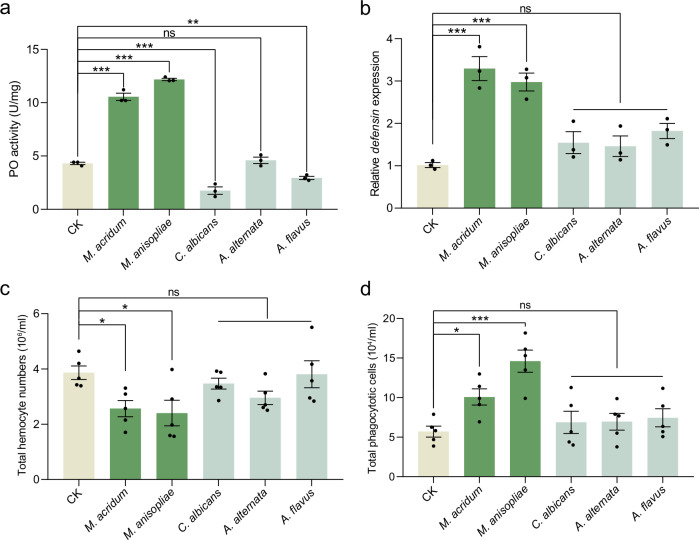
Spores from entomopathogenic fungi have the specific ability to activate locust’s immunity. Immune functions were compared in locusts non-inoculated (control check; CK), or inoculated with spores (topical application of 5 μL at 1 × 10^8^ conidia/mL to abdominal segments) from entomopathogenic (*M. acridum* and *M. anisopliae*) or non-entomopathogenic (*C. albicans*, *A. flavus*, and *A. alternata*) fungi. For all immune assays (**a**–**d**), cuticle and epidermal cell samples were dissected from the second and third abdominal segments at 4 hours post-inoculation. **a** Phenoloxidase (PO) activity in cuticle (*p* < 0.0001 for *M. acridum*; *p* < 0.0001 for *M. anisopliae*). **b** Transcription of *defensin* in epidermal cells (*p* < 0.0001 for *M. acridum*; *p* = 0.0002 for *M. anisopliae*). **c** Total circulating hemocyte number (*p* = 0.0494 for *M. acridum*; *p* = 0.0236 for *M. anisopliae*). **d** Phagocytic cell numbers (*p* = 0.0496 for *M. acridum*; *p* < 0.0001 for *M. anisopliae*). The results were obtained from at least three replicates. Asterisks indicate significant differences analyzed by one-way ANOVA with Tukey’s HSD post-hoc test (*p < 0.05; **p < 0.01; ns, not significant). Data represent the mean ± standard error of the mean (SEM) of three (**a**, **b**) or five (**c**, **d**) biologically independent samples. Source data are provided as a Source Data file.

Unlike *Metarhizium* spp. (*M. acridum*: 2.49-fold, *p* < 0.0001; *M. anisopliae*: 2.88-fold, *p* < 0.0001), non-entomopathogens cannot trigger significant phenoloxidase (PO) activity in the cuticle (Fig. 1a). The *defensin* mRNA in epidermal cells remained unchanged after exposure to all the three non-entomopathogens, contrasting sharply with entomopathogen-induced upregulation (*M. acridum*: 3.27-fold, *p* < 0.0001; *M. anisopliae*: 2.95-fold, *p* = 0.0002; Fig. 1b). In addition, entomopathogenic fungi reduced the number of circulating hemocytes (*M. acridum*: 0.66-fold, *p* = 0.0494; *M. anisopliae*: 0.62-fold, *p* = 0.0236; Fig. 1c), while increasing the number of phagocytes (*M. acridum*: 1.77-fold, *p* = 0.0496; *M. anisopliae*: 2.56-fold, *p* < 0.0001; Fig. 1d). However, the non-entomopathogenic fungi could not trigger cellular responses (Fig. 1c, d), confirming the absence of systemic immune mobilization. These findings suggest that the locust’s immune system can discriminate between entomopathogenic and non-entomopathogenic spores upon cuticle attachment, selectively activating innate immune responses in the cuticle, epidermal cells, and hemocytes when faced with an entomopathogenic threat. Moreover, selective immune activation against entomopathogens aligns with the observation that only their spores can germinate and successfully invade the cuticle.

### Locust IML1 surveils spores via fungal mannan

Since innate immunity responds to entomopathogenic conidia on the body surface before germination, we hypothesized that local receptors on the host surface could recognize ungerminated fungal spores. To identify potential conidia-binding receptors, *M. acridum* spores were inactivated and fixed for a pull-down experiment using total protein extracted from locust wings (including epicuticle and hemolymph), the epicuticle (excluding hemolymph), and cell-free hemolymph (excluding epicuticle and hemocytes) (Supplementary Fig. 2a). Eluted protein samples through conidial affinity chromatography were identified by Liquid Chromatography with Tandem Mass Spectrometry (LC-MS/MS) analysis. Proteins matching fewer than two unique peptide fragments were excluded from the analysis. In total, 134 proteins from the cell-free hemolymph, 423 proteins from the integument, and 516 proteins from the hindwing were identified. Thirty-eight proteins were expressed in all three tissues (Supplementary Data 1 and Supplementary Fig. 2b). Further analysis of signal peptides and transmembrane domains revealed that 17 of these shared proteins were predicted to be secreted. Among them, there were four potential recognition receptors, including a lipoprotein, obstructor E2, GNBP3, and a C-type lectin, that were capable of binding to the conidial surface (Supplementary Table 1). The lipoprotein has the potential to bind hydrophobin-3 of *M. acridum*, a feature found specifically in specialists^14^. The obstructor E2 can bind chitin, a component of the inner cell wall^18^ GNBP3 is undetectable on the cuticle in silkworms^20,21^. Phylogenetic analysis revealed that the closest homologous protein to this lectin in insects is immulectin-1 (IML1). IML1 contains two carbohydrate-recognition domains (CRDs), N-terminal signal peptide, and lacks a transmembrane domain (Supplementary Fig. 2c). Structural analysis revealed that IML1 is not uniformly hydrophilic but possesses clustered hydrophobic patches concentrated on one face of its N-terminal region and within the central core of both CRDs, with the opposite face being predominantly hydrophilic (Supplementary Fig. 2d). This amphipathic architecture, typical of proteins functioning at a lipid-air interface, likely enables IML1 to associate with cuticular lipids via hydrophobic patches while exposing hydrophilic surfaces for mannan recognition, providing a biophysical basis for its localization and function on the epicuticle. Therefore, we selected IML1 as a candidate receptor protein for further investigation.

**Fig. 2:**
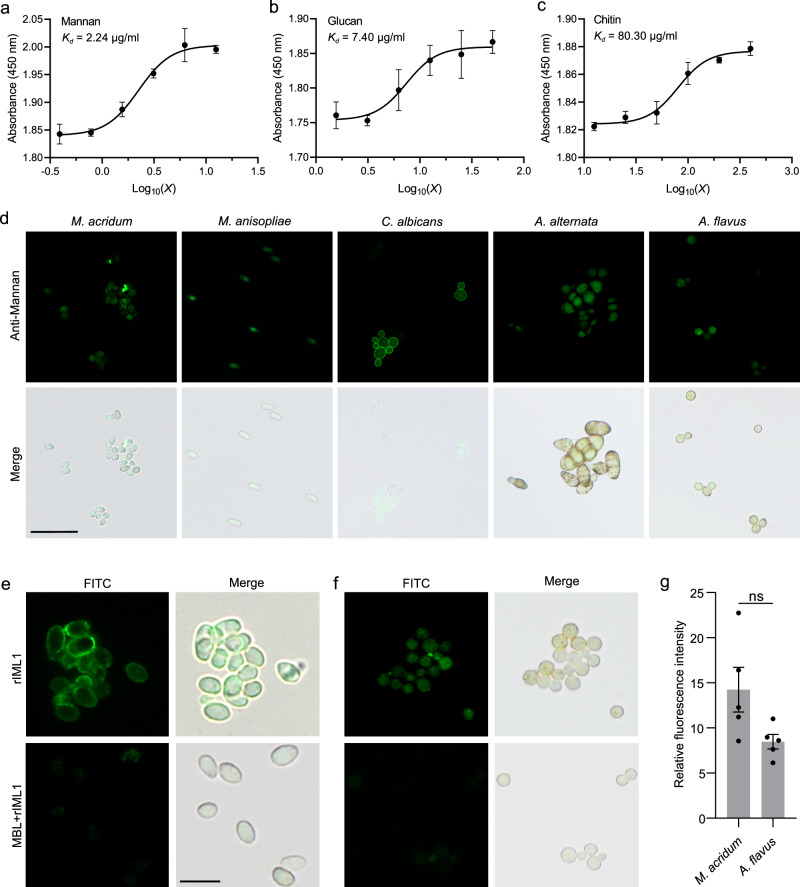
IML1 specifically recognizes and binds to mannan on the fungal surface. **a**–**c** In vitro binding assays demonstrate that recombinant IML1 (rIML1) exhibits the strongest affinity for mannan, followed by glucan and chitin. *K*_*d*_ represents the dissociation constant. The x-axis, Log10(*X*), indicates the log10-transformed glycan concentration (*X*, μg/mL) (**d**–**f**). This specific interaction was confirmed on intact conidia. **d** Staining reveals that mannan is abundant on the spore surface of all fungi. Scale bar, 10 μm. Correspondingly, FLAG-tagged rIML1 robustly binds to *M. acridum* (**e**) and *A. flavus* (**f**). To verify binding specificity, spores were pre-incubated with a mannan-binding lectin (MBL) to mask surface mannans prior to rIML1 addition, which successfully abrogated rIML1 binding (MBL control). Scale bar, 5 μm. **g** Quantification of rIML1 binding intensity from (**e**) and (**f**) by ImageJ analysis. Data are shown as mean ± SEM of three (**a**–**c**) or five (**g**) biological replicates. Micrographs in (**d**–**f**) are representative of three independent experiments with similar results. Significant differences in (**g**) were determined using a two-sided Student’s *t* test. ns, not significant (*p* = 0.0571). Source data are provided as a Source Data file.

Pathogenic and non-pathogenic fungi share similar cell wall structures^18^ such as mannan, glucan, and chitin decorate their conidial walls^22,23^. To evaluate the potential binding affinity of IML1 to these three glycans, molecular docking analyses were conducted. IML1 displayed distinct docking affinities for these polysaccharides, with values in ascending order: − 12.53 kcal/mol for mannan, − 10.80 kcal/mol for glucan, and − 9.39 kcal/mol for chitin (Supplementary Fig. 2e). The binding affinity (dissociation binding constant, *K*_*d*_) was experimentally measured using purified recombinant IML1 (rIML1). rIML1 had the highest affinity for mannan (*K*_*d*_ = 2.24 μg/mL), followed by glucan (*K*_*d*_ = 7.40 μg/mL), and chitin (*K*_*d*_ = 80.30 μg/mL) (Fig. 2a–c). Immunofluorescence microscopy showed the presence of mannans on the conidial surface of both entomopathogenic and non-entomopathogenic fungi (Fig. 2d). Based on this observation, we hypothesized that IML1 detects conidia by binding to surface mannan. To test this, we compared the binding of recombinant IML1 (rIML1) to *M. acridum* (entomopathogen) and *A. flavus* (non-entomopathogen) conidia, either precoated with a mannan-binding lectin (MBL) or left untreated. rIML1 bound strongly to the conidial surface of both fungi, and pretreatment with the mannan antibody significantly reduced this binding (Fig. 2e, f). Quantitative analysis revealed no significant difference in IML1 binding intensity between *M. acridum* and *A. flavus* (Fig. 2g). These results confirm that IML1 recognizes pathogenic and non-pathogenic fungal spores through their surface mannans.

### IML1 localizes to the cuticle surface to mediate antifungal defense

**Fig. 3:**
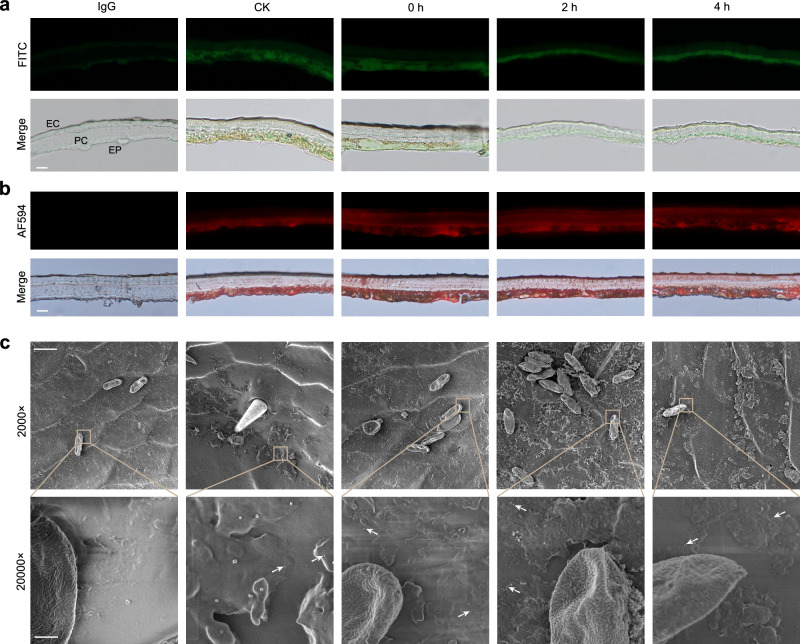
In situ analysis of *IML1* mRNA and protein expression showing IML1 secretion from epidermal cells to the body surface, and clustering of IML1 protein around attached *M. acridum* conidia. **a** *IML1* mRNA in the epidermal cells of a locust abdomen inoculated with *M. acridum* conidia four hours earlier. Scale bar, 20 μm. **b** Immunofluorescence analysis showing IML1 protein distribution in the second and third abdominal segments at 0-, 2-, and 4-hours post-inoculation with *M. anisopliae* conidia. Red fluorescence indicates IML1 protein detected by anti-IML1 polyclonal antibody and Alexa Fluor 594 (AF594)-conjugated secondary antibody. For the negative control, normal IgG was used as the primary antibody. Scale bar 20 μm. **c** Distribution of IML1 protein on body surface after inoculation of locust with *M. anisopliae* visualized by scanning electron microscopy with immunogold labeling (representative images at two magnifications are shown). White arrows indicate 35 nm colloidal gold particles conjugated to a secondary antibody, which is bound to the anti-IML1 primary antibody. A parallel control using normal IgG as the primary antibody. Scale bars: 10 μm (upper panel, 2000 × magnification); 1 μm (lower panel, 20,000 × magnification). EC, epicuticle; PC, procuticle; EP, epidermal cells. Micrographs in (**a**–**c**) are representative of three independent experiments with similar results.

To test whether the involvement of host surface IML1 and conidial mannan is critical in the host’s discrimination between pathogenic and non-pathogenic spores, we first analyzed the basal and induced expression pattern of IML1. In unchallenged locusts, IML1 transcript was detectable at low levels across tissues, with relatively higher basal expression in hemocytes (Supplementary Fig. 3a). Upon topical inoculation with pathogenic *M. acridum* conidia, a rapid and potent induction of *IML1* transcription occurred within 4 hours, most pronounced in hemocytes (8.01-fold increase, *p* = 0.0001), followed by the fat body and epidermal cells (Supplementary Fig. 3a). Extending the time course revealed a biphasic expression pattern: transcript levels declined sharply by 6 hours post-inoculation, coinciding with the onset of spore germination on the cuticle^24^, followed by a second, more gradual increase from 8 to 16 h (Supplementary Fig. 3a). In contrast, non-pathogenic *A. flavus* spores failed to elicit any significant *IML1* upregulation at any time point examined (Supplementary Fig. 4a). This biphasic induction suggests that IML1 is mobilized in two waves: an early phase for surface surveillance and a later phase potentially supporting systemic defense during active infection. Fluorescent in situ hybridization (FISH) localized *IML1* mRNA to the integument, revealing a time-dependent increase in epidermal *IML1* expression at 2- and 4-hours post-inoculation with *M. acridum* spores. Scramble control probes showed no signal, confirming the specificity of IML1 mRNA detection. While basal IML1 expression was detectable in non-inoculated locusts (CK), pathogen challenge substantially enhanced transcription in epidermal cells (Fig. 3a). These results demonstrate that pathogenic conidia activate *IML1* transcription upon cuticle attachment, whereas non-pathogenic spores fail to trigger this response.

To investigate the distribution of IML1, we generated an IML1-specific antibody (Supplementary Fig. 3b, c). Using this antibody, we found that after conidial inoculation of *M. acridum*, IML1 levels increased substantially in the hemocytes and cell-free hemolymph. In the integument, we observed a moderate increase in IML1 abundance, whereas expression remained low in the fat body (Supplementary Fig. 3d). To determine whether IML1 was secreted and accumulated around conidia on the body surface, we visualized IML1 protein in cuticle sections and on the body surface using immunofluorescence microscopy (IF) and scanning electron microscopy before and at different intervals after conidial attachment. IF analysis revealed the distribution of IML1 in epidermal cells, the procuticle and the epicuticle (Fig. 3b). IF analysis revealed that *A. flavus* did not cause IML1  to accumulate near the epicuticle (Supplementary Fig. 4b). Scanning electron microscopy (SEM) revealed the presence of pre-existing IML1 protein on the surface of the locust’s body prior to conidial attachment, which could then readily accumulate around the attached conidia of pathogenic and non-pathogenic fungi (Fig. 3c and Supplementary Fig. 4c). Further, to directly track IML1 trafficking from in vivo to fungal attachment sites, we employed a SunTag system^25^. IML1 fused with 12 × GCN4 peptide array was injected into the hemocoel and detected on the cuticle surface using ScFv-GFP. Following topical inoculation with *M. acridum* conidia, IML1-12 × GCN4 progressively accumulated around attached spores at 2 and 4 h, providing direct evidence for IML1 secretion from the hemolymph to the body surface (Supplementary Fig. 5). Together, the SunTag tracing, immunofluorescence, and immunogold-SEM data demonstrate that IML1 is secreted and dynamically accumulates on the cuticle surface, specifically at sites of fungal spore attachment.

To investigate whether IML1 could mediate the locust’s innate immune response in the integument and hemocytes upon recognition of conidial mannan, we inoculated the locust’s body surface with normal conidia, conidia treated with a mannan-binding lectin (MBL) or treated the body surface with an anti-IML1 antibody prior to inoculation. Then, we measured cuticular PO activity, *defensin* transcription in epidermal cells, hemocyte migration, and phagocytosis. Compared with normal conidia treatment, PO activity and *defensin* expression were significantly decreased in locusts treated with MBL or the anti-IML1 antibody (Supplementary Fig. 6a, b). The circulating hemocytes can rapidly respond to pathogenic infections by enhancing phagocytosis and aggregating at the basal membrane^14,26,27^. We measured the total hemocyte numbers and phagocytic cell numbers in the hemolymph following the aforementioned treatments. Topical inoculation of normal conidia reduced the number of circulating hemocytes in the hemolymph and increased the phagocytic cell numbers, while treatment with MBL or the anti-IML1 antibody suppressed the hemocyte response (Supplementary Fig. 6c, d).

To evaluate the function of IML1 during fungal infection, we knocked down *IML1* expression by injecting dsRNA targeting *IML1* (ds*IML1*) along with an IML1-specific antibody (ds*IML1*/Ab). In addition, we simulated IML1 overexpression by injecting rIML1 into the hemocoel. The treated locusts were then topically inoculated with *M. acridum* conidia. Compared to the control group (ds*GFP*) (lethal median time, LT_50_ = 5.83 days), ds*IML1*/Ab significantly decreased the survival rate of locusts (LT_50_ = 5.06 days, *p* = 0.019), whereas rIML1 showed a trend toward increased resistance to fungal infection (LT_50_ = 6.41 days, *p* = 0.062) (Supplementary Fig. 6e, f). The lethal median dose of spores (LD_50_) was 14.54-fold lower in the ds*IML1*/Ab treatment compared to the ds*GFP* control treatment (*p* = 0.0002) (Supplementary Fig. 6g). In contrast, rIML1 treatment increased the LD_50_ by 1.73-fold (*p* < 0.0001). Thus, IML1 is a crucial determinant of locust resistance to fungal infections.

Taken together, these data establish that IML1-mannan binding on the host body surface is necessary for antifungal defense upon attachment. However, since IML1 binds mannans on both pathogenic and non-pathogenic fungi, this interaction alone cannot explain the pathogen-specific immune activation observed in Fig. 1. This paradox suggests the existence of an additional, pathogen-encoded signal that converts IML1 binding into immune activation, leading us to investigate the role of fungal proteases.

### Fungal SP28 cleaves IML1 to mobilize immune defense

**Fig. 4:**
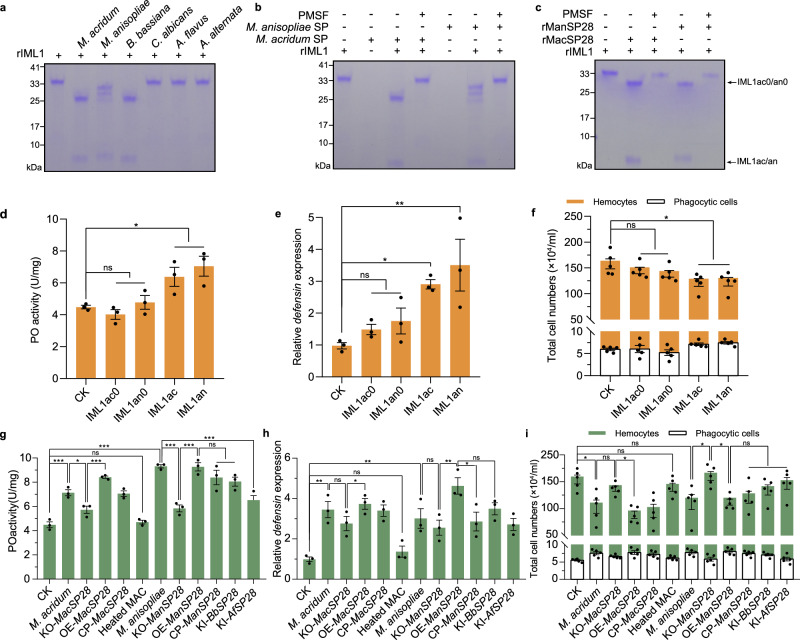
Fungal protease SP28 cleaves IML1 to trigger humoral and cellular immunity in locusts. **a**–**c** Biochemical analysis of IML1 cleavage by fungal proteases. **a** Representative Coomassie blue-stained SDS-PAGE gel showing the proteolytic activity of protein extracts from the conidia of six different fungi on recombinant IML1 (rIML1). **b** Proteolytic activity of protein extracts from *M. anisopliae* or *M. acridum* conidia on rIML1, and its inhibition by the serine protease inhibitor phenylmethylsulfonyl fluoride (PMSF). **c** Cleavage of rIML1 by recombinant serine proteases ManSP28 (rManSp28, KAK8918209.1) and MacSP28 (rMacSP28, EFY85443.1). The IML1ac0/an0 represent large fragments produced by proteases from *M. acridum* and *M. anisopliae*, respectively, while IML1ac/an represent the corresponding small peptides. **d**–**f** Immune responses in locusts at 4 hours post-inoculation with 1 μg of purified IML1-derived peptides per insect. **d** Phenoloxidase (PO) activity in cuticle (*p* = 0.0317 for IML1ac; *p* = 0.0093 for IML1an). **e** Defensin transcription in epidermal cells (*p* = 0.0280 for IML1ac; *p* = 0.0056 for IML1an). **f** Total hemocyte and phagocytic cell numbers in hemolymph (*p* = 0.0131 for IML1ac; *p* = 0.0166 for IML1an). Small peptides IML1ac and IML1an, but not large fragments IML1ac0 and IML1an0, activate immune responses. **g**–**i** Immune responses in locusts at 4 h post-inoculation. For *M. acridum*: wild type (WT), heat-inactivated WT (Heated MAC), KO-*MacSP28*, OE-*MacSP28*, and complementation (CP). For *M. anisopliae*: WT, KO-*ManSP28*, OE-*ManSP28*, and strains in which the *ManSP28* locus was replaced by *BbSP28* (KI-*BbSP28*) or *AfSP28* (KI-*AfSP28*). **g** Phenoloxidase (PO) activity in the cuticle. **h** Transcription of *defensin* in epidermal cells. **i** Total circulating hemocyte and phagocytic cell numbers in the hemolymph. Data are presented as mean ± SEM of three (**d**, **e**, **g**, **h**) or five (**f**, **i**) biologically independent samples. Significant differences were analyzed by one-way ANOVA with Tukey’s HSD post-hoc test (*p < 0.05; **p < 0.01; ***p < 0.001; ns, not significant). All experiments were repeated at least three times and yielded similar results. Source data are provided as a Source Data file.

While IML1 mediates humoral defenses in the cuticle and recruits hemocytes to fungal attachment sites, its broad affinity for both entomopathogenic and non-entomopathogenic mannans cannot explain the pathogen-specific immune activation observed in Fig. 1. This paradox points to a critical missing component: a pathogen-encoded licensing signal that converts IML1 binding into immune activation. Given that many proteases have been reported on the surface of fungal spores^28^, we hypothesized that the missing licensing signal corresponds to proteolytic activity uniquely deployed by entomopathogens. To test this hypothesis, conidial surface proteins were extracted from three entomopathogenic fungi, *M. acridum*, *M. anisopliae*, and *Beauveria bassiana,* as well as from three non-entomopathogenic fungi, *C. albicans*, *A. flavus*, and *A. alternata*. All these extracts exhibited proteolytic activity on bovine serum albumin (BSA) (Supplementary Fig. 7a). Incubation of IML1 with conidial protein extracts from *M. acridum* and *B. bassiana* resulted in its cleavage into two major fragments, while extract from *M. anisopliae* generated four fragments. In contrast, none of the extracts from the non-entomopathogenic fungi *C. albicans*, *A. flavus*, and *A. alternata* were able to cleave IML1 (Fig. 4a). IML1 cleavage by conidial protein extracts from the two *Metarhizium* spp. was inhibited by phenylmethylsulfonyl fluoride (PMSF), suggesting the involvement of serine proteases (Fig. 4b).

To identify the precise cleavage products, the IML1 fragments generated by incubation with *M. acridum* or *M. anisopliae* conidial surface extracts were excised from gels and subjected to LC-MS/MS analysis. This analysis unambiguously identified two major peptide sequences corresponding to the cleavage fragments. The released small bioactive peptide from *M. acridum* cleavage was designated IML1ac, and that from *M. anisopliae* was designated IML1an (Supplementary table 2). Mass spectrometry mapping confirmed that both peptides originate from the N-terminal region of IML1 and share a core sequence, with IML1an containing an additional C-terminal extension (Supplementary Fig. 7b, c). Bioinformatic analysis of the cleavage junctions revealed a conserved preference for a proline-X (where X is Arg or a hydrophobic residue) motif, characteristic of certain serine protease substrates. Furthermore, LC-MS/MS analysis of protein extracts identified two serine proteases in *M. anisopliae* and ten in *M. acridum* (Supplementary Table 3). For comparison, genome analysis identified two serine proteases with signal peptides in *C. albicans* and *A. flavus*, and nine in *A. alternata* (Supplementary table 3). Phylogenetic analysis of the serine proteases from the two *Metarhizium* spp. and the three non-entomopathogenic fungi revealed that the serine protease from *M. anisopliae* (ManSP28) and *M. acridum* (MacSP28) clustered in the same clade, distinct from the serine proteases of non-entomopathogenic fungi (Supplementary Fig. 7d). Therefore, we hypothesized that ManSP28 and MacSP28 are host-specific and responsible for IML1 cleavage. To test this hypothesis, recombinant ManSP28 and MacSP28 were expressed in *E. coli* and used in an in vitro IML1 cleavage assay. Both ManSP28 and MacSP28 proteases cleaved recombinant IML1 into two fragments (Fig. 4c). We assessed the immune effects of the cleavage products. The released small peptides, designated IML1an (from *M. anisopliae* ManSP28) and IML1ac (from *M. acridum* MacSP28), activated innate immunity, whereas the corresponding large residual fragments (IML1an0 and IML1ac0) were inactive (Fig. 4d–f). Bioinformatic analysis revealed that all SP28 homologs possess N-terminal signal peptides, suggesting potential secretion. To verify expression and surface localization, we focused on *A. flavus* as a representative model, given its distinct failure to induce IML1-mediated immunity despite surface binding. Immunofluorescence staining directly confirmed the presence of the native SP28 protein on the conidial surface (Supplementary Fig. 8a). These data demonstrate that the inability of *A. flavus* surface extracts to cleave IML1 stems from intrinsic divergence in enzymatic specificity rather than defective expression or trafficking.

To determine the role of SP28 in entomopathogens during infection, *M. anisopliae* and *M. acridum* strains with *SP28* knockout (KO) or complementation (CP) mutations were constructed. Transcript analysis showed that *SP28* mRNA is detectable in wild-type *M. anisopliae* and *M. acridum* conidia harvested at both 3 and 6 days post-culture (Supplementary Fig. 8b). Staining with an anti-FLAG antibody confirmed that the overexpressed homologous proteases were correctly secreted to the conidial surface (Supplementary Fig. 8c d). We first assessed fungal fitness using the *M. anisopliae* mutants. When grown on nutrient-rich artificial medium (1/4 SDAY), KO-*ManSP28* showed normal colony development comparable to wild-type. However, when *M. anisopliae* was cultured on locust hindwings, deletion of *SP28* significantly impaired fungal growth capacity, resulting in  a 15.8% reduction in colony diameter after cuticle penetration (*p* = 0.035; Supplementary Fig. 9). These findings indicate that SP28 facilitates fungal colonization and invasion of the cuticle, explaining its evolutionary retention despite triggering host immunity.

Next, to further investigate the role of SP28 in immune activation, we generated overexpression (OE) strains to serve as positive controls. The body surface of locusts was inoculated with conidia from wild-type, KO, OE, and CP strains to study host immune responses. Locusts inoculated with KO-*MacSP28* or KO-*ManSP28* conidia displayed significantly reduced PO activity in the cuticle, *defensin* expression in epidermal cells, and total phagocytic cell numbers compared to locusts inoculated with WT, CP, or OE counterparts, with the most pronounced reduction observed relative to the OE strains (Fig. 4g–i). The compromised host defense was accompanied by enhanced invasiveness of the KO-*MacSP28* and KO-*ManSP28* mutants, resulting in significantly higher fungal DNA loads in the hemolymph compared to their respective WT, CP, or OE counterparts, again with the greatest increase relative to the OE (Supplementary Fig. 10). To rigorously confirm that proteolytic activity, rather than simple receptor occupancy, was the prerequisite for signaling, we utilized heat-inactivated conidia. Binding assays revealed that IML1 still bound to heat-inactivated conidia from both *M. acridum* and *M. anisopliae*, including the wild type (WT) and KO-*SP28* (Supplementary Fig. 11a, b). However, heat-inactivated *M. acridum* spores failed to trigger host innate immunity (Fig. 4g–i). This functional uncoupling of binding from activation definitively proves that IML1 acts as a safety lock requiring specific proteolytic cleavage by SP28 to release the immune‑activating signal.

To further delineate functional specificity, we generated a *M. anisopliae* strain in which the native *ManSP28* gene locus was replaced by orthologs from *B. bassiana* (*BbSP28*) or *A. flavus* (*AfSP28*), termed KI-*BbSP28* and KI-*AfSP28*, respectively. Immunofluorescence confirmed that both *AfSP28* and *BbSP28* were correctly targeted to the conidial surface of *M. anisopliae* (Supplementary Fig. 11c). Despite this successful localization, the strain expressing AfSP28 failed to elicit host immune responses. In stark contrast, expression of *BbSP28* fully restored immune responses to wild-type levels (Fig. 4g–i). These results establish that the capacity to mobilize host defense is a specialized functional trait strictly confined to SP28 variants from entomopathogens. Finally, to determine whether IML1an, resulting from the cleavage of IML1 by fungal SP28, could penetrate the insect cuticle and bind underlying epidermal cells, recombinant IML1an carrying a histidine tag was expressed in *E. coli* and purified. Immunofluorescence analysis of tissue sections showed that IML1an could penetrate the cuticle to reach epidermal cells (Supplementary Fig. 12).

In summary, our collective data from biochemical characterization and functional genetic validation identify a protease-gated immune checkpoint on the insect cuticle. This system operates via a two-step verification mechanism, where the host lectin IML1 acts as a general sensor for fungal mannans, but immune activation is licensed only upon cleavage by the pathogen-secreted protease SP28.

### The IML1-SP28 axis is evolutionarily conserved across diverse insect and fungal taxa

To assess the broader potential of this system, we performed a phylogenetic analysis. This revealed that IML1-like C-type lectins (the locks) are highly conserved and widespread across diverse insect orders, including Hymenoptera, Coleoptera, and Lepidoptera (Supplementary Fig. 13a). This conservation extends beyond insects, as similar mannan-binding lectins were also identified in mammals (surfactant protein D of *Homo sapiens*)^29^ and plants (L-type lectin of *Arabidopsis thaliana*)^30^. This suggests a conserved evolutionary origin for this type of surveillance molecule in barrier immunity. Phylogenetic analysis revealed that animal IML-like lectin are highly divergent from their plant counterparts, whereas insect IML1-like lectins clustered with the plant mannan-binding protein orthologues (Supplementary Fig. 13a). The conservation of this lectin family suggests a similar host-directed strategy could exist beyond insects, which is an area for future research.

Phylogenetic analysis of SP28 resolved seven subclades, distinguishing various pathogenic fungi and non-pathogenic fungi, with a further conserved clade exclusive to entomopathogens and phytopathogens (Supplementary Fig. 13b). The SP28 proteases of non-entomopathogenic fungi *A. alternata* (clade E) and *A. flavus* (clade B) were highly divergent from those of entomopathogens (clade G). Within this major pathogenic clade, we observed a complex evolutionary history. This core group (Clade G) comprised highly specialized insect pathogens such as *Metarhizium*, *Beauveria*, and *Cordyceps*, interspersed with several phytopathogens like Claviceps purpurea. This topology is consistent with the hypothesis that insect pathogenicity within *Hypocreales* repeatedly evolved from plant-associated ancestors via host shifts^31^. Our findings in insects, combined with the presence of homologous lectins in mammals and plants, lead us to hypothesize that an analogous fungal protease-gated recognition mechanism may operate across kingdoms.

### IML1-SP28 interaction governs virulence across insects

**Fig. 5:**
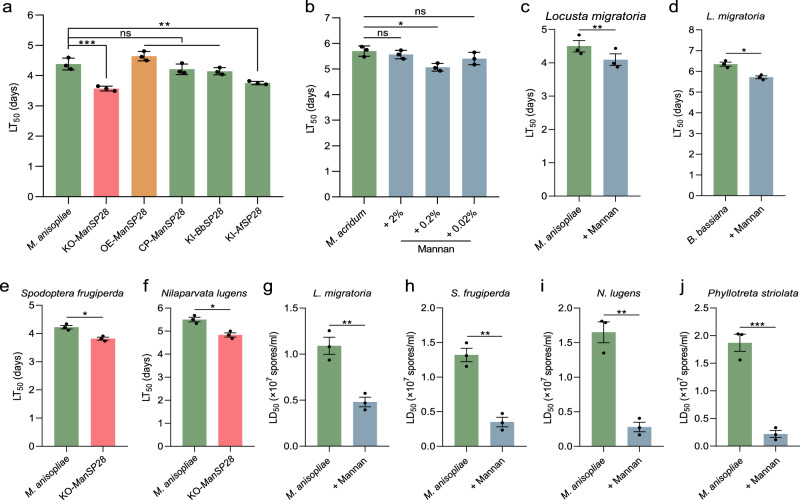
The SP28-mediated protease-gated mechanism is a conserved virulence strategy in entomopathogenic fungi. **a** LT_50_ comparison of locust *L. migratoria* challenged with *M. anisopliae* strains: knockout mutant (KO-*ManSP28*), overexpression strain (OE-*ManSP28*), complemented strain (CP), and in which the *SP28* locus was replaced by *SP28*of *B. bassiana* (KI-*BbSP28*) or *A. flavus* (KI-*AfSP28*) (*p* = 0.0001 for KO-*ManSP28*; *p* = 0.0014 for KI-*AfSP28*). **b** Dose-response analysis of locusts challenged with *M. acridum* conidia co-applied with different mannan concentrations (*p* = 0.0382 for 0.2%). **c**, **d** LT_50_ comparisons of different host-fungus combinations with or without 0.2% mannan co-application: locusts challenged with *M. anisopliae* (**c**, *p* = 0.0082) and *B. bassiana* (**d**, *p* = 0.0105). **e**, **f** LT_50_ comparisons of fall armyworm *Spodoptera frugiperda* (**e**, *p* = 0.0435) and rice planthopper *Nilaparvata lugens* (**f**, *p* = 0.0169) challenged with wild-type *M. anisopliae* or KO-*ManSP28* at 1 × 10^7^ conidia/ml. **g**–**j** LD_50_ comparison of *S. frugiperda* (**g**, *p* = 0.0088), *N. lugens* (**h**, *p* = 0.0032), flea beetles *Phyllotreta striolata* (**i**, *p* = 0.0018), and locusts (**j**, *p* = 0.0005) challenged with *M. anisopliae* at five concentrations with or without 0.2% mannan. For all assays, 30 insects per treatment group were monitored at 12 h intervals. LT_50_ values analyzed by the Kaplan-Meier method and compared using the log-rank test (**a**–**f**). LD_50_ values were calculated by Probit regression. Statistical differences in LD_50_ values (**g**–**j**) were determined using a two-sided Student’s *t* test on log-transformed values. Data are mean ± SEM from three independent experiments. *p < 0.05, **p < 0.01, ***p < 0.001; ns, not significant. All experiments were repeated at least three times with similar results. Source data are provided as a Source Data file.

First, we sought to genetically validate this principle in our primary model. Virulence bioassays against *Locusta migratoria* revealed that deleting SP28 in *M. anisopliae* (KO-*ManSP28*) had a profound effect, accelerating mortality and reducing the median lethal time (LT_50_) by 0.81 days (*p* = 0.0001; Fig. 5a). Conversely, overexpressing *ManSP28* (OE-*ManSP28*) slightly attenuated virulence compared to WT (increasing the LT_50_ by 0.26 days, *p* = 0.2703). To determine if this virulence modulation is governed by the specific activity of entomopathogenic SP28, we assessed the heterologous complementation strains. Expression of the *B. bassiana* ortholog, KI*−BbSP28*, fully restored virulence to WT levels. In contrast, the *A. flavus* ortholog, KI-*AfSP28*, failed to complement the deletion defect, resulting in a hypervirulent phenotype similar to the mutant (LT_50_ reduced by 0.63 days; *p* = 0.0014; Fig. 5a). These results demonstrate that SP28-mediated immune activation requires host-specific protease activity.

Having established SP28’s role genetically, we next developed a non-transgenic strategy to disrupt the IML1-SP28 checkpoint using competitive inhibition with excess mannan. We first confirmed that mannan solutions up to 2% (*w*/*v*) were non-toxic to locusts when applied alone. Dose-response experiments with *M. acridum* conidia co-applied with mannan at 0.02%, 0.2%, or 2% identified 0.2% as optimal, reducing the LT_50_ by 0.64 days relative to fungus-only controls (*p* = 0.0382; Fig. 5b). To confirm that this strategy is broadly applicable across different entomopathogenic fungi, we tested the 0.2% mannan formulation in locusts with two other major entomopathogens. The approach was consistently effective, significantly shortening the LT_50_ for both *M. anisopliae* (by 0.40 days, *p* = 0.0082) and *B. bassiana* (by 0.63 days, *p* = 0.0105; Fig. 5c, d). These results establish that disrupting the IML1-SP28 checkpoint, either genetically or chemically, dramatically enhances fungal virulence by circumventing early immune detection.

Finally, we evaluated the broad-spectrum potential of this checkpoint disruption strategy by testing both genetic and chemical approaches against a diverse range of major agricultural pests. The KO-*ManSP28* mutant was also significantly more virulent against the fall armyworm *Spodoptera frugiperda* and the rice planthopper *Nilaparvata lugens*, as evidenced by a reduction in their respective LT_50_ values by 0.54 and 0.67 days (*p* < 0.05; Fig. 5e, f). We then used the generalist pathogen *M. anisopliae* to assess the efficacy of the 0.2% mannan formulation against a diverse range of agricultural pests using the more sensitive LD_50_ metric. The 0.2% mannan formulation significantly enhanced the potency of *M. anisopliae* against all tested species, evidenced by significant LD_50_ reductions against *L. migratoria* (2.27-fold, *p* = 0.0088), *S. frugiperda* (3.77-fold, *p* = 0.0032), *N. lugens* (5.89-fold, *p* = 0.0018), and a coleopteran pest, the flea beetle *Phyllotreta striolata* (a remarkable 8.26-fold, *p* = 0.0005; Fig. 5g–j). These consistent effects across four insect orders (Orthoptera, Lepidoptera, Hemiptera, Coleoptera) and multiple fungal species demonstrate that the IML1-SP28 checkpoint represents a broadly conserved immune mechanism, making it a promising target for enhancing biopesticide efficacy.

## Discussion

**Fig. 6:**
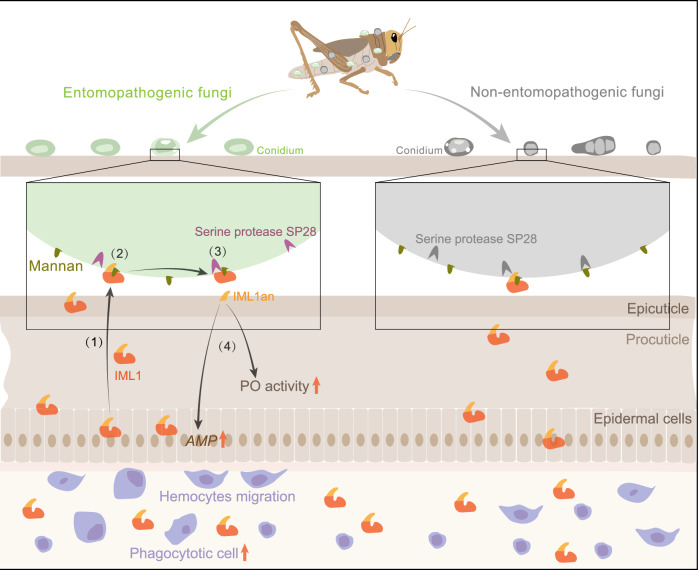
A model of locust IML1 discriminating pathogenic and non-pathogenic fungi on body surface. Schematic model illustrating the protease-gated immune discrimination system operating in four sequential steps: (1) Epidermal cells and hemocytes constitutively produce and secrete the lectin IML1 into the hemolymph, from which it translocates to the body surface through pore canals, establishing a surveillance layer on the epicuticle. (2) IML1 serves as a broad-spectrum sensor, binding to mannans present on both entomopathogenic (e.g., *Metarhizium*) and non-entomopathogenic fungi. However, this binding alone is insufficient to trigger immunity. (3) Pathogen-specific licensing occurs when serine protease SP28, secreted by entomopathogenic fungi, proteolytically cleaves mannan-bound IML1, releasing bioactive peptides (IML1an from *M. anisopliae*; IML1ac from *M. acridum*). In contrast, although non-entomopathogenic fungi secrete SP28 homologs, these proteases are functionally unable to cleave IML1, thereby failing to activate the checkpoint. (4) The liberated IML1-derived peptides penetrate through the cuticle to reach underlying epidermal cells, where they function as danger signals to mobilize systemic immune responses, including phenoloxidase (PO) activation in the cuticle, antimicrobial peptide (AMP) transcription in epidermal cells, and recruitment and activation of phagocytic hemocytes to the infection site. This two-checkpoint system, conserved pattern recognition (IML1-mannan binding) coupled with pathogen-specific licensing (SP28 cleavage), enables the host to distinguish genuine threats from harmless commensals at the critical host-environment interface.

Prevailing models of fungal immunity focus on responses triggered *after* a pathogen has breached the host surface. Here, we propose a *pre-emptive* surveillance system where the lectin IML1 acts as a gatekeeper on the insect body surface. By coupling a general pattern sensor (IML1 binding) with a pathogen-specific trigger (protease cleavage), the host exemplifies an evolutionary solution to a critical challenge: balancing rapid defense against pathogens with tolerance for commensals (Fig. 6). This shifts immune activation from inside the body to the outer surface, establishing a principle of insect *pre-emptive* defense at the host-environment interface.

The biphasic expression of IML1 further clarifies its role in this checkpoint (Supplementary Fig. 3a). An early transcriptional peak supports rapid protein recruitment to the cuticle surface for pre-invasion surveillance, while a later phase likely reinforces systemic defense during germination. Importantly, high IML1 expression in hemocytes is not contradictory to its surface function. Instead, it reflects a systemic mobilization strategy where IML1 is synthesized internally and transported to infection sites via pore canals. We propose that IML1 acts not as a static receptor, but as a soluble, dynamically replenished sentinel. Its amphipathic nature likely facilitates reversible interaction with the hydrophobic epicuticle, keeping it accessible to the fungal protease SP28. This ensures that peptide release into the hydrophilic procuticle is an immediate downstream consequence of pathogen contact.

This mechanism integrates two core immune principles: conserved pattern recognition and danger sensing. This integrated system may represent a conserved defense principle, with parallels in vertebrates and plants. For instance, surfactant proteins bind fungal PAMPs in mammalian lungs^32^ and in rice plants, they respond to fungal spores on the leaf surface^12^, triggering immune responses^33,34^. However, the mechanism preventing constant activation by non-pathogens has remained unclear. Our protease-gated model offers a solution. Given that IML1-like proteins and SP28 proteases are conserved across kingdoms, similar mechanisms may discriminate active threats from benign colonizers at other critical barriers, such as human skin or the plant phyllosphere. In this context, the loss of IML1-cleaving activity in non-entomopathogenic fungi likely reflects functional drift due to the absence of selective pressure to colonize insect cuticles.

SP28 is essential for fungal invasion but also triggers host immunity, creating a trade-off between rapid penetration and immune avoidance. Unlike dedicated elicitors such as Avr9^35^ or HYD3^14^, SP28 functions as a canonical virulence factor similar to *B. bassiana* Pr1^36^. However, our data reveal a crucial distinction: while deleting *SP28* partially impairs cuticle penetration, the mutant kills the host significantly faster in vivo. This phenotype reversal aligns with the stealth hypothesis in fungal pathogenesis, where evading immune surveillance is often a dominant determinant of hypervirulence^37^. In our model, the wild-type fungus is penalized by the host’s recognition of SP28, a mechanism analogous to the sensing of virulence factors in *Drosophila*^36^. Therefore, active immune clearance constitutes a stricter bottleneck to infection than the static physical barrier. While the deletion mutant struggles against the structural constraints of the cuticle (as evidenced by the inert wing assay), this invasion delay is negligible compared to the survival advantage gained by evading the host’s dynamic immune responses, such as melanization and antimicrobial peptide synthesis in the integument. By losing SP28, the mutant effectively adopts a stealth strategy, sacrificing penetration speed to bypass the immune checkpoint. Once the barrier is breached, the undetected mutant proliferates unchecked (as evidenced by the significantly higher fungal load in the hemolymph), demonstrating that avoiding immune detection confers a greater survival advantage than rapid entry in this specific interaction.

This dynamic traps the pathogen in an evolutionary trade-off. Mechanistically, SP28 is indispensable for breaching the nutrient-poor cuticle, making its retention non-negotiable for the fungus. However, the host has co-opted this essential activity as a reliable signature of attack. Thus, SP28 evolved as an invasion tool (the key), but inadvertently became the target of the host’s receptor (the lock). The pathogen cannot discard SP28 without losing its ability to invade, forcing it to retain a factor that betrays its presence.

Such reciprocal selection likely drives the tight host specificity observed in these interactions. We propose a co-evolutionary lock-and-key model where the fungal SP28 is molecularly tuned to a specific host receptor. Phylogenetic analysis supports this, showing intertwined evolutionary histories for insect/plant pathogens and their respective lectin targets. This provides a molecular basis for cross-kingdom pathogenicity, as seen in *Fusarium* species^38^. Our functional data confirm that the ability to cleave IML1 is restricted to SP28 variants from entomopathogens (Figs. 4g–i, 5a), proving that evolutionary relatedness does not guarantee functional equivalence. Specificity is therefore determined by fine-scale molecular adaptations within this checkpoint system.

Our findings have direct implications for antifungal strategies. In agriculture, blocking IML1 with mannan accelerates pest mortality, suggesting mannan could serve as a potent adjuvant for mycoinsecticides. This decoy strategy blinds the host’s surveillance system, allowing the fungus to bypass initial defenses and reducing the effective lethal dose. Beyond agriculture, this system highlights a class of danger signals: peptides released from surface receptors upon cleavage. Identifying the receptors for these peptides could reveal novel targets for therapeutic immune modulation. Furthermore, inhibitors targeting SP28 homologs in human pathogens like *Aspergillus* could potentially unmask these fungi to the patient’s immune system, offering a new avenue for antifungal therapy.

## Method

### Ethics statement

All research involving vertebrate animals complied with relevant ethical regulations and was approved by the Experimental Animal Ethics Committee of Chongqing University. Invertebrate insects do not require formal ethical approval under institutional guidelines.

### Insects and fungal strains

*Locusta migratoria*, flea beetle *Phyllotreta striolata*, fall armyworm *Spodoptera frugiperda*, and rice planthopper *Nilaparvata lugens* used in this study were reared at the Genetic Engineering Research Center of Chongqing University, China. The locusts were maintained at 30 ± 3 °C and 75% relative humidity with a 14h: 10 h light: dark photoperiod^14^. Rice planthoppers were reared on rice seedlings at 27 ± 1 °C, 80% relative humidity, and a 14 h: 10 h (light: dark) cycle^39^. Flea beetles were reared on cabbage at 28 ± 2 °C under a 14 h: 10 h (light: dark) photoperiod^40^. Fall armyworm eggs were collected from maize fields, and the resulting larvae were then individually reared on fresh maize leaves in a circular plastic box at 26 ± 2 °C, 65% relative humidity, and a 14 h: 10 h (light: dark) photoperiod^41^. Conidia from *Metarhizium acridum* CQMa102 strain, *M. anisopliae* CQMa421 strain, *Beauveria bassiana*, *Candida albicans*, *Aspergillus flavus*, and *Alternaria alternata* were provided by the Genetic Engineering Research Center of Chongqing University, China, and were cultured as previously described^14^. That is, fungal spores were inoculated onto 1/4SDAY medium and cultured at 28 °C. After 15 days, the conidia were harvested and used in experiments.

### Conidial affinity chromatograph

Collected conidia were suspended in a solution containing 50 mM sodium phosphate buffer, 100 mM NaCl, 0.5% Tween-80, and 0.5% formaldehyde, and fixed for 30 min at 4 °C. The fixed conidia were washed three times in 50 mM phosphate buffer saline (PBS) containing 8 M urea, pH 7.5, and centrifuged at 6000 × *g* at 4 °C. The pellet was suspended in 50 mM PBS supplemented with 100 mM NaCl and 0.5% Tween-80. Sampled hindwings, cuticles, and cell-free hemolymph were collected from locusts inoculated with *M. acridum* conidia four hours earlier. Protein extracts were prepared from grinded tissues in 50 mM PBS supplemented with 100 mM NaCl, 10% glycerol, pH 7.5. The homogenate was centrifuged at 4 °C for 13,200 × *g*, and the supernatants incubated with conidia at 4 °C overnight. Unbound proteins were eliminated by centrifugation for 13,200 × *g* at 4 °C, and the conidia were wash three times in 50 mM PBS, 100 mM NaCl, 10% glycerol, pH 7.5, and twice in 50 mM PBS, 500 mM NaCl, 10% glycerol, pH 7.5. The bound proteins were eluted with 50 mM PBS, 8 M urea, pH 7.5 and the eluates were analyzed by SDS-PAGE.

### Liquid chromatography-tandem mass spectrometry (LC-MS/MS) analysis

Protein samples were reduced with a final concentration of 10 mM dithiothreitol (DTT) and subsequently alkylated with 55 mM iodoacetamide (IAM). The proteins were then digested with 1 μg trypsin at 37 °C for 8–16 h. The resulting peptides were desalted using C18 columns, dried under vacuum, and reconstituted in 15 μL of loading buffer (0.1% formic acid and 3% acetonitrile). Peptide analysis was performed using an Ekspert™ nanoLC system coupled to an AB Sciex TripleTOF 5600-plus mass spectrometer. Raw mass spectrometry data were processed and assessed using ProteinPilot software Version 5.0.1 (AB Sciex) against a custom locust protein, with trypsin as the enzyme, iodoacetamide as the cysteine alkylation agent, and the search set to thorough ID with biological modifications. The search was performed against a custom locust protein database (Locust.V2.4.1.prot.fasta, containing 17,586 sequences). Peptide and protein identifications were filtered at a 1% false discovery rate (FDR).

### Phenoloxidase (PO) activity assay

Fifth-instar nymphs were topically inoculated on the second and third abdominal segments with 5 μL of conidia (1 × 10^8^ conidia/mL) suspended in adhesion buffer (0.5% Tween-80, 5% glycerol, 2% PEG-10000). At 4 h post-inoculation, the cuticle was dissected from the inoculation sites. After grinding in liquid nitrogen, total proteins were extracted in 50 mM PBS (containing 100 mM NaCl, pH 6.5). L-DOPA was used as a substrate, cuticle proteins were added, and the mixtures incubated at 28 °C. The absorbance of the sample was measured at 5 and 35 min, respectively. One unit of PO activity was defined as the change in absorbance at 485 nm each one min, which equals 0.001 absorbance units per minute^14^.

### Total hemocytes and phagocytic cells quantification

The method has been previously described^42^. Ten-microliter hemolymph sample was collected from locusts, and was spread on a slide to produce a smear and was dried at 37 °C for 20 min. Each slide was stained in Wright Stain (Sangon, China) for 8 min at 28 °C, followed by the application of 0.1 ml Giemsa stain (Sangon, China). The slides were incubated at 28 °C for 8 min and then were rinsed with water. After air drying, the hemocyte smear was inspected under a microscope.

### RNA isolation and qRT-PCR

Fat bodies, hemocytes, and epidermal cells were collected after treatment^43^. Total RNA was extracted with an Ultrapure RNA kit (CWbiotech, China), and the complementary DNA (cDNA) was synthesized. Quantitative real-time PCR (RT-qPCR) and the data analysis were performed according to a previously described method^44^. All primers were commercially synthesized by Tsingke Biotechnology (Beijing, China), and their specific sequences are listed in Supplementary Data 2.

### RNAi (RNA interference)

A target sequence for dsRNA-mediated *IML1* gene knockdown was designed based on a validated strategy^43^. To prepare the transcription templates, target-specific fragments flanked by T7 promoters were amplified by PCR using primers synthesized by Tsingke Biotechnology (Beijing, China). *IML1*-specific dsRNA and *GFP* dsRNA were synthesized using the MEGAscript high-yield transcription kit (Ambion, Austin, TX, USA), according to the manufacturer’s protocol. *GFP* was used as a negative control, as *GFP* dsRNA does not display any side effects and has been broadly used in locusts^45^. Briefly, a dsRNA template was prepared by PCR and purified before dsRNA synthesis. dsRNA was synthesized by mixing the plasmid template and in vitro transcription reagents in an RNase-free tube. After transcription, the DNA template was digested by DNase. The dsRNA product was precipitated in 1 M lithium chloride solution. Final dsRNA concentration was determined using a NanoVue Plus spectrophotometer (GE Health-care Life Sciences, Little Chalfont, UK). For the in vivo interference assay, 5 μL of a dsRNA solution at a total of 2 μg was injected per locust into the hemocoel, through the abdomen. The primer sequences for dsRNA synthesis are provided in Supplementary Data 2.

### Recombinant protein expression and purification

The complete gene sequence of *IML1* and SP28 (removing the signal peptide) were subcloned into pET28a (#V011005, NovoPro, China) or pCold-MBP (#V012984, NovoPro, China) vectors, respectively, and expressed in *Escherichia coli*^14^. The transformants were inoculated into 200 mL of LB broth containing 100 μg/mL ampicillin. The cells were incubated at 37 °C with shaking at 200 rpm. When the culture had reached an optical density OD of 0.6–0.8 at 600 nm, protein expression was induced with a final concentration of 0.5 mM isopropyl β-D-1-thiogalactopyranoside (IPTG) at 18 °C for 12 h. The cells were then collected by centrifugation at 13,200 × *g* for 10 min at 4 °C, and the supernatant was resuspended in 50 mL of binding buffer (10 mM HEPES, 150 mM NaCl, 25 mM imidazole, pH 7.5), and disrupted by ultrasonication. The cell debris was removed by centrifugation at 13,200 × *g* for 20 min at 4 °C, and the crude supernatant containing rIML1 was collected. The lysate was filtered through a 0.22 μm nylon membrane and loaded onto an Ni^2+^-affinity chromatography column. The recombinant protein was eluted with 40 mL of elution buffer (10 mM HEPES, 150 mM NaCl, 300 mM imidazole, pH 7.5). The purified IML1 was assessed using SDS-PAGE and Coomassie blue staining. Primers used for vector construction are listed in Supplementary Data 2.

### Antibody preparation

Polyclonal antibodies were prepared in five BALB/c female mice using the purified recombinant IML1 or an AfSP28 fragment protein as an antigen^14^. For antigen production, the high specificity fragment was subcloned into the pET32a vector (#V010987, NovoPro, China), expressed in *E. coli*, and purified. BALB/c female mice (6–8 weeks old) used for polyclonal antibody production were housed under specific pathogen‑free conditions at the Experimental Animal Center of Chongqing University, with a 12 h light: 12 h dark cycle, ambient temperature of 22 ± 2 °C, and relative humidity of 50 ± 5%. Animals were provided with standard chow and water ad libitum. These animals were immunized four times at two weeks intervals. For the first time the animals were injected with a mixture of antigen protein and complete Freund’s adjuvant (1:1, v/v). The following injections used the protein antigen in incomplete Freund’s adjuvant (1:1, v/v). Serum titer was measured by an indirect enzyme-linked immunosorbent assay (ELISA). The recombinant antigen was coated to wells. Bleedings were carried out two weeks after the last injection, and blood samples were allowed to coagulate for 2 h at 37 °C, plus 1 h at 4 °C. They were then centrifuged at 3000 × *g* for 15 min at 4 °C. The polyclonal antiserum was then purified using Protein A/G affinity chromatography to obtain the antibody used in all subsequent experiments. The purified antibody was aliquoted and stored at − 80 °C. Primers used for vector construction are listed in Supplementary Data 2.

### Protein extraction, SDS-PAGE, and western blotting

Total proteins were extracted from cuticle, epidermal cells, fat body, and hemocytes using RIPA buffer (50 mM Tris-HCl, 150 mM NaCl, 0.1% sodium deoxycholate, 1% Nonidet P40, 1 mM PMSF, 4 mM EDTA, pH 8.0)^46^. The protein solution was centrifuged at 13,200 × *g* for 10 min and the supernatants were collected. The protein concentration in the supernatants was determined using BCA kit (#P0009, Beyotime, China), and sample aliquots were adjusted to ensure uniform loading across all lanes. Subsequently, 100 μg of protein per lane was loaded and separated using 12% polyacrylamide gel under 120 V for 80 min, and transferred to a PVDF membrane (Life Sciences). After blocking with 3% skimmed milk, the primary antibody or anti-serum was diluted to 1:1000 and incubated overnight at 4 °C. The HRP-conjugated goat anti-mouse secondary antibody (#A0216, Beyotime, China) was used to detect the primary antibody at a dilution of 1:12,000 and was visualized using chemiluminescence (Beyotime, China) in a Transilluminator (TAKARA, Japan).

### Extraction of total surface proteins and in vitro cleavage assay

Fungal spores were suspended in PBS (pH 7.4) containing 0.05% NP-40, and incubated on ice for 30 min, gently resuspending every 5 min to ensure homogeneity^47^. Then, they were centrifuged at 13,200 × *g* for 10 min to pellet the spores. The supernatant was transferred to a new centrifuge tube. It was aliquoted, and each aliquot was supplemented with glycerol to a final concentration of 10%. The aliquoted samples were stored at − 80 °C for long-term preservation. For the cleavage assay, 5 μg of rIML1 was incubated with 0.1 μg of conidial surface proteins or 0.05 μg of rSP28 in reaction buffer (50 mM Tris-HCl, 100 mM NaCl, pH 7.5) at 37 °C for 2 h. Where indicated, 1 mM PMSF was added. Cleavage products were analyzed by SDS-PAGE.

### Fluorescence in situ hybridizations (FISH) and immunostainings

FITC-labeled RNA probes complementary to *IML1* mRNA were synthesized, and FISH was performed^48^. Briefly, the locust integument was sectioned and immersed in PBS, pH 7.2, containing 2.5% glutaraldehyde. The integument was then dehydrated using a series of gradient ethanol solutions and subsequently infiltrated with melted paraffin. The sections were blocked in Western Blocking Reagent (#11921673001, Merck, Germany). Following overnight hybridization, samples were incubated with RNA probes and washed twice with PBS containing 0.5% Triton X-100. For immunostaining, the slides were treated with an anti-IML1 antibody and an Alexa Fluor 594 -labeled secondary antibody (#A-11005, Thermo Fisher, USA) at 4 °C overnight. For the detection of surface mannan using Mannan-Binding Lectin (MBL), fungal conidia were first incubated with MBL (#P70674, MedChemExpress, China), followed by incubation with a rabbit polyclonal anti-MBL2 antibody (#AF7428, Beyotime, China) and subsequently with a FITC-conjugated goat anti-rabbit IgG secondary antibody (#A0562, Beyotime, China). For immunofluorescence detection of FLAG-tagged proteins on fungal conidia, samples were incubated with a mouse monoclonal anti-FLAG antibody (#AF2852, Beyotime, China) followed by an Alexa Fluor 594-conjugated goat anti-mouse IgG secondary antibody (#A-11005, Thermo Fisher, USA). The slides were washed four times with PBS containing 0.1% Tween-20. All FISH and immunostainings images were captured using an ELIPSE Ci fluorescence microscopy (Nikon, Japan). Quantification of fluorescence intensity on fungal spores was performed using ImageJ (version 1.51j8). The sequences of FISH probes are provided in Supplementary Data 2.

### Immunogold staining and scan electron microscopy

Cuticle samples with attached conidia were dissected from the second and third abdominal segments^49^. The epidermal cell layer was carefully scraped off using a scalpel blade until the cuticle became translucent. The sample was then mounted on a slide with the outer surface facing upward and immediately fixed by adding PBS (pH 7.2) containing 2.5% glutaraldehyde and 0.5% Tween-20. The fixed samples were blocked with 1% bovine serum albumin (BSA) in TBST (Tris-buffered saline with 0.1% Tween-20) for 1 h at room temperature to minimize nonspecific binding. For immunogold detection of IML1, after blocking, samples were incubated with the primary mouse anti-IML1 antiserum (diluted in 1% BSA TBST), followed by thorough washing with 1% BSA TBST and subsequent incubation with a goat anti-mouse secondary antibody conjugated to 35 nm colloidal gold particles (K1031R, Solarbio, China) diluted in 1% BSA TBST for 2 h at room temperature. After a final rinse, samples were processed for SEM without conductive coating. Imaging was performed on a Field Emission Scanning Electron Microscope SU8600 (Hitachi, Japan) in CSS mode, using a working distance of 3 mm, an accelerating voltage of 1 kV, and a probe current of 5 μA. Under these imaging conditions, the high-density colloidal gold particles, due to their strong electron scattering, appear as bright white spots against the cuticle background. The diameter of these spots was measured to be 35 nm, confirming their identity as the immunogold labels. This method enables nanoscale protein localization on uncoated biological samples. A control for nonspecific staining was performed by substituting the primary anti-IML1 antiserum with normal mouse IgG, which resulted in no detectable gold particles on the cuticle surface.

### SunTag validation of IML1 secretion

The SunTag system^25^ was employed to visualize the translocation of IML1 from the hemolymph to the cuticle surface. Recombinant IML1 fused with 12 × GCN4 peptide array and ScFv-GFP were respectively cloned into pET28a and expressed in *E. coli* BL21(DE3). Protein expression was induced with 0.1 mM IPTG at 18 °C for 12 h. Proteins were purified using Ni^2+^-affinity chromatography as described above, dialyzed against 10 mM PBS (pH 7.2), and verified by SDS-PAGE. Fifth-instar locust nymphs were injected with 5 μL purified IML1-12 × GCN4 (2 μg/μL) into the hemocoel. Twelve hours later, *M. acridum* conidia (5 μL, 1 × 10^8^ conidia/mL in 0.05% Tween-80) were topically applied to the abdominal cuticle. At 0, 2, and 4 h post-inoculation, cuticles with attached conidia were dissected (basal membrane and epidermal cells removed), fixed in 4% paraformaldehyde for 30 min, and washed with PBST. Samples were blocked with 3% BSA, incubated with ScFv-GFP (10 μg/mL) overnight at 4 °C, washed four times with PBST, and imaged by fluorescence microscopy. PBS-injected locusts or ScFv-GFP alone served as controls. Primers used for SunTag vector construction are listed in Supplementary Data 2.

### Construction of fungal mutant strains

Targeted gene manipulation was performed via *Agrobacterium tumefaciens*-mediated transformation^50^. The *SP28* deletion mutant was generated by replacing the coding sequence with the phosphinothricin resistance (bar) gene via homologous recombination. Overexpression (OE) and heterologous knock-in (KI) strains, expressing C-terminal FLAG-tagged native or orthologous *SP28* (*BbSP28* or *AfSP28*), were constructed by replacing the native promoter with the constitutive *gpdA* promoter or replacing the native gene in situ, respectively, using bar as the selectable marker. Complementation strains were generated by ectopic insertion using the chlorimuron-ethyl resistance (*sur*) gene. For heat-inactivation, conidial suspensions (1 × 10^8^ conidia/mL) were incubated at 100 °C for 20 min. The primer sequences for mutant construction and verification are listed in Supplementary Data 2.

### Fungal fitness assays

Locust hindwings dissected from adult locusts were rinsed three times with sterile water and sterilized by autoclaving^19^. For the penetration assay, sterilized hindwings were placed flat on 1/4 SDAY medium. Conidial suspensions (2 μL, 1 × 10^7^ conidia/mL) of *M. anisopliae* wild-type (WT), KO-*ManSP28*, or complemented strain (CP) were spot-inoculated onto the hindwing surface. After 3 days of incubation at 28 °C, hindwings were photographed and carefully removed from the medium. Plates were then incubated for an additional 3 days to allow fungi that had penetrated the cuticle to continue growing on the medium. Colony diameter was measured and photographed. For direct growth comparison, 3 μL of each conidial suspension was spot-inoculated directly onto 1/4 SDAY medium without hindwings. Colony diameter was measured after 3 days of incubation at 28 °C. Each treatment included at least three replicates, and the experiment was repeated three times.

### Small peptide purification

The recombinant IML1 was incubated with recombinant SP28 at 28 °C for 30 min^51^. The resulting mixture was resolved by Native-PAGE. Protein bands were visualized by negative staining with a 3 M KCl solution, and the gel slice containing the target peptide band was excised. The excised gel piece was thoroughly ground, and the peptide was eluted by suspension in 50 mM PBS (containing 100 mM NaCl, pH 7.5). After elution, the gel debris was pelleted by centrifugation, and the supernatant was collected. The peptide was further purified using an ultrafiltration centrifugal device with a 10 kDa molecular weight cutoff. The flow-through, which contained the purified peptide, was collected. The concentration of the purified peptide was determined using a BCA protein assay kit, according to the manufacturer’s instructions. The peptide solution was then aliquoted and stored at −80 °C until use. For subsequent immune assays, an aliquot was thawed and diluted to the desired working concentration in PBS.

### Molecular docking analysis

The structure of IML1 was predicted using RoseTTAFold (https://robetta.bakerlab.org/submit.php)^52^. The structures of the three glycans were obtained from PubChem (https://pubchem.ncbi.nlm.nih.gov/). The binding affinity and interaction sites of IML1 with mannan, glucan, and chitin were assessed using molecular docking analysis conducted with AutoDock v.4.2^53^. The default parameters were used for the docking analysis, and the grid boxes were designed to completely encompass the binding sites of the glycans. The lowest binding free energy values corresponding to the active cavities of IML1 were selected for further validation of the docking conformations.

### Determination of binding affinity by Enzyme-linked immunosorbent assay

The binding affinity of IML1 for mannan (#M7504, Sigma-Aldrich, Germany), glucan (#L9634, Sigma-Aldrich, Germany), and chitin (#C7170, Sigma-Aldrich, Germany) was determined by competitive assays according to procedures previously described^54^. Briefly, the wells of the ELISA plate were coated with IML1 and blocked with 3% BSA (dissolved in phosphate buffer saline containing 0.5% Tween-20, PBST). A series of graded concentrations of FITC-labeled glycans were added to different wells. Unlabeled glycans were added to compete with FITC-labeled glycans for IML1 binding. The wells were washed three times with PBST, and the fluorescent intensity was measured in a microplate reader. The binding affinity (Kd) was calculated in Prism GraphPad software.

### Phylogenetic analysis

Homologous sequences of IML1 and SP28 were retrieved from NCBI and aligned using MUSCLE. Phylogenetic trees were constructed using MEGA 12.0.14 based on the Neighbor-Joining (NJ) method with 1000 bootstrap replicates. The accession numbers of all sequences used for phylogenetic analysis are listed in Supplementary Table 1 and Supplementary Data 3.

### Survival assays

Survival assays were performed using fifth-instar nymphs of *L. migratoria*, second-instar nymphs of *Spodoptera frugiperda*, adults of *Phyllotreta striolata*, and adults of *Nilaparvata lugens*.

For the KO-*ManSP28* virulence assay, 5 µL of a paraffin oil suspension containing KO-*ManSP28* conidia at concentrations of 4 × 10,^5^ 2 × 10,^6^ 1 × 10,^7^ 5 × 10,^7^ and 2.5 × 10^8^ conidia/mL was applied to the head-thorax junction of each locust^14^. The *S. frugiperda* and *N. lugens* were sprayed with an aqueous suspension of KO-*ManSP28* conidia (1 × 10^7^ conidia/mL) containing 0.1% Tween-80, then maintained at 90% relative humidity for 48 h^39,41^.

For virulence assessment of co-applying mannan, the *M. anisopliae* conidia were suspended in aqueous solution (0.1% Tween-80 plus 0.2% mannan) at multiple concentrations (4 × 10,^5^ 2 × 10,^6^ 1 × 10,^7^ 5 × 10,^7^ and 2.5 × 10^8^ conidia/mL) and applied topically (5 µL) to the head-thorax junction. The *M. acridum* and *B. bassiana* conidia were similarly suspended in 0.1% Tween-80 plus 0.2% mannan solution at 1 × 10^7^ conidia/mL for topical application. The *M. anisopliae* conidia suspended in 0.1% Tween-80/0.2% mannan solution (1 × 10^7^ conidia/mL) were sprayed to *P. striolata*^6^. Correspondingly, control groups received either paraffin oil or 0.1% Tween-80 aqueous solution at equivalent volumes. The controls were treated with the same amount of paraffin oil, or 0.1% tween-80 aqueous solution, respectively. For each treatment, 30 insects in per group, and the experiment was repeated three times. Mortality was monitored at 12 h intervals.

### Statistical analysis

All statistical analyses were performed using SPSS (Version 20.0.0, IBM Corp.). All data are presented as mean ± standard error of the mean (SEM) from at least three independent biological replicates. A *P*-value < 0.05 was considered statistically significant. Specific statistical tests were applied as follows: For comparisons between two groups, a two-sided Student’s *t* test was used. For comparisons involving more than two groups, a one-way analysis of variance (ANOVA) was performed, followed by Tukey’s honestly significant difference (HSD) post-hoc test for pairwise comparisons. Survival data were analyzed using Kaplan-Meier survival curves, and differences between groups were assessed with the log-rank (Mantel-Cox) test. The median lethal time (LT_50_) for each group was estimated from the Kaplan-Meier curves. For the determination of the median lethal dose (LD_50_), mortality data from dose-response experiments were analyzed using a Probit regression model.

### Reporting summary

Further information on research design is available in the Nature Portfolio Reporting Summary linked to this article.

## Supplementary information

Supplementary information

Description of Additional Supplementary Files

Supplementary Data 1

Supplementary Data 2

Supplementary Data 3

Reporting Summary

Transparent Peer Review file

## Source data

Source Data

## Data Availability

The protein mass spectrometry raw data have been deposited to the ProteomeXchange Consortium via the iProX partner repository with the dataset identifier PXD076064. All other data supporting the findings of this study are available within the Article, the Supplementary Information, or the Source Data file. The full list of identified proteins from the pull-down assay is provided in Supplementary Data 1. Primer and probe sequences are provided in Supplementary Data 2, and gene accession numbers used for phylogenetic analysis are listed in Supplementary Data 3. All newly generated materials, including the recombinant plasmids, expression vectors, and fungal mutant strains described in this study, are available from the corresponding author upon reasonable request for non-commercial research purposes. Source data are provided in this paper.

## References

[1] Heald, C. L. & Spracklen, D. V. Atmospheric budget of primary biological aerosol particles from fungal spores. *Geophys. Res. Lett.* **36**, L09806 (2009).

[2] Woo, C., An, C., Xu, S., Yi, S.-M. & Yamamoto, N. Taxonomic diversity of fungi deposited from the atmosphere. *ISME J.* **12**, 2051–2060 (2018). 29849168 10.1038/s41396-018-0160-7 PMC6051994

[3] Limon, J. J., Skalski, J. H. & Underhill, D. M. Commensal fungi in health and disease. *Cell Host Microbe* **22**, 156–165 (2017). 28799901 10.1016/j.chom.2017.07.002 PMC5573128

[4] Zhou, J. & Zhang, Y. Plant immunity: danger perception and signaling. *Cell* **181**, 978–989 (2020). 32442407 10.1016/j.cell.2020.04.028

[5] Lionakis, M. S., Drummond, R. A. & Hohl, T. M. Immune responses to human fungal pathogens and therapeutic prospects. *Nat. Rev. Immunol.* **23**, 433–452 (2023). 36600071 10.1038/s41577-022-00826-w PMC9812358

[6] Buchon, N., Silverman, N. & Cherry, S. Immunity in Drosophila melanogaster — from microbial recognition to whole organism physiology. *Nat. Rev. Immunol.* **14**, 796–810 (2014). 25421701 10.1038/nri3763 PMC6190593

[7] Ruchti, F. & LeibundGut-Landmann, S. New insights into immunity to skin fungi shape our understanding of health and disease. *Parasite Immunol.* **45**, e12948 (2023). 36047038 10.1111/pim.12948 PMC10078452

[8] Vance, R. E., Isberg, R. R. & Portnoy, D. A. Patterns of pathogenesis: discrimination of pathogenic and nonpathogenic microbes by the innate immune system. *Cell Host Microbe* **6**, 10–21 (2009). 19616762 10.1016/j.chom.2009.06.007 PMC2777727

[9] Sparber, F. et al. The skin commensal yeast *Malassezia* triggers a type 17 response that coordinates anti-fungal immunity and exacerbates skin inflammation. *Cell Host Microbe* **25**, 389–403 (2019). 30870621 10.1016/j.chom.2019.02.002

[10] Schultz, T. R. et al. The coevolution of fungus-ant agriculture. *Science* **386**, 105–110 (2024). 39361762 10.1126/science.adn7179

[11] Berasategui, A. et al. The leaf beetle *Chelymorpha alternans* propagates a plant pathogen in exchange for pupal protection. *Curr. Biol.* **32**, 4114–4127 (2022). 35987210 10.1016/j.cub.2022.07.065

[12] Li, W. et al. The durably resistant rice cultivar Digu activates defence gene expression before the full maturation of *Magnaporthe oryzae* appressorium. *Mol. Plant Pathol.* **17**, 354–368 (2015). 26095454 10.1111/mpp.12286 PMC6638526

[13] Huang, X. et al. Murine model of colonization with fungal pathogen *Candida auris* to explore skin tropism, host risk factors and therapeutic strategies. *Cell Host Microbe* **29**, 210–221 (2021). 33385336 10.1016/j.chom.2020.12.002 PMC7878403

[14] Jiang, Z. Y., Ligoxygakis, P. & Xia, Y. X. HYD3, a conidial hydrophobin of the fungal entomopathogen *Metarhizium acridum* induces the immunity of its specialist host locust. *Int. J. Biol. Macromol.* **165**, 1303–1311 (2020). 33022346 10.1016/j.ijbiomac.2020.09.222

[15] Kawai, T., Ikegawa, M., Ori, D. & Akira, S. Decoding Toll-like receptors: Recent insights and perspectives in innate immunity. *Immunity* **57**, 649–673 (2024). 38599164 10.1016/j.immuni.2024.03.004

[16] Rao, X. J. et al. Immune functions of insect βGRPs and their potential application. *Dev. Comp. Immunol.* **83**, 80–88 (2018). 29229443 10.1016/j.dci.2017.12.007

[17] Tan, X. et al. A pair of LysM receptors mediates symbiosis and immunity discrimination in Marchantia. *Cell* **188**, 1330–1348 (2025). 39855200 10.1016/j.cell.2024.12.024

[18] Gow, N. A. R., Latge, J. P., Munro, C. A. & Heitman, J. The fungal cell wall: structure, biosynthesis, and function. *Microbiol. Spectr.* **5**, FUNK-0035–FUNK-2016 (2017). 10.1128/microbiolspec.funk-0035-2016 PMC11687499 28513415

[19] Du, Y. R., Li, J., Chen, S. P., Xia, Y. X. & Jin, K. Pathogenicity analysis and comparative genomics reveal the different infection strategies between the generalist *Metarhizium anisopliae* and the specialist *Metarhizium acridum*. *Pest Manag. Sci.* **80**, 820–836 (2023). 37794279 10.1002/ps.7812

[20] Kim, B. Y. & Jin, B. R. The dual roles of *Bombyx mori* immulectin in cuticular melanization and innate immunity. *J. Asia Pac. Entomol.* **20**, 761–766 (2017).

[21] Ochiai, M. & Ashida, M. A pattern-recognition protein for beta-1,3-glucan. The binding domain and the cDNA cloning of beta-1,3-glucan recognition protein from the silkworm, *Bombyx mori*. *J. Biol. Chem.* **275**, 4995–5002 (2000). 10671539 10.1074/jbc.275.7.4995

[22] Netea, M. G., Brown, G. D., Kullberg, B. J. & Gow, N. A. An integrated model of the recognition of *Candida albicans* by the innate immune system. *Nat. Rev. Microbiol.* **6**, 67–78 (2008). 18079743 10.1038/nrmicro1815

[23] Garcia-Rubio, R., de Oliveira, H. C., Rivera, J. & Trevijano-Contador, N. The fungal cell wall: *Candida*, *Cryptococcus*, and *Aspergillus* species. *Front. Microbiol.* **10**, 2993 (2020). 31993032 10.3389/fmicb.2019.02993 PMC6962315

[24] Zheng, X. L., Li, S., Si, Y., Hu, J. & Xia, Y. X. Locust can detect beta-1, 3-glucan of the fungal pathogen before penetration and defend infection via the Toll signaling pathway. *Dev. Comp. Immunol.* **106**, 103636 (2020). 32014469 10.1016/j.dci.2020.103636

[25] Tanenbaum, M. arvinE., Gilbert, L. ukeA., Qi, L. eiS., Weissman, J. onathanS. & Vale, R. onaldD. A protein-tagging system for signal amplification in gene expression and fluorescence imaging. *Cell* **159**, 635–646 (2014). 25307933 10.1016/j.cell.2014.09.039 PMC4252608

[26] Nakatogawa, S. et al. A novel peptide mediates aggregation and migration of hemocytes from an insect. *Curr. Biol.* **19**, 779–785 (2009). 19375321 10.1016/j.cub.2009.03.050

[27] Gunnarsson, S. G. S. Infection of *Schistocerca gregaria* by the fungus *Metarhizium anisopliae*: cellular reactions in the integument studied by scanning electron and light microscopy. *J. Invertebr. Pathol.* **52**, 9–17 (1988).

[28] Yike, I. Fungal proteases and their pathophysiological effects. *Mycopathologia* **171**, 299–323 (2011). 21259054 10.1007/s11046-010-9386-2

[29] Madan, T. & Kishore, U. Surfactant protein D recognizes multiple fungal ligands: A key step to initiate and intensify the anti-fungal host defense. *Front. Cell Infect. Microbiol.* **10**, 229 (2020). 32547959 10.3389/fcimb.2020.00229 PMC7272678

[30] Zhou, J. et al. Transcriptome analysis and functional characterization reveal that Peclg gene contributes to the virulence of *Penicillium expansum* on Apple fruits. *Foods* **12**, 479 (2023). 36766008 10.3390/foods12030479 PMC9914705

[31] Spatafora, J. W., Sung, G. H., Sung, J. M., Hywel-Jones, N. L. & White, J. F. Jr Phylogenetic evidence for an animal pathogen origin of ergot and the grass endophytes. *Mol. Ecol.* **16**, 1701–1711 (2007). 17402984 10.1111/j.1365-294X.2007.03225.x

[32] Han, S. & Mallampalli, R. K. The role of surfactant in lung disease and host defense against pulmonary infections. *Ann. Am. Thorac. Soc.* **12**, 765–774 (2015). 25742123 10.1513/AnnalsATS.201411-507FR PMC4418337

[33] Wright, J. R. Immunoregulatory functions of surfactant proteins. *Nat. Rev. Immunol.* **5**, 58–68 (2005). 15630429 10.1038/nri1528

[34] Wang, Y. & Bouwmeester, K. L-type lectin receptor kinases: New forces in plant immunity. *PLoS Pathog.* **13**, e1006433 (2017). 28817713 10.1371/journal.ppat.1006433 PMC5560540

[35] Van den Ackerveken, G. F. J. M., Van Kan, J. A. L. & De Wit, P. J. G. M. Molecular analysis of the avirulence gene avr9 of the fungal tomato pathogen *Cladosporium fulvum* fully supports the gene-for-gene hypothesis. *Plant J.* **2**, 359–366 (2005). 10.1111/j.1365-313x.1992.00359.x 1303800

[36] Gottar, M. et al. Dual detection of fungal infections in *Drosophila* via recognition of glucans and sensing of virulence factors. *Cell* **127**, 1425–1437 (2006). 17190605 10.1016/j.cell.2006.10.046 PMC1865096

[37] Wang, C. S. & St. Leger, R. J. A collagenous protective coat enables *Metarhizium anisopliae* to evade insect immune responses. *Proc. Natl. Acad. Sci. USA* **103**, 6647–6652 (2006). 16614065 10.1073/pnas.0601951103 PMC1458935

[38] Santos, A. C. D. S., Diniz, A. G., Tiago, P. V. & de Olivrira, N. T. Entomopathogenic Fusarium species: a review of their potential for the biological control of insects, implications and prospects. *Fungal Biol. Rev.* **34**, 41–57 (2020).

[39] Tang, C. et al. The symbiont *Acinetobacter baumannii* enhances the insect host resistance to entomopathogenic fungus *Metarhizium anisopliae*. *Commun. Biol.* **7**, 1184 (2024). 39300313 10.1038/s42003-024-06779-1 PMC11412983

[40] Li, J. Y. et al. Infection of *Metarhizium anisopliae* Ma6 and defense responses of host *Phyllotreta striolata* adults. *Arch. Insect. Biochem.* **110**, e21908 (2022). 10.1002/arch.21908 35470484

[41] Zheng, R. W. et al. Comparative analysis of gut microbiota and immune genes linked with the immune system of wild and captive *Spodoptera frugiperda* (Lepidoptera: Noctuidae). *Dev. Comp. Immunol.* **138**, 104530 (2023). 36084754 10.1016/j.dci.2022.104530

[42] Yu, Y., Cao, Y. Q., Xia, Y. X. & Liu, F. H. Wright-Giemsa staining to observe phagocytes in Locusta migratoria infected with *Metarhizium acridum*. *J. Invertebr. Pathol.* **139**, 19–24 (2016). 27345377 10.1016/j.jip.2016.06.009

[43] Zhang, W. et al. An odorant binding protein is involved in counteracting detection-avoidance and Toll-pathway innate immunity. *J. Adv. Res.* **48**, 1–16 (2023). 36064181 10.1016/j.jare.2022.08.013 PMC10248801

[44] Zhang, W. et al. Comparative transcriptomic analysis of immune responses of the migratory locust, *Locusta migratoria*, to challenge by the fungal insect pathogen, *Metarhizium acridum*. *BMC Genom.* **16**, 867 (2015). 10.1186/s12864-015-2089-9 PMC4624584 26503342

[45] Wang, Y. D. et al. Variation of TNF modulates cellular immunity of gregarious and solitary locusts against fungal pathogen *Metarhizium anisopliae*. *Proc. Natl. Acad. Sci. USA* **119**, e2120835119 (2022). 35110413 10.1073/pnas.2120835119 PMC8833202

[46] Kang, X. L., Yang, M. L., Cui, X. S., Wang, H. M. & Kang, L. Spatially differential regulation of ATF2 phosphorylation contributes to warning coloration of gregarious locusts. *Sci. Adv.* **9**, eadi5168 (2023). 37611100 10.1126/sciadv.adi5168 PMC10446495

[47] Yang, Z. et al. Correlation of cell surface proteins of distinct *Beauveria bassiana* cell types and adaption to varied environment and interaction with the host insect. *Fungal Genet. Biol.* **99**, 13–25 (2017). 28040530 10.1016/j.fgb.2016.12.009

[48] Scimone, M. L., Cloutier, J. K., Maybrun, C. L. & Reddien, P. W. The planarian wound epidermis gene equinox is required for blastema formation in regeneration. *Nat. Commun.* **13**, 2726 (2022). 35585061 10.1038/s41467-022-30412-6 PMC9117669

[49] Miller, K. K., Wang, P. & Grillet, N. SUB-immunogold-SEM reveals nanoscale distribution of submembranous epitopes. *Nat. Commun.* **15**, 7864 (2024). 39256352 10.1038/s41467-024-51849-x PMC11387508

[50] Qin, Y. P. & Xia, Y. X. A negative melanin regulator, Mamrn, regulates thermo and UV tolerance via distinct mechanisms in *Metarhizium*. *Microbiol. Res.* **297**, 128190 (2025). 10.1016/j.micres.2025.128190 40300370

[51] Wang, B. et al. Purification and characterisation of a novel antioxidant peptide derived from blue mussel (*Mytilus edulis*) protein hydrolysate. *Food Chem.* **138**, 1713–1719 (2013). 23411302 10.1016/j.foodchem.2012.12.002

[52] Baek, M. et al. Accurate prediction of protein structures and interactions using a three-track neural network. *Science* **373**, 871–876 (2021). 34282049 10.1126/science.abj8754 PMC7612213

[53] Morris, G. M. et al. Autodock4 and AutoDockTools4: automated docking with selective receptor flexiblity. *J. Comput. Chem.* **16**, 2785–2791 (2009). 10.1002/jcc.21256 PMC2760638 19399780

[54] Rath, S., Stanley, C. M. & Steward, M. W. An inhibition enzyme immunoassay for estimating relative antibody affinity and affinity heterogeneity. *J. Immunol. Methods* **106**, 245–249 (1988). 3276795 10.1016/0022-1759(88)90204-9

