# Supplementary material for: Immune surveillance on the insect body surface recognizes a pathogen-derived fungal protease to activate defenses
Source: Nat Commun. 2026 May 9;17:6268. doi: 10.1038/s41467-026-72836-4 (PMC13376424; doi:10.1038/s41467-026-72836-4)

Supplementary Information

Immune surveillance on the insect body surface recognizes a pathogen-derived fungal protease to activate defenses

Jun Li, Qi Xiao, Petros Ligoxygakis and Yuxian Xia

This file includes:

Supplementary figures 1-13

Supplementary tables 1-3

Uncropped gels of Supplementary figures 2, 3 and 7

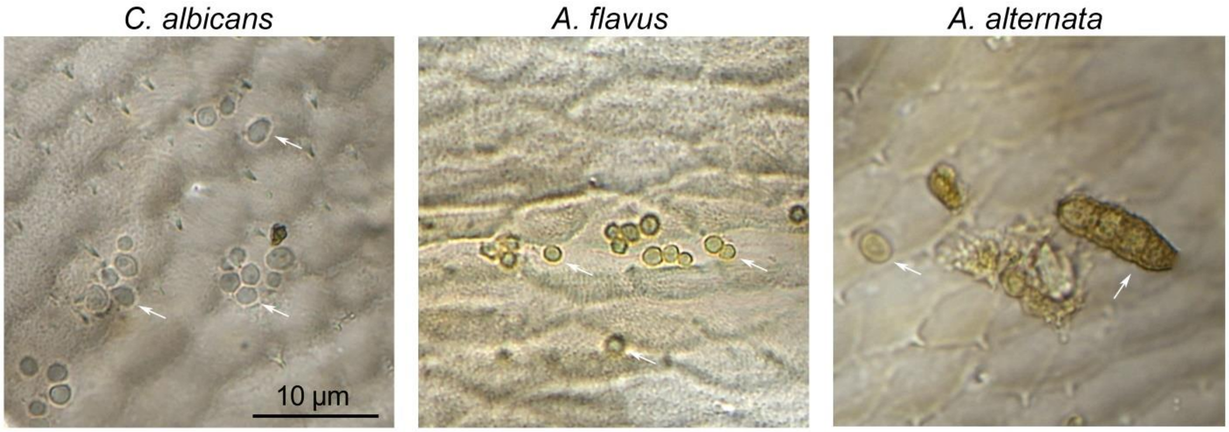

Supplementary figure 1. Non-entomopathogenic fungi spores fail to germinate on body surface of locust

Spores from three non-entomopathogenic fungi were collected from 1/4 SDAY medium and suspended in 0.05% Tween-80 solution to inoculate the cuticular surface of locusts' abdomen. After 72 hours, the local cuticle was dissected, and the epithelial tissue removed. The cuticle was spread on a glass slide for conidial germination observation and photography. Micrographs are representative of three independent experiments with similar results. The scale bar represents 10 μm.

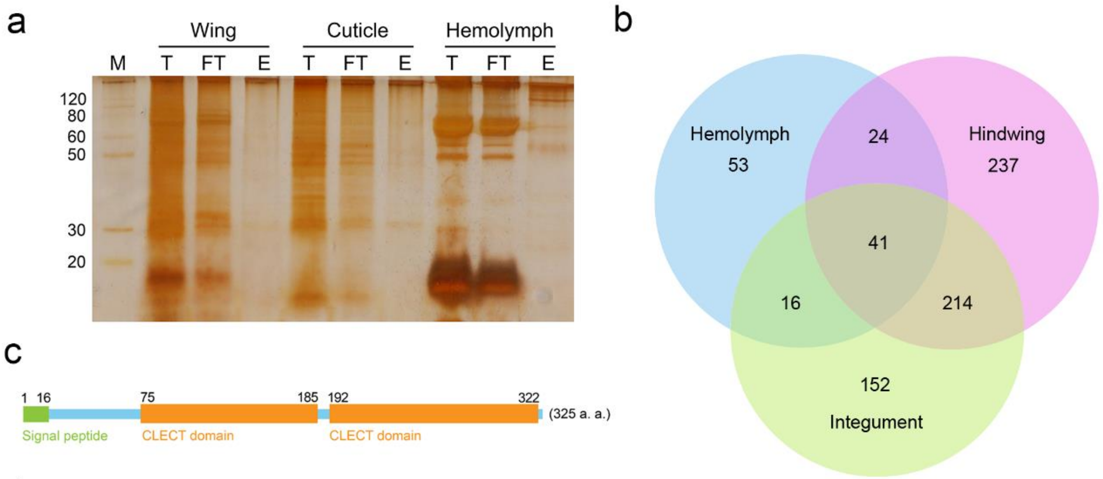

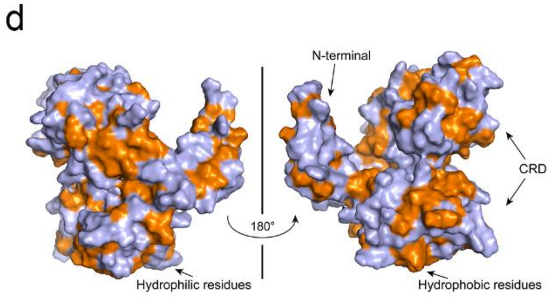

e

Ligand	Affinity (kcal/mol)	Confidence Score
Mannan	-12.53	0.8055
Glucan	-10.80	0.7361
Chitin	-6.13	0.5471

Supplementary figure 2. Identification of locust proteins binding to *M. acridum* conidia

(a) Silver stained 12% SDS-PAGE showing the different steps of *M. acridum* conidia-binding protein isolation by pull-down experiment. Lane 1, 4 and 7: total proteins isolated from hindwing, integument, and cell-free hemolymph, respectively. Lane 2, 5 and 8: flow through from hindwing, integument, and cell-free hemolymph, respectively. Lane 3, 6 and 9: elution fractions from hindwing, integument, and cell-free hemolymph, respectively. (b) Venn diagram revealing *M. acridum* conidia-binding proteins expressed in hindwing, integument, and cell-free hemolymph. (c) Schematic representation showing the carbohydrate recognition domains (CRDs) and signal peptide of the 325-amino acid (a. a.) IML1 protein. (d) AlphaFold-predicted structure of IML1 highlighting surface physicochemical properties. Hydrophobic residues (orange) cluster on one face of the N-terminal region and within the core of both CRDs, whereas the opposite surface is predominantly hydrophilic (blue). (e) The docking affinity and confidence scores between IML1 and mannan, glucan, or chitin were predicted by AutoDock Vina. Source data are provided as a Source Data file.

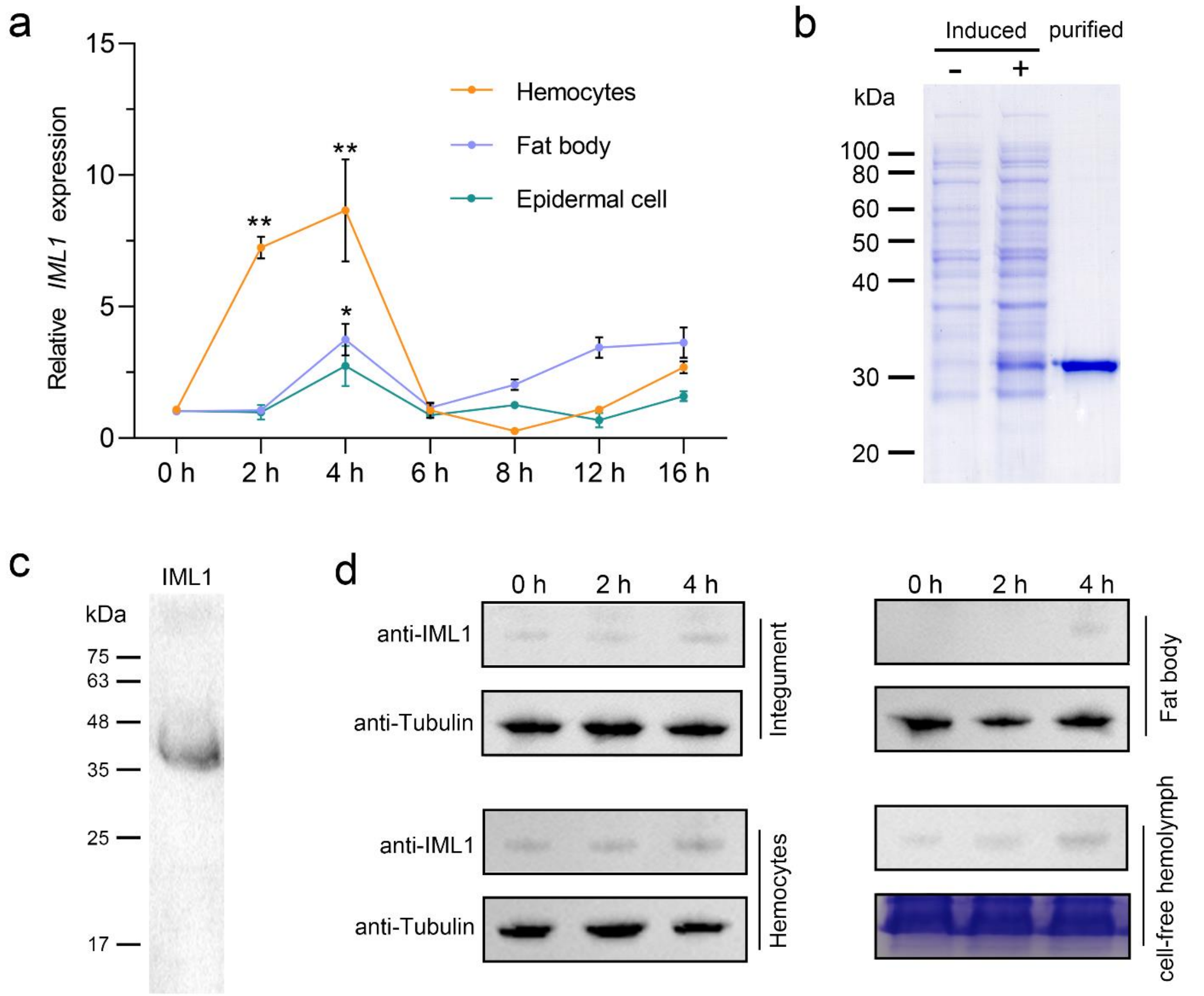

Supplementary figure 3. Recombinant protein purification, antibody validation, and expression dynamics

(a) qRT-PCR results showing *IML1* transcription levels in locust epidermal cells, fat body, and hemocytes at 0-, 2-, and 4-hours post-inoculation with *M. acridum* conidia. (b) Representative SDS-PAGE gel stained with Coomassie blue assessing the production and purification of recombinant IML1 (rIML1). (c) Assessment of anti-IML1 antibody specificity on hemocytes protein extract by western blot. (d) Western blot images showing IML protein levels in locust integument, cell-free hemolymph, hemocytes, and fat body at 0-, 2-, and 4-hours post-inoculation with *M. acridum* conidia. Asterisks indicate significant differences analyzed by ANOVA (Tukey's HSD test, $*p < 0.05$; $**p < 0.01$). Data represent mean ± standard error of the mean (SEM) of three biological replicates. Source data are provided as a Source Data file.

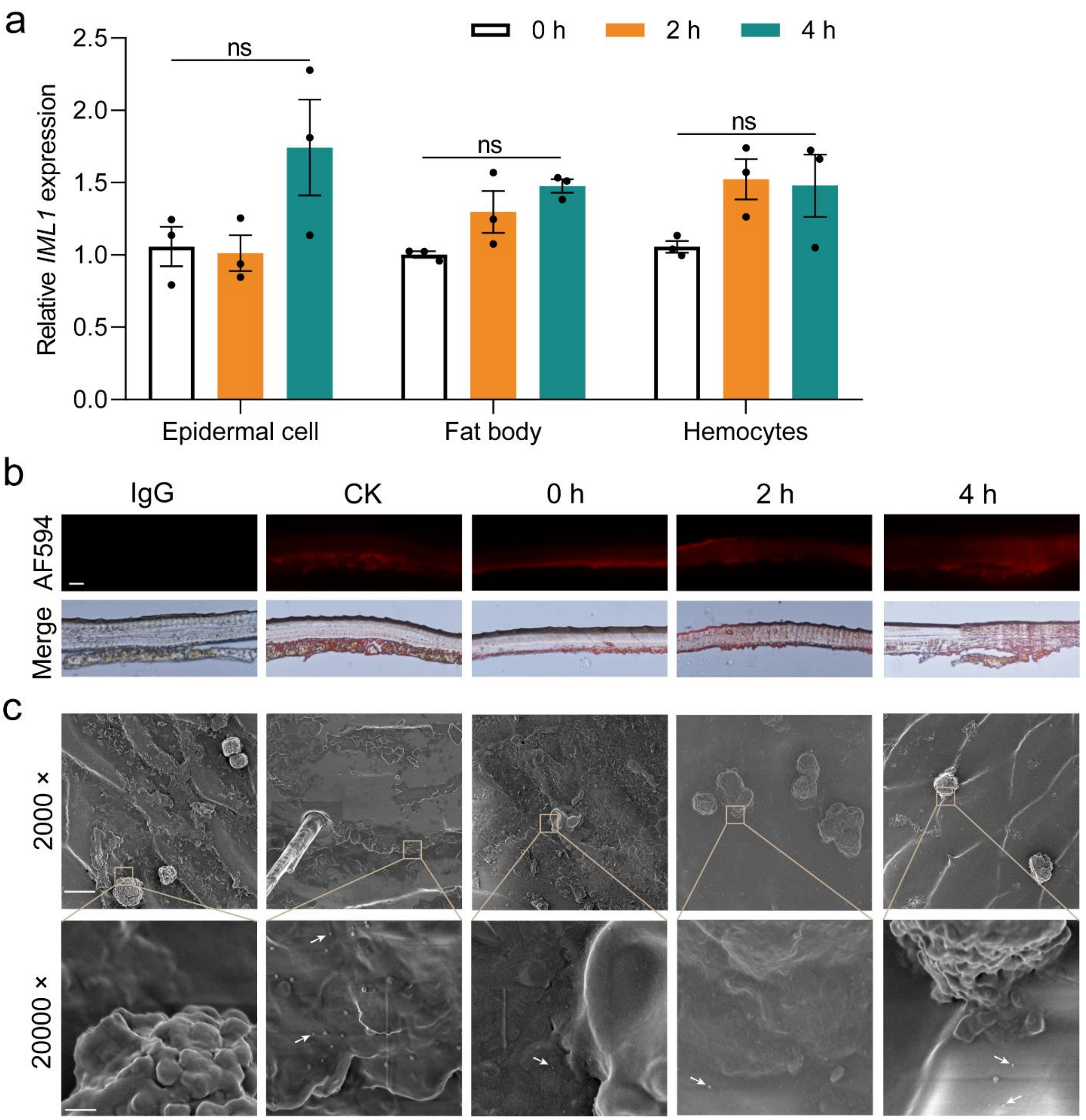
a
0 h
2 h
4 h
Relative IML1 expression
2.5
2.0
1.5
1.0
0.5
0.0
ns
ns
ns
Epidermal cell
Fat body
Hemocytes
b
IgG
CK
0 h
2 h
4 h
AF594
Merge
c
2000 ×
20000 ×

Supplementary figure 4. The non-pathogenic fungus *Aspergillus flavus* fails to induce a *IML1* expression and mobilization despite IML1 binding

(a) qRT-PCR analysis of *IML1* transcription in epidermal cells, fat body, and hemocytes at 0, 2, and 4 hours after topical inoculation with *A. flavus* conidia. Data are shown as mean ± SEM three biological replicates. ns, not significant. (b) Immunofluorescence analysis showing IML1 protein distribution in cuticle sections at 0-, 2-, and 4-hours post-inoculation with *A. flavus* conidia. Red fluorescence (Alexa Fluor 594, AF594) indicates IML1 protein detected by anti-IML1 antibody. For negative control, normal IgG was used as the primary antibody. Scale bar represents 10 μm. (c) Scanning electron microscopy analysis confirming the binding of IML1 to *A. flavus* conidia on the locust body surface. White arrows indicate 35-nm colloidal gold particles conjugated to a secondary antibody bound to the anti-IML1 primary antibody. A parallel control using normal IgG as the primary antibody. The upper panel (2,000× magnification; scale bar, 10 μm) provides an overview, while the lower panel (20,000× magnification; scale bar, 1 μm) shows a detailed view. Micrographs in (b,c) are representative of three independent experiments with similar results. Source data are provided as a Source Data file.

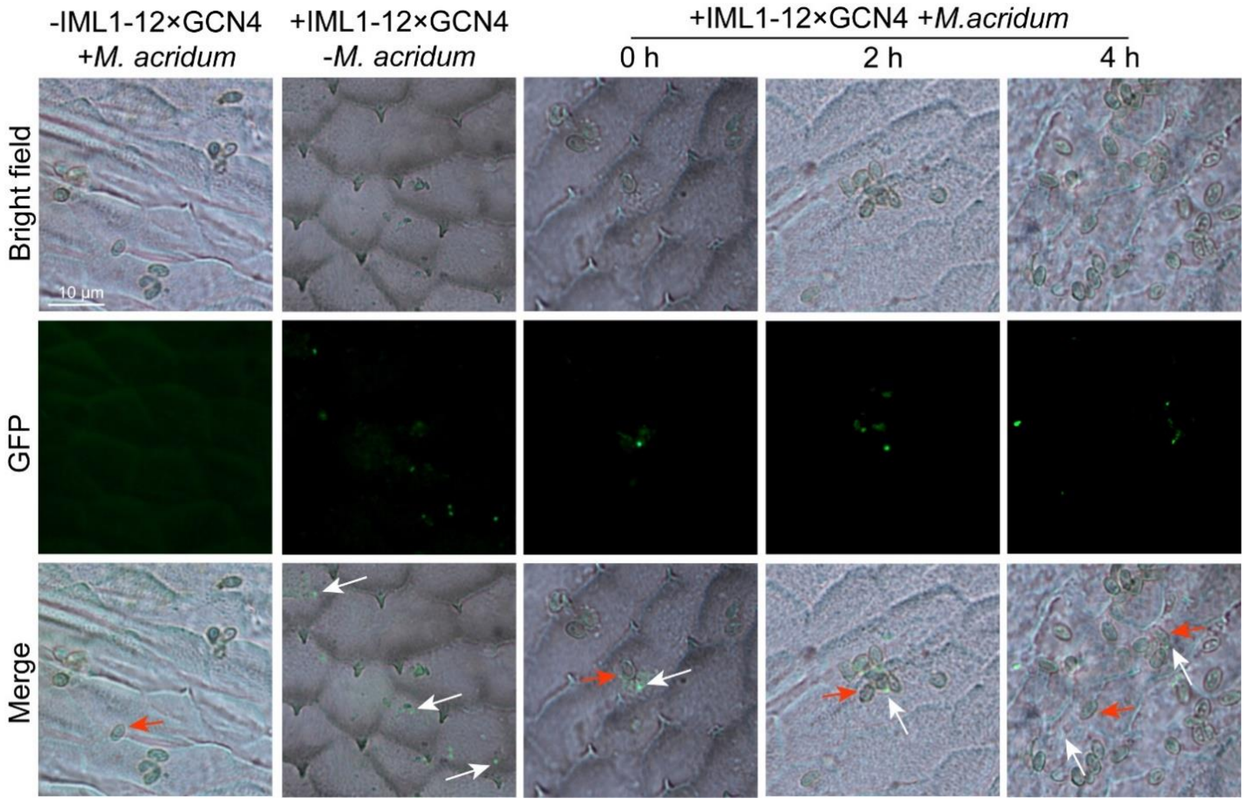

Supplementary figure 5. SunTag system validates IML1 secretion from hemolymph to fungal spores on body surface

Recombinant IML1 fused with 12×GCN4 peptide array was injected into the locust hemocoel. Twelve hours post-injection, *M. acridum* conidia were topically applied to the abdominal cuticle. At 0-, 2-, and 4-hours post-inoculation, the cuticle from the inoculation site was dissected (with basal membrane and epidermal cells removed) and stained with single-chain variable fragment fused to GFP (ScFv-GFP), which specifically binds to the GCN4 peptide epitopes. Fluorescence microscopy revealed progressive accumulation of IML1-12×GCN4 around attached conidia over time, confirming IML1 secretion from hemolymph to the body surface. The controls included injection of IML1-12×GCN4 without conidia inoculation, and inoculation of *M. acridum* conidia without IML1-12×GCN4 injection. In the labels, + and − indicate the presence and absence of IML1-12×GCN4 injection or *M. acridum* inoculation, respectively. Red arrows indicate fungal conidia; white arrows indicate GFP fluorescent signals representing IML1-12×GCN4 protein. Micrographs are representative of three independent experiments with similar results. Scale bar, 10 μm.

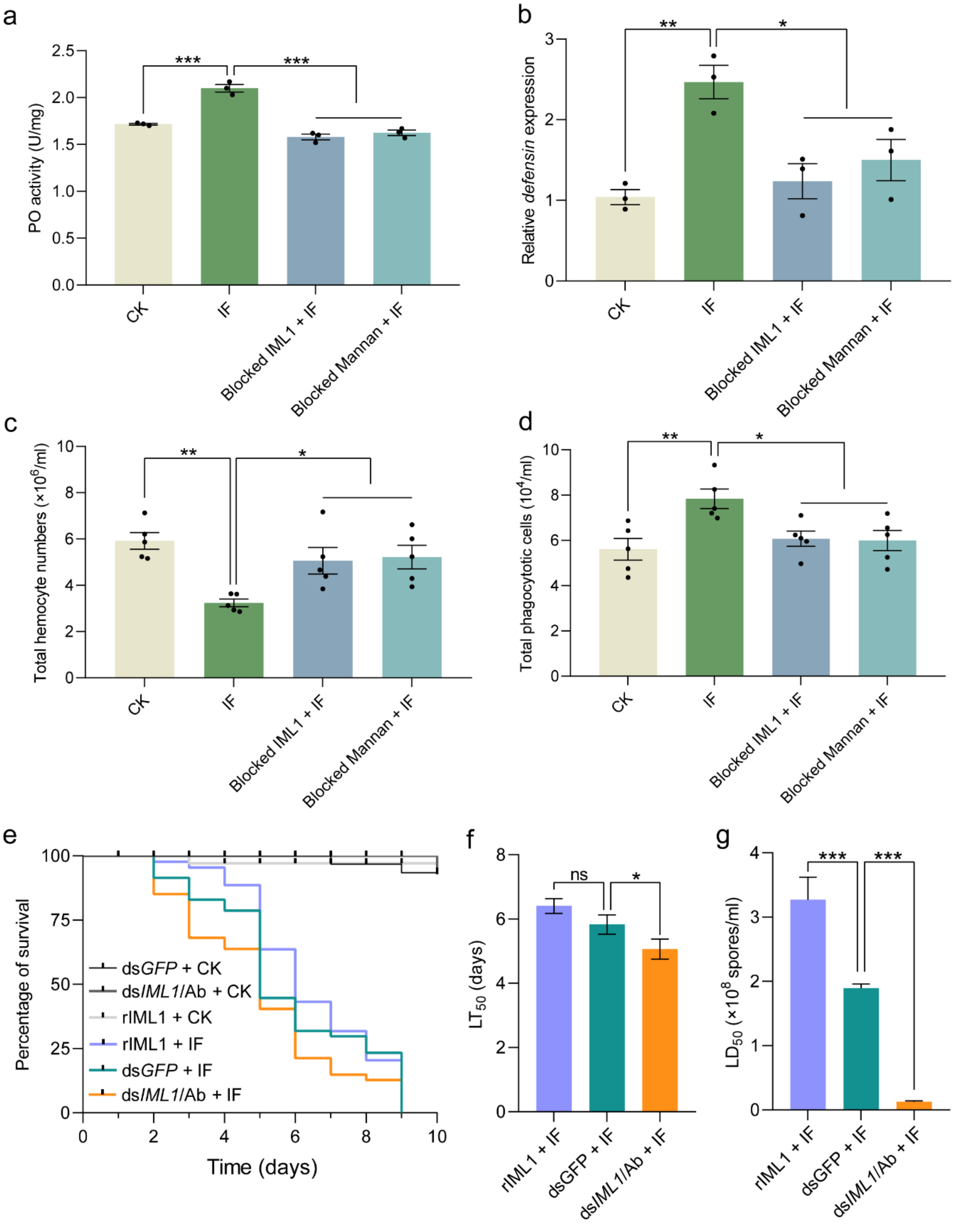
a
PO activity (U/mg)
0.0
0.5
1.0
1.5
2.0
2.5
***
***
CK
IF
Blocked IML1 + IF
Blocked Mannan + IF
b
Relative defensin expression
0
1
2
3
**
*
CK
IF
Blocked IML1 + IF
Blocked Mannan + IF
c
Total hemocyte numbers (×10^6/ml)
0
2
4
6
8
10
**
*
CK
IF
Blocked IML1 + IF
Blocked Mannan + IF
d
Total phagocytotic cells (10^4/ml)
0
2
4
6
8
10
**
*
CK
IF
Blocked IML1 + IF
Blocked Mannan + IF
e
Percentage of survival
0
25
50
75
100
dsGFP + CK
dsIML1/Ab + CK
rIML1 + CK
rIML1 + IF
dsGFP + IF
dsIML1/Ab + IF
0
2
4
6
8
10
Time (days)
f
LT50 (days)
0
2
4
6
8
ns
*
rIML1 + IF
dsGFP + IF
dsIML1/Ab + IF
g
LD50 (×10^8 spores/ml)
0
1
2
3
4
***
***
rIML1 + IF
dsGFP + IF
dsIML1/Ab + IF

Supplementary figure 6. Blocking and disrupting IML1-mediated surveillance potentiates fungal virulence

(a-d) Blocking either the fungal ligand or the host receptor suppresses the initial immune response. Locusts were treated with an anti-IML1 antibody (blocked IML1) or a mannan-binding lectin (MBL, blocked Mannan) before inoculation with *M. acridum*. Immune parameters were measured 4 hours later: (a) cuticular PO activity, (b) *defensin* expression, (c) total hemocytes, and (d) phagocytic cells. (e-g) Manipulating host IML1 levels directly impacts survival. We suppressed IML1 using a dsIML1/Ab cocktail or supplemented it with recombinant protein (rIML1) before fungal challenge. (e) IML1 suppression accelerated mortality, while supplementation was protective. (f) Median lethal time (LT_{50}) comparison. (g) Median lethal dose (LD_{50}) was significantly lower in IML1-suppressed locusts compared to dsGFP controls. CK, non-infected check control; IF, infected with *M. acridum*; ds*GFP*, dsRNA targeting green fluorescent protein (negative control); ds*IML1*, dsRNA targeting IML1; ds*IML1*/Ab, ds*IML1* plus anti-IML1 antibody; rIML1, recombinant IML1 protein. Data are shown as mean ± SEM (*n* = 3 replicates). Statistical significance was assessed by log-rank (Mantel-Cox) test for survival curves (e) and by two-sided Student's t-test for for LD_{50} values (f) (**p* < 0.05, ****p* < 0.001, ns, not significant). Source data are provided as a Source Data file.

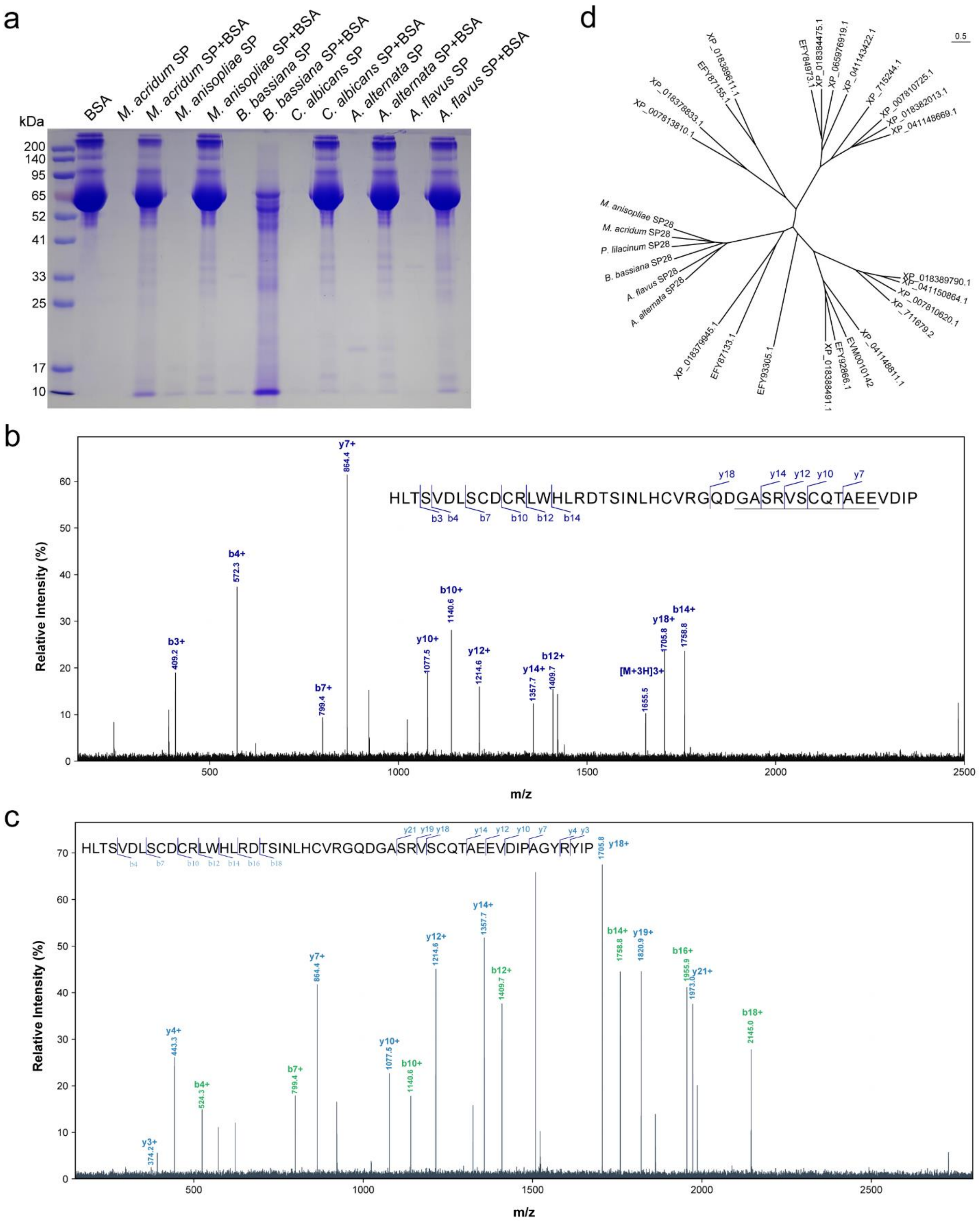
a
kDa
200
140
95
65
52
41
33
25
17
10
BSA
M. acridum SP
M. acridum SP+BSA
M. anisopliae SP
M. anisopliae SP+BSA
B. bassiana SP
B. bassiana SP+BSA
C. albicans SP
C. albicans SP+BSA
A. alternata SP
A. alternata SP+BSA
A. flavus SP
A. flavus SP+BSA
d
0.5
M. anisopliae SP28
M. acridum SP28
P. lilacinum SP28
B. bassiana SP28
A. flavus SP28
A. alternata SP28
XP_018379945.1
EFY87133.1
EFY93305.1
XP_018388491.1
EFY92866.1
EVM0010142
XP_041148811.1
XP_711679.2
XP_007810620.1
XP_041150864.1
XP_018389790.1
XP_007813810.1
XP_018378833.1
EFY87155.1
XP_018389611.1
EFY84973.1
XP_018384475.1
XP_065976919.1
XP_041143422.1
XP_715244.1
XP_007810725.1
XP_018382013.1
XP_041148669.1
b
HLTSVDLSCDCRLWHLRDTSINLHCVRGQDGASRVSCQTAEEVDIP
Relative Intensity (%)
m/z
b3+ 409.2
b4+ 572.3
b7+ 799.4
y7+ 864.4
y10+ 1077.5
b10+ 1140.6
y12+ 1214.6
y14+ 1357.7
b12+ 1409.7
[M+3H]3+ 1655.5
y18+ 1705.8
b14+ 1758.8
c
HLTSVDLSCDCRLWHLRDTSINLHCVRGQDGASRVSCQTAEEVDIPAGYRYIP
y3+ 374.2
y4+ 443.3
b4+ 524.3
b7+ 799.4
y7+ 864.4
y10+ 1077.5
b10+ 1140.6
y12+ 1214.6
y14+ 1357.7
b12+ 1409.7
y18+ 1705.8
b14+ 1758.8
y19+ 1820.9
b16+ 1955.9
y21+ 1973.0
b18+ 2145.0

Supplementary figure 7. Protease activity of fungal conidial extracts, IML1 cleavage products, and phylogenetic analysis of serine proteases

(a) SDS-PAGE analysis showing proteolytic digestion of bovine serum albumin (BSA) by surface protein extracts from entomopathogenic (*M. acridum*, *M. anisopliae*, *B. bassiana*) and non-entomopathogenic fungi (*C. albicans*, *A. alternata*, *A. flavus*). All fungal extracts exhibit proteolytic activity on BSA. (b) Mass spectrometry (MS/MS) identification of the small bioactive peptide IML1ac generated by MacSP28 cleavage of IML1. (c) MS/MS identification of the small bioactive peptide IML1an generated by ManSP28 cleavage of IML1. (d) Phylogenetic analysis of serine proteases from entomopathogenic and non-entomopathogenic fungi. Serine proteases were identified in *M. acridum* and *M. anisopliae* by LC-MS/MS analysis of conidial surface proteins, and predicted from *C. albicans*, *A. flavus*, and *A. alternata* genome sequences. Protein alignment and phylogenetic tree were generated using MEGA 12.0 by the Neighbor-Joining method. Red branches highlight SP28 proteases characteristic of *M. acridum* and *M. anisopliae*, which form a distinct clade separate from non-entomopathogenic fungal proteases. Scale bar for phylogenetic tree, 0.2 substitutions per site. Source data are provided as a Source Data file.

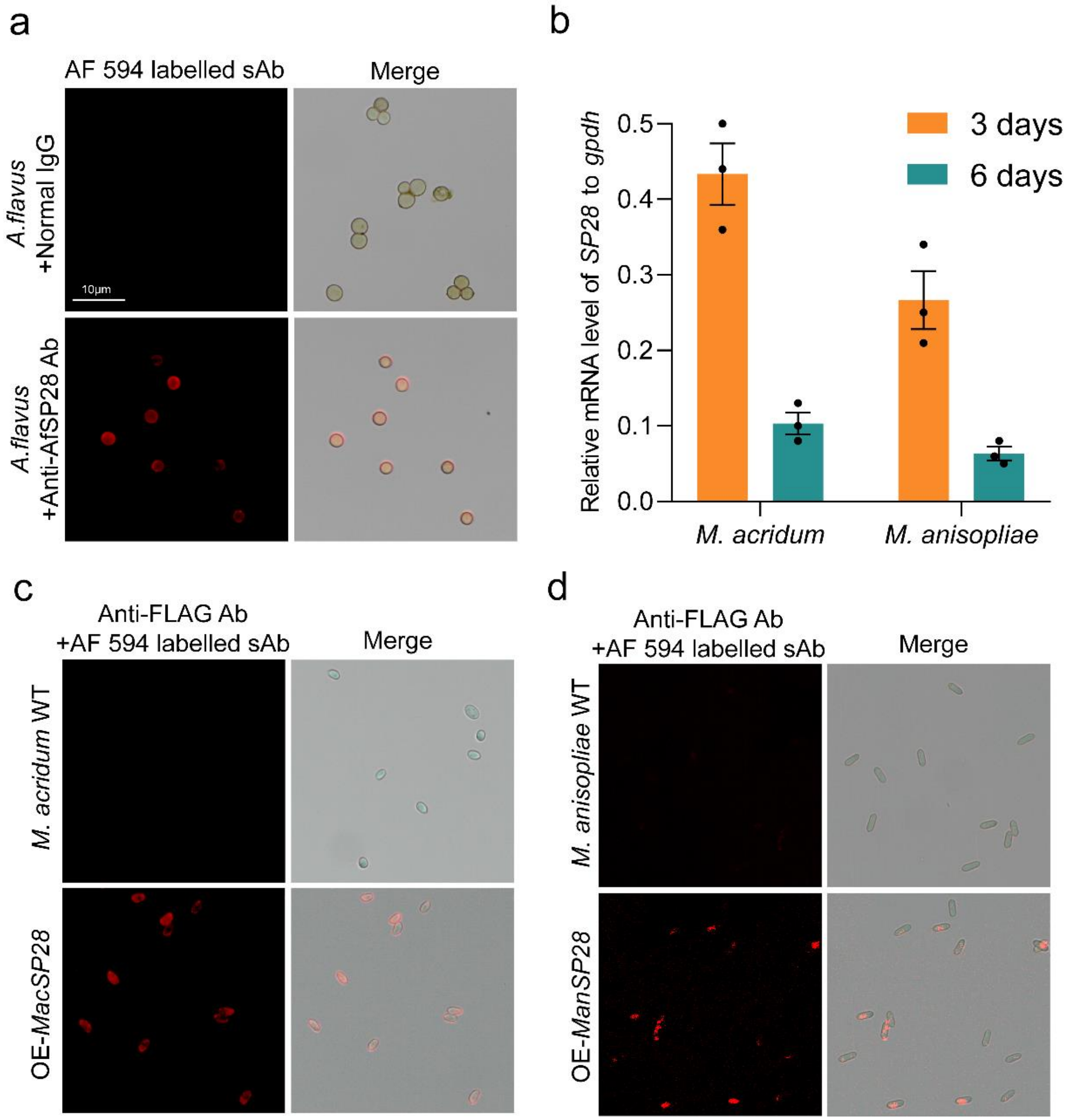

a
AF 594 labelled sAb
Merge
A.flavus +Normal IgG
10μm
A.flavus +Anti-AfSP28 Ab
b
Relative mRNA level of SP28 to gpdh
0.5
0.4
0.3
0.2
0.1
0.0
3 days
6 days
M. acridum
M. anisopliae
c
Anti-FLAG Ab +AF 594 labelled sAb
Merge
M. acridum WT
OE-MacSP28
d
Anti-FLAG Ab +AF 594 labelled sAb
Merge
M. anisopliae WT
OE-ManSP28

Supplementary figure 8. Surface localization of SP28 in entomopathogenic and non-entomopathogenic fungi

(a) Immunofluorescence detection of endogenous AfSP28 on *A. flavus* conidial surface. Conidia were stained with anti-AfSP28 polyclonal antibody or normal IgG (negative control), followed by Alexa Fluor 594-conjugated secondary antibody (sAb). (b) Relative mRNA expression levels of *SP28* in conidia of *M. acridum*, and *M. anisopliae* wild type (WT) harvested at 3 days and 6 days cultured on 1/4 SDAY medium. Data are shown as mean ± SEM (n = 3 replicates). (c) Immunofluorescence localization of FLAG-tagged MacSP28 on *M. acridum* conidial surface. Wild-type (WT) and overexpression (OE-*MacSP28*) strains were stained with anti-FLAG antibody followed by Alexa Fluor 594-conjugated secondary antibody. (d) Immunofluorescence localization of FLAG-tagged ManSP28 on *M. anisopliae* conidial surface. WT and overexpression (OE-*ManSP28*) strains were stained with anti-FLAG antibody followed by Alexa Fluor 594-conjugated secondary antibody. Micrographs are representative of three independent experiments with similar results. Scale bar, 10 μm. Source data are provided as a Source Data file.

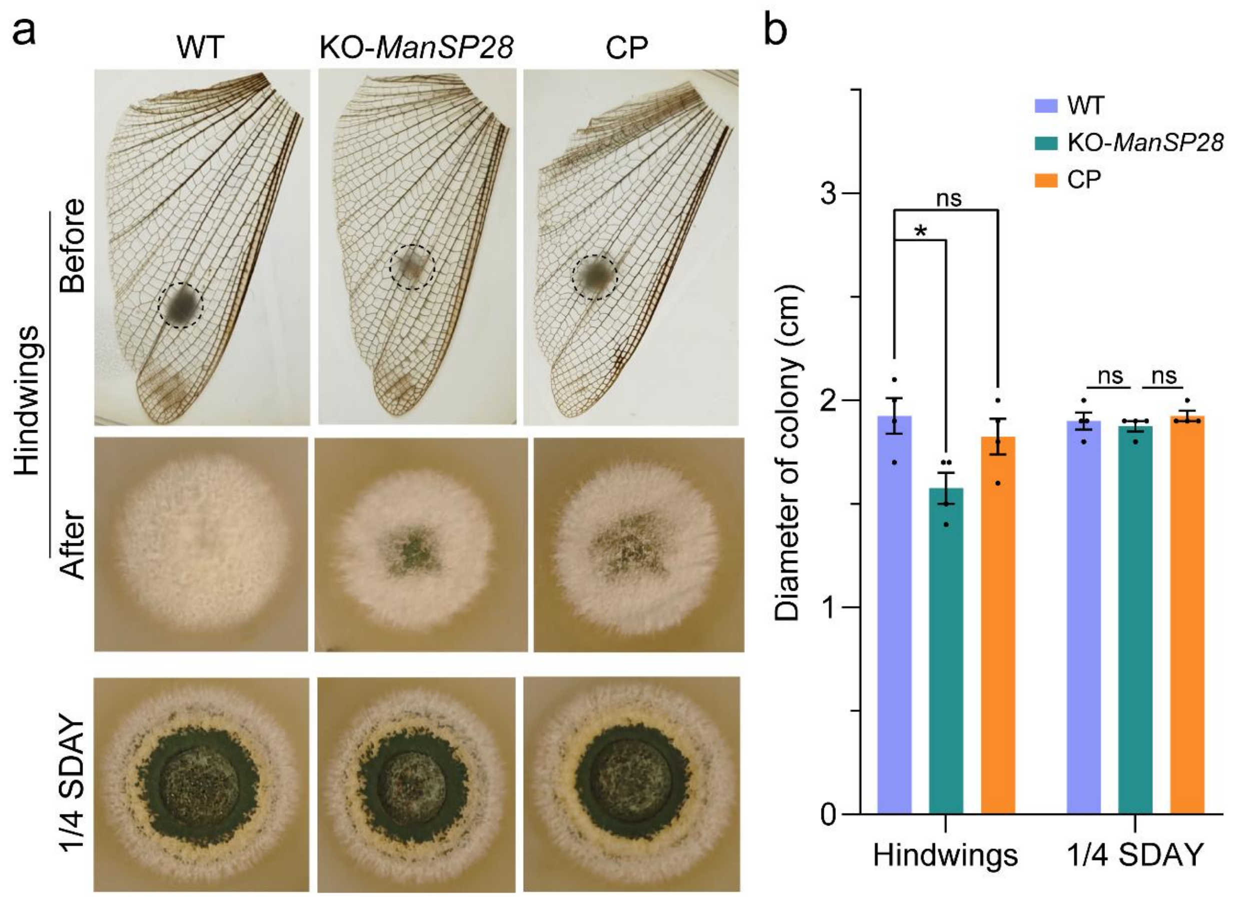

Supplementary figure 9. Deletion of SP28 impairs *M. anisopliae* penetration ability on locust cuticle but not growth on artificial medium

(a) Representative images showing *M. anisopliae* wild-type (WT), knockout mutant (KO-*ManSP28*), and complemented strain (CP) growth assays. Top panels (Before): Hindwings were placed on 1/4 SDAY medium and inoculated with conidial suspensions; images were taken after 3 days. Middle panels (After): After removing hindwings, plates were cultured for an additional 3 days to allow penetrated fungi to grow out on the medium. Bottom panels (1/4 SDAY): Conidial suspensions were directly inoculated onto 1/4 SDAY medium and cultured for 3 days. (b) Quantification of colony diameter. Before represents growth on hindwings; After represents colony diameter after cuticle penetration; 1/4 SDAY represents direct growth on medium. Data are shown as mean ± SEM (n = 3 replicates). Asterisk indicates significant difference analyzed by ANOVA (Tukey's HSD test) (*$p < 0.05$; ns, not significant). All experiments were repeated four times with similar results. Source data are provided as a Source Data file.

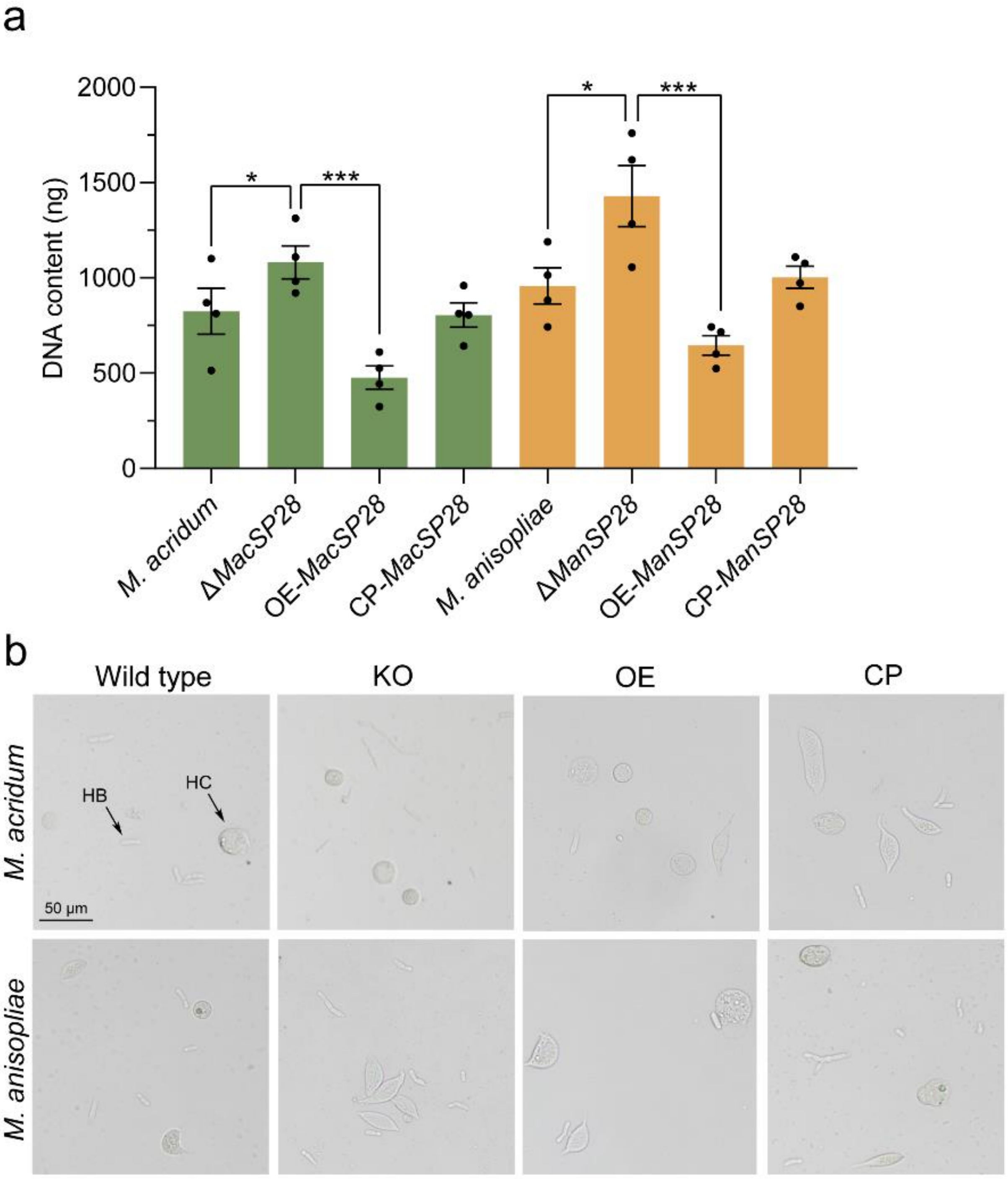
a
DNA content (ng)
2000
1500
1000
500
0
*
***
*
***
M. acridum
ΔMacSP28
OE-MacSP28
CP-MacSP28
M. anisopliae
ΔManSP28
OE-ManSP28
CP-ManSP28
b
Wild type
KO
OE
CP
M. acridum
M. anisopliae
HB
HC
50 μm

Supplementary figure 10. Effect of *SP28* mutations on fungal invasiveness after inoculation with *M. acridum* or *M. anisopliae*

(a) Wild type, knockout (KO), expression (OE), or complementray (CP) forms of *SP28* in *M*. *acridum* and *M. anisopliae* were inoculated to locusts. After three days, fungal DNA load in hemolymph was quantified by RT-qPCR. Data represent mean ± SEM of three biological replicates. Asterisks indicate significant differences analyzed by two-tailed t test (*$p < 0.05$, ***$p < 0.001$). (b) Microscope images of hemolymph collected after fungi inoculation. HB, yeast-like hyphal bodies; HC, hemocytes. Scale bar, 50 μm. Data represent mean ± SEM of four biologically independent samples. Micrographs are representative of three independent experiments with similar results. Source data are provided as a Source Data file.

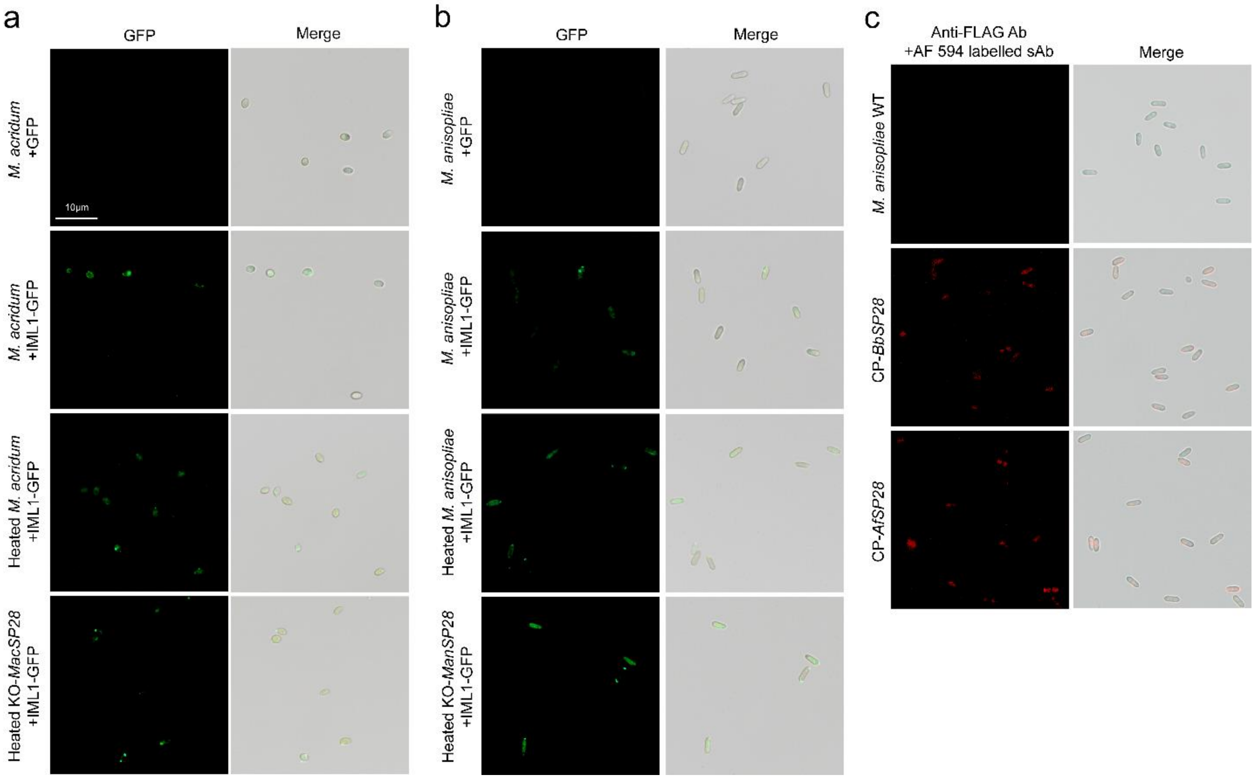

Supplementary figure 11. Heterologous SP28 localization and IML1-binding capacity of fungal conidia

(a, b) IML1-binding capacity of fungal conidia before and after heat inactivation. *M. acridum* (a) and *M. anisopliae* (b) wild-type or knockout (KO) of *SP28* mutant conidia were either untreated or heat-killed (100°C for 20 min), then incubated with GFP-tagged IML1 (IML1-GFP) or GFP alone (negative control). Binding was visualized by fluorescence microscopy. (c) Immunofluorescence localization of heterologous FLAG-tagged SP28 on *M. anisopliae* conidial surface. Knock-in strains expressing *A. flavus SP28* (KI-*AfSP28*) or *B. bassiana SP28* (KI-*BbSP28*) were stained with anti-FLAG antibody followed by Alexa Fluor 594-conjugated secondary antibody. Wild-type (WT) serves as negative control. Micrographs are representative of three independent experiments with similar results. Scale bar, 10 μm.

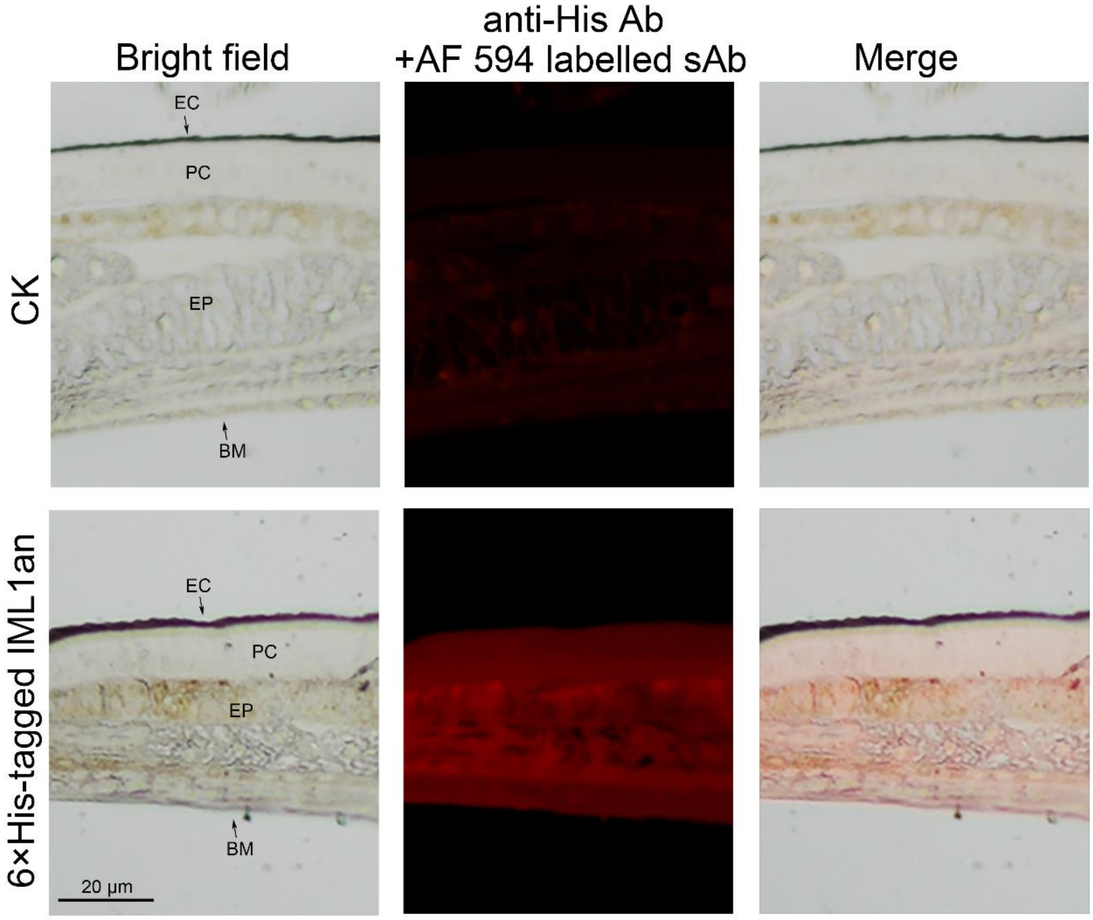

Supplementary figure 12. Location of IML1an on the locust cuticle

Recombinant IML1an peptide carrying a 6×His tag was inoculated on locusts' abdomen cuticles. After three hours, the cuticle was dissected, fixed in glutaric dialdehyde, and embedded into paraffin for sectioning. The sections were incubated with an anti-His antibody, stained by Alexa Fluor 594-conjugated secondary antibody (sAb). The sections were imaged by fluorescence microscope. Micrographs are representative of three independent experiments with similar results. Scale bar, 20μm EC, epicuticle; PC, procuticle; EP, epidermal cells; BM, basal membrane.

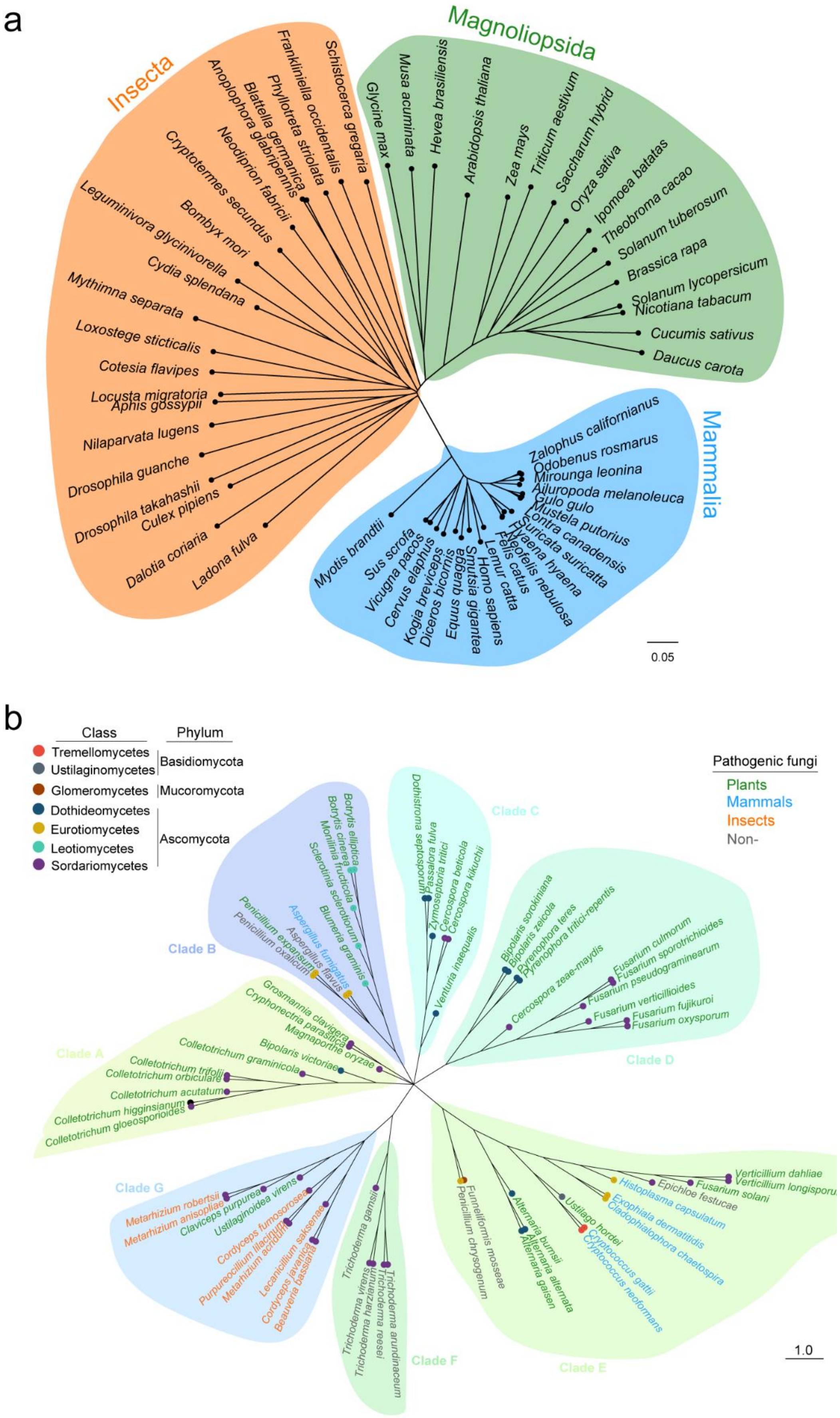

a
Insecta
Magnoliopsida
Mammalia
Schistocerca gregaria
Frankliniella occidentalis
Phyllotreta striolata
Blattella germanica
Anoplophora glabripennis
Neodiprion fabricii
Cryptotermes secundus
Bombyx mori
Leguminivora glycinivorella
Cydia splendana
Mythimna separata
Loxostege sticticalis
Cotesia flavipes
Locusta migratoria
Aphis gossypii
Nilaparvata lugens
Drosophila guanche
Drosophila takahashii
Culex pipiens
Dalotia coriaria
Ladona fulva
Glycine max
Musa acuminata
Hevea brasiliensis
Arabidopsis thaliana
Zea mays
Triticum aestivum
Saccharum hybrid
Oryza sativa
Ipomoea batatas
Theobroma cacao
Solanum tuberosum
Brassica rapa
Solanum lycopersicum
Nicotiana tabacum
Cucumis sativus
Daucus carota
Zalophus californianus
Odobenus rosmarus
Mirounga leonina
Ailuropoda melanoleuca
Gulo gulo
Mustela putorius
Lontra canadensis
Suricata suricatta
Hyaena hyaena
Neofelis nebulosa
Felis catus
Lemur catta
Homo sapiens
Smutsia gigantea
Equus quagga
Diceros bicornis
Kogia breviceps
Cervus elaphus
Vicugna pacos
Sus scrofa
Myotis brandtii
0.05
b
Class
Phylum
Tremellomycetes
Ustilaginomycetes
Basidiomycota
Glomeromycetes
Mucoromycota
Dothideomycetes
Eurotiomycetes
Leotiomycetes
Sordariomycetes
Ascomycota
Pathogenic fungi
Plants
Mammals
Insects
Non-
Clade A
Clade B
Clade C
Clade D
Clade E
Clade F
Clade G
Botrytis elliptica
Botrytis cinerea
Monilinia fructicola
Sclerotinia sclerotiorum
Blumeria graminis
Aspergillus fumigatus
Aspergillus flavus
Penicillium expansum
Penicillium oxalicum
Dothistroma septosporum
Passalora fulva
Zymoseptoria tritici
Cercospora beticola
Cercospora kikuchii
Venturia inaequalis
Bipolaris sorokiniana
Bipolaris zeicola
Pyrenophora teres
Pyrenophora tritici-repentis
Cercospora zeae-maydis
Fusarium culmorum
Fusarium sporotrichioides
Fusarium pseudograminearum
Fusarium verticillioides
Fusarium fujikuroi
Fusarium oxysporum
Grosmannia clavigera
Cryphonectria parasitica
Magnaporthe oryzae
Bipolaris victoriae
Colletotrichum graminicola
Colletotrichum trifolii
Colletotrichum orbiculare
Colletotrichum acutatum
Colletotrichum higginsianum
Colletotrichum gloeosporioides
Metarhizium robertsii
Metarhizium anisopliae
Claviceps purpurea
Ustilaginoidea virens
Cordyceps fumosorosea
Purpureocillium lilacinum
Metarhizium acridum
Lecanicillium saksenae
Cordyceps javanica
Beauveria bassiana
Trichoderma gamsii
Trichoderma virens
Trichoderma harzianum
Trichoderma reesei
Trichoderma arundinaceum
Funneliformis mosseae
Penicillium chrysogenum
Alternaria burnsii
Alternaria alternata
Alternaria gaisen
Ustilago hordei
Cryptococcus gattii
Cryptococcus neoformans
Histoplasma capsulatum
Exophiala dermatitidis
Cladophialophora chaetospira
Epichloe festucae
Fusarium solani
Verticillium dahliae
Verticillium longisporum
1.0

Supplementary figure 13. Phylogenetic analysis of IML1-like lectins and SP28 proteases

(a) Phylogenetic analysis by Neighbor-Joining method using MEGA 12.0 shows that the host target, IML1, is part of a conserved insect lectin family. (b) The protease SP28 are found in pathogenic and non-pathogenic fungi, there are significantly divergence between pathogenic and non-pathogenic fungi. The mechanism is functionally conserved across different host-pathogen systems.

Supplementary table 1. Four potential receptor proteins. The sequence can be found in locustDB (http://locustdb.genomics.org.cn).

GeneID	GeneName	Unique peptide numbers		
		Cuticle	Wings	Hemocytes
LOCMI14814	Lipoprotein	8	15	72
LOCMI10363	Immulectin-1, IML1	8	9	20
LOCMI08613	GNBP3	6	12	3
LOCMI09790	Obstructor E2	7	4	4

Supplementary table 2. Sequence of two small peptides of IML1 produced *M. acridum* and *M. anisopliae*.

Name	Sequence
IML1ac	HLTSVDLSCDCRLWHLRDTSINLHCVRGQDGASRVSCQTAEEVDIP
IML1an	HLTSVDLSCDCRLWHLRDTSINLHCVRGQDGASRVSCQTAEEVDIPAGYRYIP

Supplementary table 3. The serine proteases identified from *M. acridum*, *M. anisopliae*, *C. albicans*, *A. alternata*, and *A. flavus*. The accession numbers can find NCBI (https://www.ncbi.nlm.nih.gov/).

Species	Accession number
M. acridum	EFY93305.1
M. acridum	EFY92866.1
M. acridum	EFY87133.1
M. acridum	EFY84973.1
M. acridum	XP_007813810.1
M. acridum	XP_007810725.1
M. acridum	EFY87155.1
M. acridum	XP_007810620.1
M. acridum	XP_065976919.1
M. acridum	EFY85443.1 (MacSP28)
M. anisopliae	KAK8918209.1 (ManSP28)
M. anisopliae	KAK8922447.1
A. alternata	XP_018380840.1
A. alternata	XP_018378833.1
A. alternata	XP_018384475.1
A. alternata	XP_018380418.1
A. alternata	XP_018382013.1
A. alternata	XP_018384287.1
A. alternata	XP_018380630.1
A. alternata	XP_018381804.1
A. alternata	XP_018384474.1
A. alternata	XP_018391216.1 (AaSP28)
A. flavus	XP_041143422.1 (AfSP28)
A. flavus	XP_041148669.1
C. albicans	XP_718622.1
C. albicans	XP_715244.1

Source Data for the gel shown in Supplementary figure 2

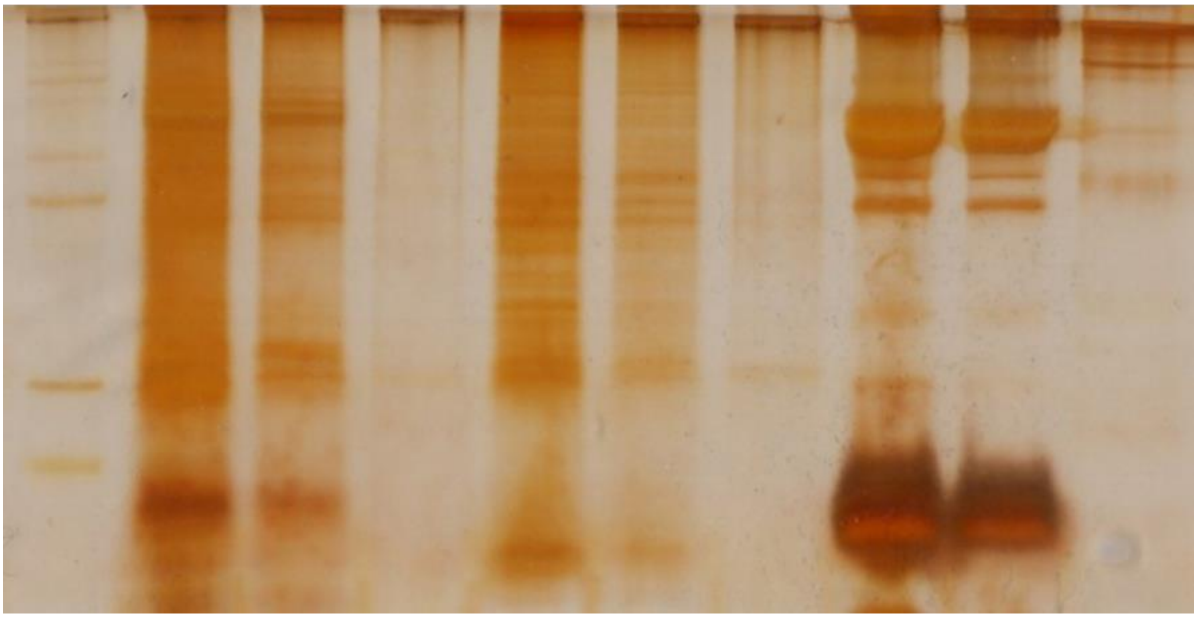

Source Data for the gel shown in Supplementary figure 3

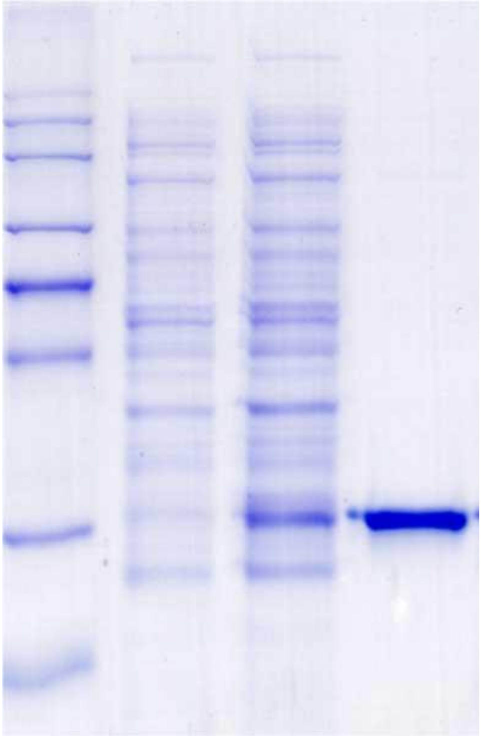

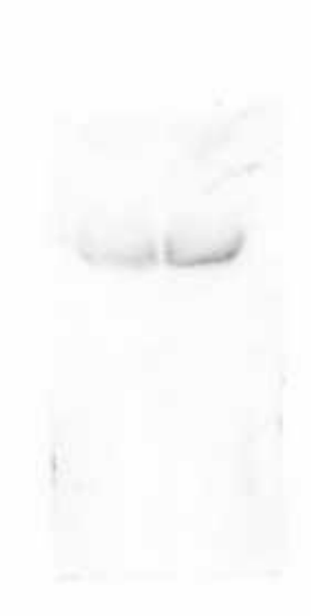

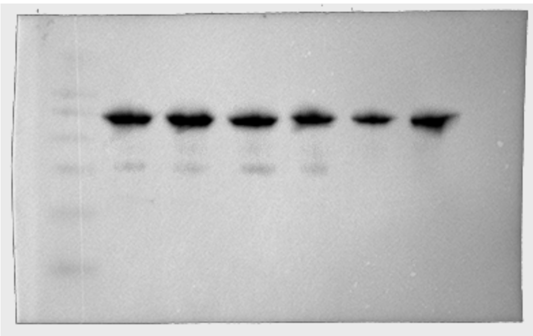

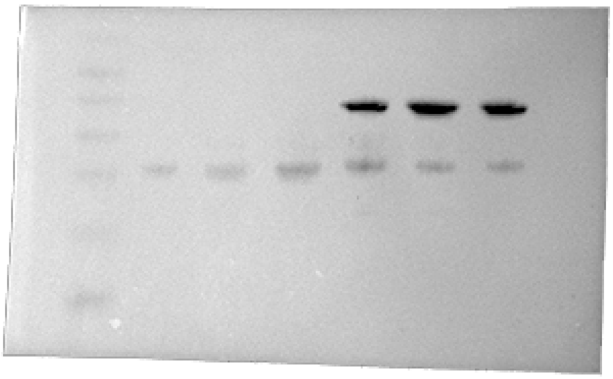

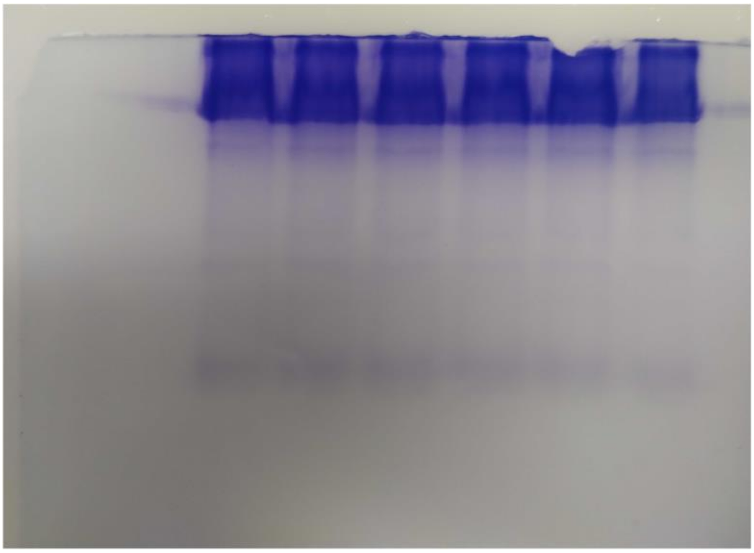

Source Data for the gel shown in Supplementary figure 7

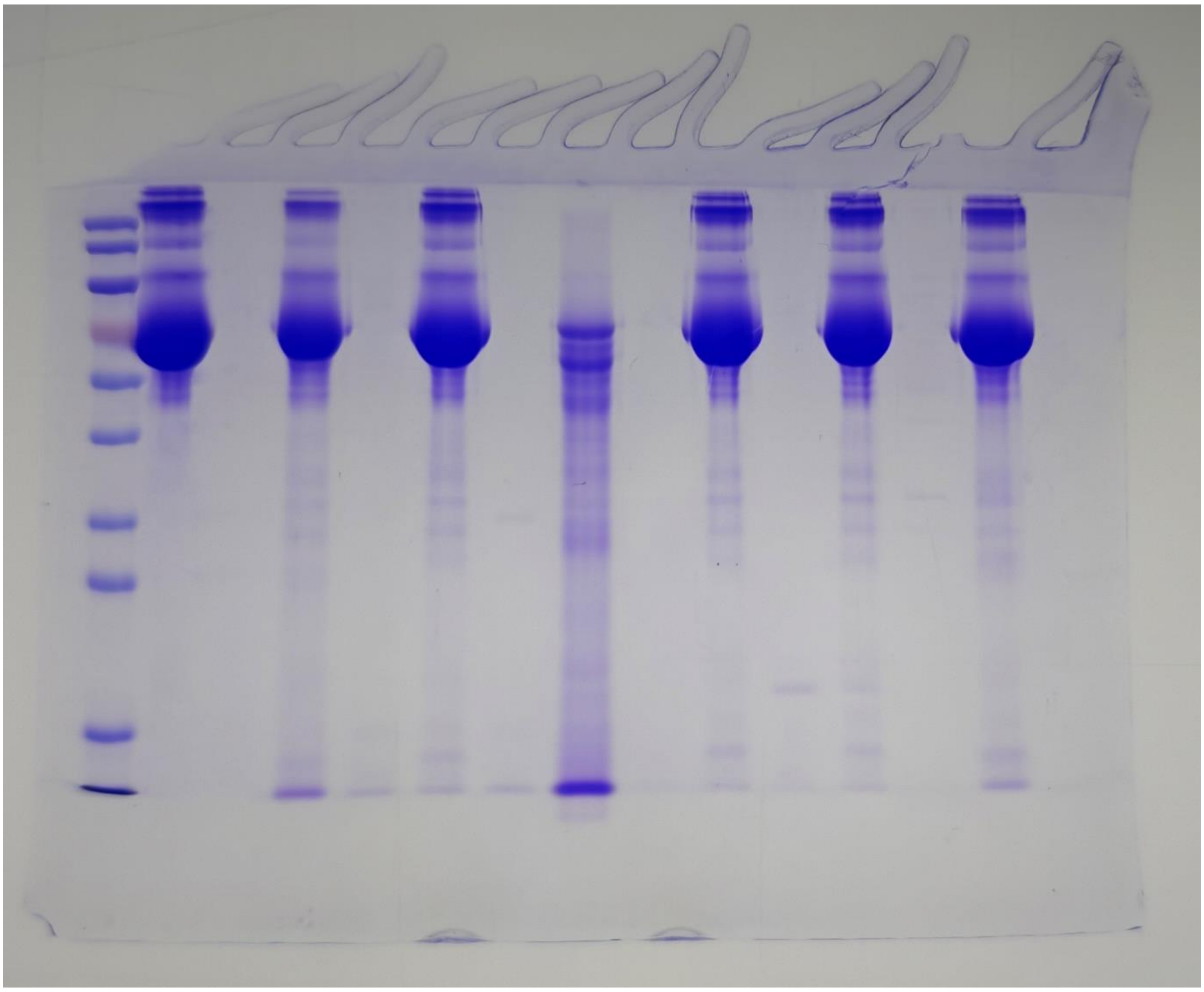

Supplement: Supplementary file 1 — Supplementary information [file 41467_2026_72836_MOESM1_ESM.pdf]
