# Supplementary material for: Immune surveillance on the insect body surface recognizes a pathogen-derived fungal protease to activate defenses
Source: Nat Commun. 2026 May 9;17:6268. doi: 10.1038/s41467-026-72836-4 (PMC13376424; doi:10.1038/s41467-026-72836-4)
Supplement: Supplementary file 2 — Description of Additional Supplementary Files [file 41467_2026_72836_MOESM2_ESM.pdf]

Title: Supplementary Data 1

Description: Mass spectrometry identification of locust proteins binding to Metarhizium acridum conidia. This file contains the complete list of proteins identified by LC-MS/MS from the conidial affinity chromatography (pull-down) assay, including cell-free hemolymph, integument, and hindwing samples.

Title: Supplementary Data 2

Description: Primer and probe sequences used in this study. This file lists all oligonucleotide sequences utilized for plasmid construction, fungal mutant generation and verification, RT qPCR, dsRNA synthesis, and fluorescence in situ hybridization (FISH).

Title: Supplementary Data 3

Description: Protein accession numbers used for phylogenetic analyses. This file provides the GenBank and relevant database accession numbers for all IML1-like lectins and SP28 serine proteases used to construct the phylogenetic trees in this study.
