# Supplementary material for: Immune surveillance on the insect body surface recognizes a pathogen-derived fungal protease to activate defenses
Source: Nat Commun. 2026 May 9;17:6268. doi: 10.1038/s41467-026-72836-4 (PMC13376424; doi:10.1038/s41467-026-72836-4)
Supplement: Supplementary file 6 — Reporting Summary [file 41467_2026_72836_MOESM6_ESM.pdf]

nature portfolio

Corresponding author(s): Yuxian Xia

Last updated by author(s): Feb 8, 2026

Reporting Summary

Nature Portfolio wishes to improve the reproducibility of the work that we publish. This form provides structure for consistency and transparency in reporting. For further information on Nature Portfolio policies, see our Editorial Policies and the Editorial Policy Checklist.

Statistics

For all statistical analyses, confirm that the following items are present in the figure legend, table legend, main text, or Methods section.

n/a	Confirmed	
☐	☒	The exact sample size (*n*) for each experimental group/condition, given as a discrete number and unit of measurement
☐	☒	A statement on whether measurements were taken from distinct samples or whether the same sample was measured repeatedly
☐	☒	The statistical test(s) used AND whether they are one- or two-sided<br>*Only common tests should be described solely by name; describe more complex techniques in the Methods section.*
☒	☐	A description of all covariates tested
☐	☒	A description of any assumptions or corrections, such as tests of normality and adjustment for multiple comparisons
☐	☒	A full description of the statistical parameters including central tendency (e.g. means) or other basic estimates (e.g. regression coefficient) AND variation (e.g. standard deviation) or associated estimates of uncertainty (e.g. confidence intervals)
☐	☒	For null hypothesis testing, the test statistic (e.g. *F*, *t*, *r*) with confidence intervals, effect sizes, degrees of freedom and *P* value noted<br>*Give P values as exact values whenever suitable.*
☒	☐	For Bayesian analysis, information on the choice of priors and Markov chain Monte Carlo settings
☒	☐	For hierarchical and complex designs, identification of the appropriate level for tests and full reporting of outcomes
☒	☐	Estimates of effect sizes (e.g. Cohen's *d*, Pearson's *r*), indicating how they were calculated

Our web collection on statistics for biologists contains articles on many of the points above.

Software and code

Policy information about availability of computer code

Data collection	Data collection relied on experimental techniques and instrument-specific control software, with no mention of dedicated commercial, open-source, or custom code for data acquisition. Standard laboratory equipment and their accompanying control software were used for operations such as centrifugation, spectrophotometry, and microscopy (e.g., imaging software for the Nikon Eclipse Ci fluorescence microscope). Mass spectrometry data were acquired using an AB Sciex TripleTOF 5600-plus mass spectrometer controlled by the vendor's Analyst® TF software (version 1.8). Thus, no specialized data collection software or code was employed.
Data analysis	(1) SPSS is used for all core statistical analyses, including t-tests, ANOVA, and survival analysis. Version 20.0.0.<br>(2) ImageJ is used for image analysis, e.g., quantification of fluorescence intensity (referred to as "ImageJ analysis"). Version 1.51j8.<br>(3) GraphPad Prism is used to calculate binding affinity (Kd) (mentioned as "Prism GraphPad software"). Version 8.0.1.<br>(4) MEGA is used for phylogenetic tree construction. Version 12.0.14.<br>(5) AutoDock is used for molecular docking analysis. Version 4.2.<br>(6) ProteinPilot is used for LC-MS/MS data analysis (protein identification and peptide mapping). Version 5.0.1 (AB Sciex).

For manuscripts utilizing custom algorithms or software that are central to the research but not yet described in published literature, software must be made available to editors and reviewers. We strongly encourage code deposition in a community repository (e.g. GitHub). See the Nature Portfolio guidelines for submitting code & software for further information.

Data

Policy information about availability of data

All manuscripts must include a data availability statement. This statement should provide the following information, where applicable:

- Accession codes, unique identifiers, or web links for publicly available datasets
- A description of any restrictions on data availability
- For clinical datasets or third party data, please ensure that the statement adheres to our policy

The protein mass spectrometry raw data have been deposited to the ProteomeXchange Consortium via the iProX partner repository with the dataset identifier PXD076064 (http://proteomecentral.proteomexchange.org/cgi/GetDataset?ID=PXD076064). All other data supporting the findings of this study are available within the Article, the Supplementary Information, or Source Data file. The full list of identified proteins from the pull-down assay is provided in Supplementary Data 1. Primer and probe sequences are provided in Supplementary Data 2, and gene accession numbers used for phylogenetic analysis are listed in Supplementary Data 3. All newly generated materials, including the recombinant plasmids, expression vectors, and fungal mutant strains described in this study, are available from the corresponding author upon reasonable request for non-commercial research purposes. Source data are provided with this paper.

Research involving human participants, their data, or biological material

Policy information about studies with human participants or human data. See also policy information about sex, gender (identity/presentation), and sexual orientation and race, ethnicity and racism.

Reporting on sex and gender	This study used invertebrate insects (Locusta migratoria, Spodoptera frugiperda, etc.) as experimental models. No sex- or gender-based analysis was performed, as the research question focuses on conserved surface immune mechanisms not related to sex or gender.
Reporting on race, ethnicity, or other socially relevant groupings	This study did not use any socially constructed or socially relevant categorization variables (e.g., race, ethnicity, gender). The research is in the field of molecular entomology and microbiology, utilizing insect and fungal models; therefore, such human social variables are not relevant to the research question or design. Potential biological confounding was controlled through standardized experimental conditions, use of genetically defined organisms, and appropriate control groups, as detailed in the experimental methods.
Population characteristics	This study did not involve any human research participants. The research is exclusively based on insect and fungal models. Therefore, no covariate-relevant population characteristics (age, genotype, diagnosis, etc.) are applicable.
Recruitment	This study did not involve any human participants. All experiments were conducted using insect models (Locusta migratoria, Spodoptera frugiperda, Nilaparvata lugens, Phyllotreta striolata) and fungal pathogens. Therefore, questions regarding participant recruitment, self-selection bias, or other human subject-related biases are not applicable.
Ethics oversight	The study protocol did not involve human participants or personal data. Experimental work with insect models was conducted in accordance with standard laboratory practices for which specific ethical approval is not required. The use of mice for polyclonal antiserum production was approved by the Animal Ethics Committee of Chongqing University.

Note that full information on the approval of the study protocol must also be provided in the manuscript.

Field-specific reporting

Please select the one below that is the best fit for your research. If you are not sure, read the appropriate sections before making your selection.

[x] Life sciences [ ] Behavioural & social sciences [ ] Ecological, evolutionary & environmental sciences

For a reference copy of the document with all sections, see nature.com/documents/nr-reporting-summary-flat.pdf

Life sciences study design

All studies must disclose on these points even when the disclosure is negative.

Sample size	No formal a priori sample size calculation or statistical power analysis was performed. Sample sizes were chosen based on well-established conventions in the fields of insect pathology and molecular host-microbe interactions. For molecular and biochemical assays (e.g., qRT-PCR, phenoloxidase activity, binding assays), three independent biological replicates was used, as this is the standard to reliably capture biological variation and permit robust statistical comparison (t-test, ANOVA). For survival assays, a sample size of 30 insects per group was employed, which is a common and sufficient cohort size in entomological bioassays to generate reliable Kaplan-Meier survival curves and detect significant differences using log-rank tests. For cellular assays (hemocyte counts, phagocytosis), five biological replicates (n = 5) per condition were used to account for inter-individual variation.These sample sizes are considered adequate based on extensive prior experimental experience in the field and were validated empirically, as they consistently enabled the detection of statistically significant differences (P < 0.05) for the key comparisons central to this study.
Data exclusions	No data were excluded from the analyses.
Replication	All experimental findings were verified for reproducibility through multiple approaches: (1) all experiments were conducted with a minimum of three independent biological replicates, with results presented as mean ± SEM; (2) key experiments were repeated in full at least three times, consistently yielding similar results; (3) central conclusions were supported by converging evidence from complementary techniques

	(e.g., biochemical, genetic, cellular, and in vivo functional assays); and (4) the core mechanism was further validated across multiple fungal pathogens and insect hosts. All attempts at replication were successful, and no findings failed to be reproduced.
Randomization	The allocation of organisms (insects and fungi) into experimental groups was based on the experimental factor being tested (e.g., fungal strain, treatment type, genetic manipulation). Insects of the same developmental stage were randomly selected from a homogeneous, standardized rearing colony and assigned to groups. Fungal strains (wild-type, knockout, complemented, etc.) were cultured under identical conditions prior to inoculation. No formal randomization protocol is described, but allocation aimed to ensure initial equivalence by using organisms of uniform age, size, and rearing history. Covariates such as developmental stage, rearing environment, and inoculation procedures were strictly controlled across all groups through standardized protocols, thereby minimizing potential confounding variation. This approach is standard in comparative functional studies in entomology and microbiology, where the primary source of variation is intentionally introduced by the experimental treatment itself.
Blinding	Blinding was not explicitly implemented or described in the study. In this experimental system, the primary outcomes were objective, instrument-based measurements: phenoloxidase activity (spectrophotometric absorbance), gene expression (qRT-PCR cycle thresholds), hemocyte counts (direct microscopy), and survival (time-to-death recording). These endpoints are largely resistant to observer bias. Image-based quantifications (e.g., fluorescence intensity) were analyzed using standardized software (ImageJ), minimizing subjective assessment. However, during sample processing and data collection, researchers were likely aware of group identities due to the visible nature of treatments (e.g., different fungal strains).

Behavioural & social sciences study design

All studies must disclose on these points even when the disclosure is negative.

Study description	*Briefly describe the study type including whether data are quantitative, qualitative, or mixed-methods (e.g. qualitative cross-sectional, quantitative experimental, mixed-methods case study).*
Research sample	*State the research sample (e.g. Harvard university undergraduates, villagers in rural India) and provide relevant demographic information (e.g. age, sex) and indicate whether the sample is representative. Provide a rationale for the study sample chosen. For studies involving existing datasets, please describe the dataset and source.*
Sampling strategy	*Describe the sampling procedure (e.g. random, snowball, stratified, convenience). Describe the statistical methods that were used to predetermine sample size OR if no sample-size calculation was performed, describe how sample sizes were chosen and provide a rationale for why these sample sizes are sufficient. For qualitative data, please indicate whether data saturation was considered, and what criteria were used to decide that no further sampling was needed.*
Data collection	*Provide details about the data collection procedure, including the instruments or devices used to record the data (e.g. pen and paper, computer, eye tracker, video or audio equipment) whether anyone was present besides the participant(s) and the researcher, and whether the researcher was blind to experimental condition and/or the study hypothesis during data collection.*
Timing	*Indicate the start and stop dates of data collection. If there is a gap between collection periods, state the dates for each sample cohort.*
Data exclusions	*If no data were excluded from the analyses, state so OR if data were excluded, provide the exact number of exclusions and the rationale behind them, indicating whether exclusion criteria were pre-established.*
Non-participation	*State how many participants dropped out/declined participation and the reason(s) given OR provide response rate OR state that no participants dropped out/declined participation.*
Randomization	*If participants were not allocated into experimental groups, state so OR describe how participants were allocated to groups, and if allocation was not random, describe how covariates were controlled.*

Ecological, evolutionary & environmental sciences study design

All studies must disclose on these points even when the disclosure is negative.

Study description	*Briefly describe the study. For quantitative data include treatment factors and interactions, design structure (e.g. factorial, nested, hierarchical), nature and number of experimental units and replicates.*
Research sample	*Describe the research sample (e.g. a group of tagged Passer domesticus, all Stenocereus thurberi within Organ Pipe Cactus National Monument), and provide a rationale for the sample choice. When relevant, describe the organism taxa, source, sex, age range and any manipulations. State what population the sample is meant to represent when applicable. For studies involving existing datasets, describe the data and its source.*
Sampling strategy	*Note the sampling procedure. Describe the statistical methods that were used to predetermine sample size OR if no sample-size calculation was performed, describe how sample sizes were chosen and provide a rationale for why these sample sizes are sufficient.*
Data collection	*Describe the data collection procedure, including who recorded the data and how.*
Timing and spatial scale	*Indicate the start and stop dates of data collection, noting the frequency and periodicity of sampling and providing a rationale for these choices. If there is a gap between collection periods, state the dates for each sample cohort. Specify the spatial scale from which the data are taken*

Data exclusions	*If no data were excluded from the analyses, state so OR if data were excluded, describe the exclusions and the rationale behind them, indicating whether exclusion criteria were pre-established.*
Reproducibility	*Describe the measures taken to verify the reproducibility of experimental findings. For each experiment, note whether any attempts to repeat the experiment failed OR state that all attempts to repeat the experiment were successful.*
Randomization	*Describe how samples/organisms/participants were allocated into groups. If allocation was not random, describe how covariates were controlled. If this is not relevant to your study, explain why.*
Blinding	*Describe the extent of blinding used during data acquisition and analysis. If blinding was not possible, describe why OR explain why blinding was not relevant to your study.*

Did the study involve field work? ☐ Yes ☐ No

Field work, collection and transport

Field conditions	*Describe the study conditions for field work, providing relevant parameters (e.g. temperature, rainfall).*
Location	*State the location of the sampling or experiment, providing relevant parameters (e.g. latitude and longitude, elevation, water depth).*
Access & import/export	*Describe the efforts you have made to access habitats and to collect and import/export your samples in a responsible manner and in compliance with local, national and international laws, noting any permits that were obtained (give the name of the issuing authority, the date of issue, and any identifying information).*
Disturbance	*Describe any disturbance caused by the study and how it was minimized.*

Reporting for specific materials, systems and methods

We require information from authors about some types of materials, experimental systems and methods used in many studies. Here, indicate whether each material, system or method listed is relevant to your study. If you are not sure if a list item applies to your research, read the appropriate section before selecting a response.

Materials & experimental systems

n/a	Involved in the study	
☐	☒	Antibodies
☒	☐	Eukaryotic cell lines
☒	☐	Palaeontology and archaeology
☐	☒	Animals and other organisms
☒	☐	Clinical data
☒	☐	Dual use research of concern
☒	☐	Plants

Methods

n/a	Involved in the study	
☒	☐	ChIP-seq
☒	☐	Flow cytometry
☒	☐	MRI-based neuroimaging

Antibodies

Antibodies used	The following antibodies were used: A purified, custom-made mouse polyclonal anti-IML1 antibody (described in 'Antibody preparation'). Commercial mouse monoclonal anti-FLAG antibody (Beyotime, #AF2852). HRP-conjugated goat anti-mouse IgG (Beyotime, #A0216). Alexa Fluor 594-conjugated goat anti-mouse IgG (Thermo Fisher, #A-11005). FITC-conjugated goat anti-rabbit IgG (Beyotime, #A0562). 35-nm gold-conjugated goat anti-mouse IgG (Solarbio, #K1031R-G35). Normal mouse IgG isotype control (Beyotime, #A7028). Rabbit polyclonal anti-MBL2 antibody (Beyotime, #AF7428). For mannan blocking: Mannan-Binding Lectin, MBL (HY-MedChemExpress, #P70674).
Validation	Custom-made anti-IML1 polyclonal antibody (mouse): Validation for application in Western blot (WB) and immunofluorescence (IF) on locust (Locusta migratoria) samples is provided within the manuscript. Specificity was confirmed by WB against locust hemocyte protein extract, showing a single major band at the expected molecular weight (Supplementary Fig. 3b). Functional validation is supported by the congruence of results from multiple independent methods (RNAi, antibody blocking, recombinant protein) targeting IML1, and by the specific localization patterns observed in IF and immunogold assays (e.g., Fig. 3b-c, Supplementary Fig. 4b-c).<br>Commercial anti-FLAG monoclonal antibody (mouse, Beyotime #AF2852): Validation relies on the manufacturer's specifications. According to the supplier's datasheet, this antibody is validated for applications including WB, IF, and immunoprecipitation (IP) for the detection of C-terminal FLAG-tagged fusion proteins. Its successful application for IF on fungal conidia in this study is demonstrated in Supplementary Fig. 8c-d and 11c.<br>Mannan-Binding Lectin (MBL) and anti-MBL2 antibody: The MBL protein (HY-MedChemExpress #P70674) is a defined lectin with inherent specificity for mannan polysaccharides, used here as a blocking and detection reagent. Its binding to fungal conidia was detected using a commercial rabbit polyclonal anti-MBL2 antibody (Beyotime, #AF7428), the specificity of which is supported by the

manufacturer's validation for IF. The effective blocking of IML1 binding by MBL (Fig. 2e-f) and the specific IF signal for MBL on conidia empirically validate the use of this detection system in our experimental context.

Eukaryotic cell lines

Policy information about cell lines and Sex and Gender in Research

Cell line source(s)	*State the source of each cell line used and the sex of all primary cell lines and cells derived from human participants or vertebrate models.*
Authentication	*Describe the authentication procedures for each cell line used OR declare that none of the cell lines used were authenticated.*
Mycoplasma contamination	*Confirm that all cell lines tested negative for mycoplasma contamination OR describe the results of the testing for mycoplasma contamination OR declare that the cell lines were not tested for mycoplasma contamination.*
Commonly misidentified lines (See ICLAC register)	*Name any commonly misidentified cell lines used in the study and provide a rationale for their use.*

Palaeontology and Archaeology

Specimen provenance	*Provide provenance information for specimens and describe permits that were obtained for the work (including the name of the issuing authority, the date of issue, and any identifying information). Permits should encompass collection and, where applicable, export.*
Specimen deposition	*Indicate where the specimens have been deposited to permit free access by other researchers.*
Dating methods	*If new dates are provided, describe how they were obtained (e.g. collection, storage, sample pretreatment and measurement), where they were obtained (i.e. lab name), the calibration program and the protocol for quality assurance OR state that no new dates are provided.*

☐ Tick this box to confirm that the raw and calibrated dates are available in the paper or in Supplementary Information.

Ethics oversight	*Identify the organization(s) that approved or provided guidance on the study protocol, OR state that no ethical approval or guidance was required and explain why not.*

Note that full information on the approval of the study protocol must also be provided in the manuscript.

Animals and other research organisms

Policy information about studies involving animals; ARRIVE guidelines recommended for reporting animal research, and Sex and Gender in Research

Laboratory animals	This study did not involve vertebrate laboratory animals in its core experimental design. All primary experiments utilized invertebrate insect models: fifth-instar nymphs or adults of the migratory locust (Locusta migratoria), second-instar larvae of the fall armyworm (Spodoptera frugiperda), and adults of the rice planthopper (Nilaparvata lugens) and flea beetle (Phyllotreta striolata), all reared under standardized laboratory conditions. The sole use of vertebrate animals was for reagent generation, BALB/C female mice (6–8 weeks old) used for polyclonal antibody production were housed under specific pathogen-free conditions at the Experimental Animal Center of Chongqing University, with a 12-h light : 12-h dark cycle, ambient temperature of 22 ± 2 °C, and relative humidity of 50 ± 5 %. Animals were provided with standard chow and water ad libitum.
Wild animals	The study did not involve the observation or capture of wild animals. All insects used in the experiments (Locusta migratoria, Spodoptera frugiperda, Nilaparvata lugens, Phyllotreta striolata) were from laboratory-reared colonies maintained under controlled conditions. The fungal strains were cultured from existing laboratory collections. Therefore, no procedures related to the catching, transport, or post-study disposition of wild animals were applicable.
Reporting on sex	This study did not involve sex-based analysis
Field-collected samples	This study did not involve any samples collected from the field.
Ethics oversight	This study primarily utilized invertebrate insects, for which formal ethical committee approval is not typically required under international and institutional guidelines. The immunization of mice for antiserum production is a standard procedure that generally requires approval from the Experimental Animal Ethics Committee of Chongqing University.

Note that full information on the approval of the study protocol must also be provided in the manuscript.

Clinical data

Policy information about clinical studies
All manuscripts should comply with the ICMJE guidelines for publication of clinical research and a completed CONSORT checklist must be included with all submissions.

Clinical trial registration	*Provide the trial registration number from ClinicalTrials.gov or an equivalent agency.*

Study protocol	*Note where the full trial protocol can be accessed OR if not available, explain why.*
Data collection	*Describe the settings and locales of data collection, noting the time periods of recruitment and data collection.*
Outcomes	*Describe how you pre-defined primary and secondary outcome measures and how you assessed these measures.*

Dual use research of concern

Policy information about dual use research of concern

Hazards

Could the accidental, deliberate or reckless misuse of agents or technologies generated in the work, or the application of information presented in the manuscript, pose a threat to:

No	Yes	
☐	☐	Public health
☐	☐	National security
☐	☐	Crops and/or livestock
☐	☐	Ecosystems
☐	☐	Any other significant area

Experiments of concern

Does the work involve any of these experiments of concern:

No	Yes	
☐	☐	Demonstrate how to render a vaccine ineffective
☐	☐	Confer resistance to therapeutically useful antibiotics or antiviral agents
☐	☐	Enhance the virulence of a pathogen or render a nonpathogen virulent
☐	☐	Increase transmissibility of a pathogen
☐	☐	Alter the host range of a pathogen
☐	☐	Enable evasion of diagnostic/detection modalities
☐	☐	Enable the weaponization of a biological agent or toxin
☐	☐	Any other potentially harmful combination of experiments and agents

Plants

Seed stocks	This study did not utilize any seed stocks or plant material.
Novel plant genotypes	N/A
Authentication	N/A

ChIP-seq

Data deposition

☐ Confirm that both raw and final processed data have been deposited in a public database such as GEO.

☐ Confirm that you have deposited or provided access to graph files (e.g. BED files) for the called peaks.

Data access links<br>*May remain private before publication.*	*For "Initial submission" or "Revised version" documents, provide reviewer access links. For your "Final submission" document, provide a link to the deposited data.*
Files in database submission	*Provide a list of all files available in the database submission.*

Genome browser session
(e.g. UCSC)

Provide a link to an anonymized genome browser session for "Initial submission" and "Revised version" documents only, to enable peer review. Write "no longer applicable" for "Final submission" documents.

Methodology

Replicates

Describe the experimental replicates, specifying number, type and replicate agreement.

Sequencing depth

Describe the sequencing depth for each experiment, providing the total number of reads, uniquely mapped reads, length of reads and whether they were paired- or single-end.

Antibodies

Describe the antibodies used for the ChIP-seq experiments; as applicable, provide supplier name, catalog number, clone name, and lot number.

Peak calling parameters

Specify the command line program and parameters used for read mapping and peak calling, including the ChIP, control and index files used.

Data quality

Describe the methods used to ensure data quality in full detail, including how many peaks are at FDR 5% and above 5-fold enrichment.

Software

Describe the software used to collect and analyze the ChIP-seq data. For custom code that has been deposited into a community repository, provide accession details.

Flow Cytometry

Plots

Confirm that:

- [ ] The axis labels state the marker and fluorochrome used (e.g. CD4-FITC).
- [ ] The axis scales are clearly visible. Include numbers along axes only for bottom left plot of group (a 'group' is an analysis of identical markers).
- [ ] All plots are contour plots with outliers or pseudocolor plots.
- [ ] A numerical value for number of cells or percentage (with statistics) is provided.

Methodology

Sample preparation

Describe the sample preparation, detailing the biological source of the cells and any tissue processing steps used.

Instrument

Identify the instrument used for data collection, specifying make and model number.

Software

Describe the software used to collect and analyze the flow cytometry data. For custom code that has been deposited into a community repository, provide accession details.

Cell population abundance

Describe the abundance of the relevant cell populations within post-sort fractions, providing details on the purity of the samples and how it was determined.

Gating strategy

Describe the gating strategy used for all relevant experiments, specifying the preliminary FSC/SSC gates of the starting cell population, indicating where boundaries between "positive" and "negative" staining cell populations are defined.

- [ ] Tick this box to confirm that a figure exemplifying the gating strategy is provided in the Supplementary Information.

Magnetic resonance imaging

Experimental design

Design type

Indicate task or resting state; event-related or block design.

Design specifications

Specify the number of blocks, trials or experimental units per session and/or subject, and specify the length of each trial or block (if trials are blocked) and interval between trials.

Behavioral performance measures

State number and/or type of variables recorded (e.g. correct button press, response time) and what statistics were used to establish that the subjects were performing the task as expected (e.g. mean, range, and/or standard deviation across subjects).

Acquisition

Imaging type(s) | *Specify: functional, structural, diffusion, perfusion.*

Field strength | *Specify in Tesla*

Sequence & imaging parameters | *Specify the pulse sequence type (gradient echo, spin echo, etc.), imaging type (EPI, spiral, etc.), field of view, matrix size, slice thickness, orientation and TE/TR/flip angle.*

Area of acquisition | *State whether a whole brain scan was used OR define the area of acquisition, describing how the region was determined.*

Diffusion MRI ☐ Used ☐ Not used

Preprocessing

Preprocessing software | *Provide detail on software version and revision number and on specific parameters (model/functions, brain extraction, segmentation, smoothing kernel size, etc.).*

Normalization | *If data were normalized/standardized, describe the approach(es): specify linear or non-linear and define image types used for transformation OR indicate that data were not normalized and explain rationale for lack of normalization.*

Normalization template | *Describe the template used for normalization/transformation, specifying subject space or group standardized space (e.g. original Talairach, MNI305, ICBM152) OR indicate that the data were not normalized.*

Noise and artifact removal | *Describe your procedure(s) for artifact and structured noise removal, specifying motion parameters, tissue signals and physiological signals (heart rate, respiration).*

Volume censoring | *Define your software and/or method and criteria for volume censoring, and state the extent of such censoring.*

Statistical modeling & inference

Model type and settings | *Specify type (mass univariate, multivariate, RSA, predictive, etc.) and describe essential details of the model at the first and second levels (e.g. fixed, random or mixed effects; drift or auto-correlation).*

Effect(s) tested | *Define precise effect in terms of the task or stimulus conditions instead of psychological concepts and indicate whether ANOVA or factorial designs were used.*

Specify type of analysis: ☐ Whole brain ☐ ROI-based ☐ Both

Statistic type for inference | *Specify voxel-wise or cluster-wise and report all relevant parameters for cluster-wise methods.*

(See Eklund et al. 2016)

Correction | *Describe the type of correction and how it is obtained for multiple comparisons (e.g. FWE, FDR, permutation or Monte Carlo).*

Models & analysis

n/a	Involved in the study
☐	☐ Functional and/or effective connectivity
☐	☐ Graph analysis
☐	☐ Multivariate modeling or predictive analysis

Functional and/or effective connectivity | *Report the measures of dependence used and the model details (e.g. Pearson correlation, partial correlation, mutual information).*

Graph analysis | *Report the dependent variable and connectivity measure, specifying weighted graph or binarized graph, subject- or group-level, and the global and/or node summaries used (e.g. clustering coefficient, efficiency, etc.).*

Multivariate modeling and predictive analysis | *Specify independent variables, features extraction and dimension reduction, model, training and evaluation metrics.*
