# Supplementary material for: Immune surveillance on the insect body surface recognizes a pathogen-derived fungal protease to activate defenses
Source: Nat Commun. 2026 May 9;17:6268. doi: 10.1038/s41467-026-72836-4 (PMC13376424; doi:10.1038/s41467-026-72836-4)
Supplement: Supplementary file 7 — Transparent Peer Review file [file 41467_2026_72836_MOESM7_ESM.pdf]

nature portfolio

Peer Review File

# Immune surveillance on the insect body surface recognizes a pathogen-derived fungal protease to activate defenses

Corresponding Author: Professor Yuxian Xia

**This file contains all reviewer reports in order by version, followed by all author rebuttals in order by version.**

Version 0:

Reviewer comments:

Reviewer #1

(Remarks to the Author)
Li et al. performed comprehensive experiments aiming to establish a lectin-sensing and protease-gated surveillance model occurring on locust body surface to discriminate the pathogenic and non-pathogenic fungal spore. An immune lectin (IML1) was identified with a higher-level of binding affinity to mannan using the pathogenic fungal spores for immunoprecitation. Intriguingly, the authors showed that IML1 can similarly bind non-pathogenic Aspergillus spores, the latter cannot induce extensive immune activation. It was further shown by the authors that the Metarhizium spore surface protease ManSP28/MacSP28 contributes to cleaving IML1 into fragments, and the smaller fragment IML1an re-enters into locust body cavity to trigger antifungal immune defenses. Overall, the results sound interesting and novel if the solid and compelling evidence has been provided in the core steps.

Specific comments:
First, the so-called “dual-senor mechanism” is confusing and misleading since fungal protease was claimed to get involved rather than both factors are from insect hosts. It is different from the dual sensing mechanism like the Drosophila GNBP3-Psh system. The following question would be why the entomopathogen evolved the negative virulence factor ManSP28/MacSP28 to help activate host immune reactions. Even negative virulence factors have been frequently reported, the related literatures have been cited and the point has not been discussed.

(Core) Experimental procedures have not been clearly described including the experimental setup. Started from line 70 “inoculated to attach on cuticle”, “host surface”, “locust cuticle” or “integument” etc. has been generally described by the authors. There are no details of inoculation: pronotum, thorax, abdomen or abdominal segment? It is doubted that the rigid, hydrophobic, waxy pronotum, thorax or abdomen surfaces can have any sensor protein or enzyme activity. It is unclear from the Results, figure legends and even not clear from Methods regarding the experimental details of spore inoculations: spore dosage, inoculation methods and time post inoculation to dissect locust cuticles for PO enzyme activity and defensin expression analysis? It is not wise to ask people to go for their previous publications for details.

For the setup of affinity chromatography “using total protein extracted from locust wings (including epicuticle and hemolymph), epicuticle (excluding hemolymph), and cell-free hemolymph (excluding epicuticle and hemocytes)” (Lines 100-102) could not guarantee the subsequently obtained IML1 is a host surface protein. Why not a hemolymph surveillance lectin? However, “Surface IML1” was concluded and took for granted from line 139 section onward.

It is tricky that mannan, glucan and chitosan (all commercial origins are unclear) but not chitin (the real fungal cell wall component) were used in binding assays. IML1 for chitin cannot be precluded at this stage.

For the essential results shown in Fig. 3A-C, it is not clear where were these surfaces from or referred to. In addition, immunogold labelling method has not been described, and the evidence of IML1 presence on surface (not clear where exactly is) is slim. How to confirm the arrowed white spots to be IML1 in Fig. 3C. On top of FISH analysis (Fig. 3A), immunofluorescent analysis using the obtained antibody would be more direct since the authors have shown that the expression of IML1 is not high in epidermal cells (Fig. S3C).

Critically, what are the IML1an/IMl1ac peptide sequences, are they the same? No LC-MS/MS analysis has been conducted.

Thus, how the IML1an-His tagged polypeptide/protein was prepared for Fig. S9. It is awkward to peptide and fungal spore challenges in the same panels. The applied protein amount for Fig. 4D-F (yellow parts) are unclear.

Fig. 5. It is unfortunately that the authors have missed the inclusion of OE mutant WT::ManSP28 mutant for parallel survival assays. This gain-of-function result would be essential to support the conclusion. In addition, unlike glucan and chitin/chitosa, commercial mannans may be water soluble. Its direct influence on insect survivals is unclear. A control treatment using pure mannan has not been included.

The authors have not shown that ManSP28/MacSO28 is highly transcribed in fungal conidia. Not as described (lines 238-239), SP28 signal could not or be barely seen on the WT spore surface.

The authors have shown that Aspergillus flavus does have a ManSP28 homolog, however, which has not been expressed for IML1 cleavage assay. It may not localize on conidial surface.

There are also structuring and writing issues. The conclusive diagram Fig. S10 is put in supplementary, which appears before the last paragraph. The phylogenies of IML1 and ManSP28 proteins can be shown in supplementary just following their identifications. The authors show Fig. 5F and Fig. S6B, both for SP28 proteases. There is no results of molecular docking described in Methods.

Immulectin-1 for IML1 has not been indicated in Abstract etc. until the appearance in line 112.

Line 190-191, two Fig. S5F? Fig. S5E, what are those abbreviations?

The labels "IML1ac0/an0" and "IML1ac/an" could be briefly defined in the main text and figure legend for clarity.

Line 42: "through distincting from those against invasive pathogen." This phrase is grammatically incorrect. It should likely read something like: "through mechanisms distinct from those used against invasive pathogens" or "by distinguishing them from invasive pathogens."

Line 269-270: "SP28 delaying fungal infection through triggers host defences, thereby." This sentence is incomplete. It should be rephrased, e.g., "SP28 delays fungal infection by triggering host defenses."

Line 322: "This integrated systems is may represent..." change to "This integrated system may represent..."

While generally clear, some figure captions (e.g., Fig. S10) could be slightly more descriptive for readers glancing at the supplement.

Reviewer #2

(Remarks to the Author)
In this report, the authors identify an insect host lectin (IML1), expressed near the cuticle surface that recognizes mannans. The authors show that IML1 functions to activate immune responses only if cleaved by a fungal protease designated SP28. Although most fungi appear to contain SP28, only the SP28 from (some?) entomopathogenic fungi appears to be able to cleave IML1 into an active form which subsequently results in immune activation.

The report is very interesting, and the experiments well performed.

One conceptual issue should be addressed in the Discussion: why would the insect pathogen retain SP28 since its ablation increases virulence and conversely why would non-pathogens lack “active” SP28 since they aren’t pathogens to begin with?

In addition:

1. What is the time point for the data in Fig. 1?
2. IML1 appears to have reasonable binding to glucans and lower binding to chitosan, but chitin (hydroxymethylchitin is more soluble) was not tested. What about response to glucans which are prevalent on fungal surfaces?
3. What is the “stain” in Fig. 1D and why does A. alternata show no staining. Binding of the recombinant IML1 appears to be lower to A. flavus (Fig. 2). Can the signal be measured to provide a more quantified analysis?
4. The background control in Fig 3B is very high (compare to the background/signal in Fig. S4B). More importantly, shouldn’t the samples be examined as a cross-section as given in panel A? We should see a similar pattern between the mRNA and antibody? How is IML1 anchored to the cuticle, it appears to be a soluble protein?
5. B. bassiana should be included in Fig. 4A. Figure legend should include time points used for each panel.
6. According to Fig. 5F, the “active” Clade G SP28 includes plant pathogenic fungi. Does the SP28 from these result in IML1 activation? If so, that would make the issue of specificity more complex. Related: Panels E and F are not that informative as few organisms are labeled. It may be better to indicate which Class/Order either IML1 and/or SP8 are present. At which point do the authors consider specificity to have been affected?
7. Data for the SP28 complemented mutant appears to be missing to confirm the genetics.
8. Considering Fig. S6B, can the P. lilacinum and/or B. bassiana but not A. flavus and/or A. alternata SP28 genes complement the SP28 mutant in M. acridum?
9. Are there any other phenotypic consequences of loss of SP28 for the fungus? This would relate to why it may be

maintained.
10. IML1 appears to be most highly expressed/induced in hemocytes (Fig. S3C). How does this fit with the model the authors suggest that it's activity is mainly near/underneath the cuticle? Examining slightly longer time points, i.e. 12-18 h may also be informative as this would represent spore germination?
11. The timing of IML1 expression (Fig. 3) seems confusing. IML1 expression is low to begin with but is induced to high levels at 4 hr
12. Do heat killed M. acridum spores +/- SP8 bind to IML1 and/or activate immune responses? This would seem to be an important control.
13. B. bassiana should be included in Fig. 4A.

Version 1:

Reviewer comments:

Reviewer #1

(Remarks to the Author)
Additional experiments and repetition of some experiments have been done in this revision. Taken together with re-writing and re-structuring, substantial improvements have been made, and the answers of concerns are generally acceptable. Remaining corrections and clarifications are still required.

There are issues for the reported sequences/accessions. Table S1, LOCMI10396 is not a lectin. Line 263, EFY85443 for MacSP28 but Table S3 shows that EFY87133 is for ManSP28 (sic). EVM0008168 is not accessible in public databases.

The remaining controversy results are the data regarding the ManSP28/MacP28's contribution to invasion and IML1 cleavage. Fig. S9 data indicated that KO strain was impaired in penetrating locust wings (implication in degrading cuticular compounds. In contrast, however, locust survival data showed in Fig. 5 indicated that the LT50 of KO strain was reduced whereas the LT50 of OE strain was increased, i.e., the virulence of KO strain was increased but OE strain had reduced virulence. Even though the data fit with SP28's role in cleaving IML1 to activate locust immune responses, the results did not align with its proposed "additional" involvement in cuticle penetration and host invasion.

The proposed "lock-and-key" model is acceptable but it is confusing regarding the discussion/suggestion (lines 430-444), which argued that "the primary role of SP28 is not to engage the host immune system, but to perform a fundamental physiological function for the fungus." or "host immunity activation is not SP28's primary function, but rather an unavoidable consequence of its essential activity during infection. "

Line 171, "IML1 levels significantly increased in the integument" (Fig. S3d). Actually, band intensity has not been quantified, and it looks that the increase of band intensity is marginal.

Lines 178-179, Fig. S4b is not a TEM analysis.

Fig. S6: x-axis labelings are missed in different panels.

Reviewer #2

(Remarks to the Author)
The authors have done an excellent job at addressing the previous concerns and have supplemented the work with significant new data.

The only minor item concerns the title which is a bit difficult to understand. Would suggest altering to:

1. Insect hosts recognize a fungal pathogen derived protease to activate immune defenses

or

2. Immune surveillance on the insect body surface recognizes a pathogen-derived fungal protease to activate defenses

**Open Access** This Peer Review File is licensed under a Creative Commons Attribution 4.0 International License, which permits use, sharing, adaptation, distribution and reproduction in any medium or format, as long as you give appropriate credit to the original author(s) and the source, provide a link to the Creative Commons license, and indicate if changes were made.
In cases where reviewers are anonymous, credit should be given to 'Anonymous Referee' and the source.
The images or other third party material in this Peer Review File are included in the article's Creative Commons license, unless indicated otherwise in a credit line to the material. If material is not included in the article's Creative Commons license and your intended use is not permitted by statutory regulation or exceeds the permitted use, you will need to obtain permission directly from the copyright holder.
To view a copy of this license, visit https://creativecommons.org/licenses/by/4.0/

**Reviewer #1 (Remarks to the Author):**

Li et al. performed comprehensive experiments aiming to establish a lectin-sensing and protease-gated surveillance model occurring on locust body surface to discriminate the pathogenic and non-pathogenic fungal spore. An immune lectin (IML1) was identified with a higher-level of binding affinity to mannan using the pathogenic fungal spores for immunoprecitation. Intriguingly, the authors showed that IML1 can similarly bind non-pathogenic Aspergillus spores, the latter cannot induce extensive immune activation. It was further shown by the authors that the Metarhizium spore surface protease ManSP28/MacSP28 contributes to cleaving IML1 into fragments, and the smaller fragment IML1an re-enters into locust body cavity to trigger antifungal immune defenses. Overall, the results sound interesting and novel if the solid and compelling evidence has been provided in the core steps.

**Response**: We thank the reviewer for their constructive feedback. To provide the solid evidence requested for the core steps, we have conducted key additional experiments. First, using the SunTag live-imaging system, we directly visualized the translocation of IML1 from the hemolymph to the cuticle surface. Second, phenotypic assays revealed that SP28 deletion impairs fungal growth on the host cuticle, clarifying its essential role in invasion and its unavoidable trigger of host immunity. Furthermore, we introduced critical genetic and biochemical controls, including complementation of the SP28 mutant, heterologous substitution with orthologs from Beauveria bassiana or Aspergillus flavus to test protease specificity, and the use of heat-inactivated spores to decisively separate binding from immune activation. We have also restructured the manuscript to improve logical flow, separating phylogenetic and bioassay results, and moved the model schematic to the main text. Numerous other details have been revised accordingly. We believe these additions and edits have substantially strengthened the support for the proposed mechanism.

Specific comments:

First, the so-called “dual-senor mechanism” is confusing and misleading since fungal protease was claimed to get involved rather than both factors are from insect hosts. It is different from the dual sensing mechanism like the Drosophila GNBP3-Psh system. The following question would be why the entomopathogen evolved the negative virulence factor ManSP28/MacSP28 to help activate host immune reactions. Even negative virulence factors have been frequently reported, the related literatures have been cited and the point has not been discussed.

**Response**: We thank the reviewer for this critical insight. We fully agree that our original terminology was imprecise and have revised the title to “**A pathogen-derived fungal protease gates checkpoint to mediate immune surveillance on insect body surface**” and text throughout to frame our discovery as a “fungal protease-gated checkpoint.” This accurately reflects the mechanism: a host sensor (IML1) whose signal is licensed by a pathogen-derived protease (SP28).

Regarding the evolutionary paradox of why a pathogen would retain an immune-activating factor, our new data provide a clear explanation. We found that SP28 is not a dedicated immune elicitor but an essential colonization factor for the fungus. While the knockout mutant (KO-*ManSP28*) grew normally on nutrient-rich artificial medium, it was significantly impaired (15.8% reduction in colony diameter) when growing on the locust cuticle, a nutrient-poor substrate (Supplementary Fig. 9). Thus, the selective pressure to maintain cuticle-degrading capacity outweighs the cost of alerting the host immune system.

This finding reshapes the discussion of SP28 as a classic “avirulence” or “negative virulence” factor. We have expanded the Discussion (Lines 430-444) to position SP28 within the broader context of pathogen-derived signals. We

propose that its primary role is physiological (facilitating invasion), and the activation of host immunity is an unavoidable consequence that the host has evolutionarily co-opted as a reliable danger signal. This moves beyond the binary “virulence factor/avirulence factor” paradigm toward a more integrated view of co-evolution.

(Core) Experimental procedures have not been clearly described including the experimental setup. Started from line 70 “inoculated to attach on cuticle”, “host surface”, “locust cuticle” or “integument” etc. has been generally described by the authors. There are no details of inoculation: pronotum, thorax, abdomen or abdominal segment? It is doubted that the rigid, hydrophobic, waxy pronotum, thorax or abdomen surfaces can have any sensor protein or enzyme activity. It is unclear from the Results, figure legends and even not clear from Methods regarding the experimental details of spore inoculations: spore dosage, inoculation methods and time post inoculation to dissect locust cuticles for PO enzyme activity and defensin expression analysis? It is not wise to ask people to go for their previous publications for details.

**Response**: We thank the reviewer for raising this important point regarding the physiological feasibility of protein-based interactions on the hydrophobic insect cuticle. We agree that the epicuticle presents a waxy, hydrophobic barrier, which makes our findings of a surface-localized immune surveillance system particularly significant. Our conclusion is robustly supported by a combination of direct experimental evidence, established anatomical principles, and well-documented natural mechanisms that overcome surface hydrophobicity. Below, we address the concern by integrating these lines of evidence into a coherent argument.

We have substantially expanded the Methods section to ensure the work is fully reproducible without recourse to previous publications. All critical experimental details are now explicitly provided, including the precise site,

method, dosage, and timeline of inoculation. Specifically, a 5 μL droplet was applied to the second and third abdominal segments of *L. migratoria*. Furthermore, the specific time points for measuring immune parameters, such as PO activity and defensin expression, are now clearly stated within the relevant Results descriptions and figure legends. For instance, it is now explicitly indicated that the data presented in Fig. 1 were obtained 4 hours post-inoculation.

The cuticle is a dynamic, protein-secreting tissue. Insect cuticle is not a static and impermeable barrier, in fact, is a living and metabolically active tissue. Crucially, it is traversed by pore canals and dermal gland ducts that extend from the underlying epidermis through the procuticle to the epicuticle surface. As reviewed by Moussian (2010), these microscopic channels serve as dedicated anatomical pathways not only for wax secretion but also for the active transport of proteins and enzymes (e.g., esterases) to the surface. This provides a clear and direct physiological route for the secretion and localization of host-derived immune factors like IML1 to the very site of fungal challenge.

Our use of an aqueous spore suspension containing Tween-80 and glycerol in specific assays was a deliberate and necessary design to enable and observe the molecular interactions central to our hypothesis. This formulation achieves two key objectives: It transiently overcomes the inherent hydrophobicity of the thin epicuticular wax layer (1-5 μm). It establishes a stable aqueous phase at the spore-cuticle interface. This controlled microenvironment is essential to permit the solubility, diffusion, and interaction between the host immune protein (IML1) and the fungal protease (SP28). It thereby replicates the fundamental aqueous requirement for any biochemical reaction, which must also be met in nature for such interactions to occur.

In natural environments, the hydrophobic barrier is routinely circumvented through evolved biological mechanisms that create a localized hydrophilic

microenvironment conducive to infection and defense. On the one hand, fungi actively secrete adhesive proteins and mucilage upon contact with the cuticle. These secretions bridge the spore-surface interface, facilitating both hydration and firm adhesion, thereby creating a localized niche for further development (Wang & St. Leger, 2007). On the other hand, insects continuously lose water through the cuticle via Transepidermal Water Loss (TEWL). This process creates a persistent microscopic “boundary layer” of higher humidity immediately adjacent to the cuticle surface (Willmer, 1982). This naturally occurring microclimate provides the essential aqueous medium that enables biochemical recognition and enzymatic activity at the host-pathogen interface.

The insect cuticle surface is a well-documented site of biochemical activity during fungal pathogenesis. As extensively summarized by Ortiz-Urquiza & Keyhani (2013), the epicuticle is the primary battlefield where fungal hydrolytic enzymes are actively engaged. This includes lipases and esterases that degrade the waxy layer itself, and proteases (such as the well-characterized Pr1) that degrade cuticular proteins. The activity of the fungal protease SP28 in our model is entirely consistent with this established paradigm of surface-focused enzymology. The presence and function of IML1 as a surface-localized immune sentinel represent the host’s co-evolved counterpart within this active interfacial landscape.

For the setup of affinity chromatography “using total protein extracted from locust wings (including epicuticle and hemolymph), epicuticle (excluding hemolymph), and cell-free hemolymph (excluding epicuticle and hemocytes)” (Lines 100-102) could not guarantee the subsequently obtained IML1 is a host surface protein. Why not a hemolymph surveillance lectin? However, “Surface IML1” was concluded and took for granted from line 139 section onward.

**Response**: We thank the reviewer for raising this essential point regarding the interpretation of our affinity chromatography data. The reviewer is correct: this biochemical approach using tissue extracts containing both hemolymph

and cuticular components cannot distinguish whether IML1 is a resident surface protein or a soluble hemolymph protein that binds spores *ex vivo*. We therefore did not rely on this assay alone to assign surface localization.

Our conclusions are instead grounded in direct, complementary visualization experiments: Immunogold SEM and SunTag tracing experiment. These data support a revised model for IML1 in *Locusta migratoria*: while IML1 homologs are known to function in hemolymph immunity in other insects (e.g., as a lectin promoting cellular defense), our findings reveal an additional, inducible role at the host-environment interface. We propose that IML1, produced by hemocytes and possibly epidermal cells, is secreted into the hemolymph and can be translocated to the cuticle surface where it performs pathogen surveillance.

Accordingly, we agree that the term "Surface IML1" in the original subheading was imprecise, as it implied a static, exclusive surface residence. We have changed the subheading to "IML1 localizes to the cuticle surface to mediate antifungal defense" and revised the text to clarify that IML1's presence at the surface is dynamic and replenished from internal pools, aligning with the integrated defense strategy we describe.

It is tricky that mannan, glucan and chitosan (all commercial origins are unclear) but not chitin (the real fungal cell wall component) were used in binding assays. IML1 for chitin cannot be precluded at this stage.

**Response**: We thank the reviewer for this important technical point. The reviewer is correct that chitin is the predominant and relevant fungal cell wall polymer, whereas chitosan is a deacetylated derivative not abundant in fungal spores. We have now specified the commercial source and catalog numbers for all polysaccharides (mannan, glucan, chitin) in the Materials and Methods section (Lines 716-717) to ensure reproducibility. As suggested, we have replaced the chitosan-binding data with a direct assessment of IML1's affinity

for chitin. The measured dissociation constant (Kd = 80.30 μg/mL) confirms that IML1 does bind chitin, but with an order-of-magnitude lower affinity compared to mannan (Kd = 2.24 μg/mL) (Fig. 2a-c).

For the essential results shown in Fig. 3A-C, it is not clear where were these surfaces from or referred to. In addition, immunogold labelling method has not been described, and the evidence of IML1 presence on surface (not clear where exactly is) is slim. How to confirm the arrowed white spots to be IML1 in Fig. 3C. On top of FISH analysis (Fig. 3A), immunofluorescent analysis using the obtained antibody would be more direct since the authors have shown that the expression of IML1 is not high in epidermal cells (Fig. S3C).

**Response**: We thank the reviewer for these important points. The manuscript has been revised to clarify all experimental details and to strengthen the evidence for IML1 surface localization. We now specify in the Figure legends and Methods that all cuticle samples were from the second and third abdominal segments.

The Methods now detail the immunogold protocol. A critical negative control (normal mouse IgG) shows no such particles (Supplementary Fig. 4d), confirming the specificity of the signals in Fig. 3c. Direct measurement of these bright spots under high magnification confirmed a diameter of 35 nm, matching the colloidal gold conjugates used. Due to their high atomic number and strong electron-scattering ability, the dense gold nanoparticles appear as distinct white spots under standard SEM imaging, enabling precise nanoscale protein localization on uncoated samples (Miller et al., 2024, *Nat Commun*).

As suggested, we provide direct protein-level visualization via immunofluorescence with our anti-IML1 antibody (Fig. 3b), complementing the FISH data (Fig. 3a). Additionally, the SunTag live-tracing system (Supplementary Fig. 5) provides dynamic, independent evidence. This system employs a 12×GCN4 peptide tag on IML1 and a GFP-labeled single-chain

antibody fragment (scFv) for detection. Specifically, after injecting IML1-12×GCN4 into the hemocoel and allowing 12 hours for distribution, we topically applied fungal conidia. Subsequent dissection, staining with ScFv-GFP, and fluorescence microscopy clearly revealed GFP signals (indicating IML1) on the outer cuticle surface, accumulating around the attached spores. This direct visualization powerfully reinforces the immunogold-SEM results by demonstrating the active recruitment of IML1 from the hemolymph to the precise site of fungal challenge on the outer cuticle.

Critically, what are the IML1an/IMl1ac peptide sequences, are they the same? No LC-MS/MS analysis has been conducted. Thus, how the IML1an-His tagged polypeptide/protein was prepared for Fig. S9. It is awkward to peptide and fungal spore challenges in the same panels. The applied protein amount for Fig. 4D-F (yellow parts) are unclear.

**Response**: We apologize for the lack of clarity. The IML1an and IML1ac peptides have distinct sequences resulting from cleavage at different sites by their respective proteases. The LC-MS/MS analysis was performed on the cleavage products to identify the protease cleavage site on IML1, which then allowed us to deduce the peptide sequences (Supplementary Fig. 7). The His-tagged IML1an peptide used in Supplementary Fig. 12 was produced recombinantly in *E. coli* based on this deduced sequence. We have clarified this process in the Materials and Methods under "Small peptide purification". The amounts of peptides app lied for the immune assays are also now specified in Methods and Fig. legend.

Fig. 5. It is unfortunately that the authors have missed the inclusion of OE mutant WT::ManSP28 mutant for parallel survival assays. This gain-of-function result would be essential to support the conclusion. In addition, unlike glucan and chitin/chitosa, commercial mannans may be water soluble. Its direct influence on insect survivals is unclear. A control treatment using pure mannan has not been included.

**Response**: We appreciate the reviewer's suggestion to rigorously validate the gain-of-function phenotype. In response, we have incorporated the survival bioassay for the SP28 overexpression strain (OE-*ManSP28*) into Fig. 5a; consistent with our model where SP28 functions as an immune elicitor, the overexpression strain displayed a trend of attenuated virulence compared to the wild type, although this difference did not reach statistical significance, likely due to a saturation effect where endogenous SP28 levels in the wild type already trigger a robust recognition response. Furthermore, to address the concern regarding the potential intrinsic toxicity of the inhibitor, we included appropriate controls in our bioassays and explicitly confirmed that the mannan concentration used (0.2%) has no negative impact on insect survival, demonstrating that the observed virulence enhancement results from specific checkpoint blockade rather than chemical toxicity.

The authors have not shown that ManSP28/MacSO28 is highly transcribed in fungal conidia. Not as described (lines 238-239), SP28 signal could not or be barely seen on the WT spore surface.

**Response**: We thank the reviewer for this important observation, which allows us to clarify our experimental strategy. All SP28 homologs analyzed in our study possess a predicted N-terminal signal peptide, supporting their potential for secretion. To directly demonstrate surface localization, we generated fungal strains overexpressing C-terminal FLAG-tagged SP28. Immunofluorescence staining with an anti-FLAG antibody confirmed the clear surface signal on these overexpression (OE) conidia, providing definitive proof-of-concept for SP28 secretion and surface localization (Supplementary Fig. 8c, d). The wild-type (WT) strain, which lacks the FLAG tag, served as the essential negative control for antibody specificity; therefore, the absence of signal in WT was the expected and correct result. Furthermore, transcript analysis confirmed that SP28 mRNA is detectable in wild-type *M. acridum* and *M. anisopliae* conidia (Supplementary Fig. 8b). Combined with the conserved

signal peptide, transcriptional evidence, functional data from conidial surface extracts (Fig. 4a), and the proof-of-concept localization in OE strains, we present a robust case for SP28's presence and critical role on the conidial surface.

The authors have shown that *Aspergillus flavus* does have a *ManSP28* homolog, however, which has not been expressed for IML1 cleavage assay. It may not localize on conidial surface.

**Response**: We thank the reviewer for raising this point. To directly address whether the *A. flavus* SP28 homolog (AfSP28) localizes to the conidial surface, we prepared a specific anti-AfSP28 polyclonal antibody. Immunofluorescence staining of wild-type *A. flavus* conidia confirmed the presence of the native AfSP28 protein on the spore surface (Supplementary Fig. 8a). This result demonstrates that AfSP28 is expressed and correctly trafficked in its native context. Its inability to cleave IML1 (Fig. 4a) therefore stems from an intrinsic divergence in enzymatic specificity, rather than a failure in surface localization.

There are also structuring and writing issues. The conclusive diagram Fig. S10 is put in supplementary, which appears before the last paragraph. The phylogenies of IML1 and ManSP28 proteins can be shown in supplementary just following their identifications. The authors show Fig. 5F and Fig. S6B, both for SP28 proteases. There is no results of molecular docking described in Methods.

**Response**: We sincerely thank the reviewer for their constructive suggestions regarding the organization and writing of our manuscript. In response, we have undertaken a comprehensive restructuring of the text and Figures to improve the narrative flow and clarity. To highlight the conceptual synthesis of our findings, we have elevated the conclusive mechanistic diagram from the supplementary material to the main text as Fig. 6, where it now serves as a

central visual summary in the Discussion section. To streamline the presentation and ensure a logical progression from molecular identification to functional analysis, we have consolidated the phylogenetic analyses of IML1 and SP28 into a new Supplementary Fig. 13. The corresponding descriptive text has been reorganized into a dedicated subsection within the Results, placed directly before the virulence bioassays.

We appreciate the opportunity to clarify the rationale for presenting two phylogenetic analyses of proteases. The analysis now in Supplementary Fig. 7c was a broad screen of all signal peptide-containing serine proteases from the fungi used in our initial experiments, serving to identify candidate proteases. In contrast, the analysis in Fig. 5f (and its expanded version in Supplementary Fig. 13b) specifically traces the evolution of Metarhizium SP28 orthologs across a wide taxonomic range of fungi. This focused comparison was essential to demonstrate the functional divergence correlated with pathogenicity, which is a cornerstone of our evolutionary argument. We have revised the relevant Fig. legends and text to make this distinction clearer.

Finally, regarding the molecular docking analysis, we confirm that the specific interaction energies are detailed in the Results (Line 130, 133), with the corresponding methodology fully described in the Materials and Methods section. We believe these adjustments have significantly improved the manuscript's logical structure and readability. Thank the reviewer for this valuable guidance.

Immulectin-1 for IML1 has not been indicated in Abstract etc. until the appearance in line 112.

**Response**: We thank the reviewer for identifying this oversight regarding nomenclature. To ensure immediate clarity for the reader, we have now explicitly defined the full name “immulectin-1” (IML1) upon its first citation in the Abstract (Line 19), updating the text to read: “...The host immulectin-1

(IML1)...”.

Line 190-191, two Fig. S5F? Fig. S5E, what are those abbreviations?

**Response**: We apologize for the typographical error in the figure citation and the lack of clarity regarding the abbreviations. We have corrected the erroneous citations in the revised manuscript (Line 214 and 216). Furthermore, we have thoroughly revised the legend for Supplementary Figure 6 to explicitly define all experimental groups and statistical details. The revised legend now clearly states: “…CK, non-infected check control; IF, infected with *M. acridum*; ds*GFP*, dsRNA targeting green fluorescent protein (negative control); ds*IML1*, dsRNA targeting *IML1*; ds*IML1/Ab*, ds*IML1* plus anti-IML1 antibody; rIML1, recombinant IML1 protein….”

The labels "IML1ac0/an0" and "IML1ac/an" could be briefly defined in the main text and Fig. legend for clarity.

**Response**: We thank the reviewer for this excellent suggestion to improve readability. Following this advice, we have added a clear definition for these labels in both the main text and the figure legend. In the main text (Lines 258-261), we now explicitly define the nomenclature when introducing the cleavage products. In the legend for Figure 4, we have included a concise note that clarifies: the suffixes ‘ac’ and ‘n’ refer to cleavage by proteases from *M. acridum* and *M. anisopliae*, respectively; the suffix ‘0’ designates the large, inactive fragment, while its absence designates the small, bioactive peptide. We believe these additions will prevent any ambiguity for readers.

Line 42: "through distincting from those against invasive pathogen." This phrase is grammatically incorrect. It should likely read something like: "through mechanisms distinct from those used against invasive pathogens" or "by distinguishing them from invasive pathogens."

**Response**: We thank the reviewer for identifying this grammatical error. We have revised the sentence according to the reviewer's excellent suggestion.

The “through distincting from those against invasive pathogen.” has been changed to “by discriminating them from invasive pathogens.”, as shown in Line 38.

Line 269-270: "SP28 delaying fungal infection through triggers host defences, thereby." This sentence is incomplete. It should be rephrased, e.g., "SP28 delays fungal infection by triggering host defenses."

**Response**: Thank the reviewer for pointing out this incomplete sentence. In the revised manuscript, we have restructured the final section of the Results, which involved removing the original sentence entirely. We have also taken the opportunity to thoroughly check the entire manuscript for potential grammatical issues and have made corrections accordingly.

Line 322: "This integrated systems is may represent..." change to "This integrated system may represent..."

**Response**: We apologize for this grammatical mistake. The sentence “This integrated systems is may represent” has been changed to “This integrated system may represent”, as shown in Line 417.

While generally clear, some Fig. captions (e.g., Fig. S10) could be slightly more descriptive for readers glancing at the supplement.

**Response**: We appreciate this valuable advice. We have reviewed all Fig. captions to ensure they are more descriptive and can be easily understood as stand-alone items. The model diagram, formerly in the supplement as Fig. S10, has been moved to the main text as Fig. 6, and its caption has been significantly expanded to describe each step of the proposed mechanism in detail.

## References

Miller, K.K. et al. (2024) SUB-immunogold-SEM reveals nanoscale distribution of submembranous epitopes. *Nat Commun* 15: 7864. https://doi.org/10.1038/s41467-

024-51849-x
Moussian, B. (2010) Recent advances in understanding mechanisms of insect cuticle differentiation. *Insect Biochem Molec*, 40: 363-375. https://doi.org/10.1016/j.ibmb.2010.03.003
Ortiz-Urquiza, A. and Keyhani, N. O. (2013) Action on the surface: entomopathogenic fungi versus the insect cuticle. *Insects*, 4: 357-374. https://doi.org/10.3390/insects4030357
Wang, C. S. and St Leger, R. J. (2007) The MAD1 adhesin of *Metarhizium anisopliae* links adhesion with blastospore production and virulence to insects, and the MAD2 adhesin enables attachment to plants. *Eukaryotic Cell*, 6: 808-816. https://doi.org/10.1128/ec.00409-06
Willmer, P. G. (1982) Microclimate and the environmental physiology of insects. *Adv Insect Physiol*, 16: 1-57. https://doi.org/10.1016/S0065-2806(08)60151-4

**Response to Reviewer #2**

Reviewer #2 (Remarks to the Author):

In this report, the authors identify an insect host lectin (IML1), expressed near the cuticle surface that recognizes mannans. The authors show that IML1 functions to activate immune responses only if cleaved by a fungal protease designated SP28. Although most fungi appear to contain SP28, only the SP28 from (some?) entomopathogenic fungi appears to be able to cleave IML1 into an active form which subsequently results in immune activation.

The report is very interesting, and the experiments well performed.

**Response**: We sincerely thank the reviewer for their positive assessment and constructive questions, which have guided several important improvements in this revision. In direct response to the specific points raised below, we have performed additional experiments and controls that further clarify the mechanism, specificity, and evolutionary context of the IML1-SP28 checkpoint. These include the use of the SunTag system to visualize IML1 trafficking, heterologous complementation to define protease specificity, phenotypic assays to resolve the role of SP28 in fungal fitness, and multiple critical controls such as heat-inactivated spores. We believe these additions have significantly strengthened the study, and we address each point in detail

below.

One conceptual issue should be addressed in the Discussion: why would the insect pathogen retain SP28 since its ablation increases virulence and conversely why would non-pathogens lack “active” SP28 since they aren’t pathogens to begin with?

**Response**: We thank the reviewer for raising this central evolutionary paradox, which prompted us to clarify the dual role of SP28. The apparent contradiction is resolved by recognizing that SP28 is subject to two opposing selective pressures. First, SP28 is essential for the fungus to overcome the host’s physical barrier. Our new phenotypic data (Supplementary Fig. 9) show that while knockout (KO) mutant grows normally on nutrient-rich medium, it is significantly impaired in colonizing the locust cuticle. Thus, for the pathogen, retaining *SP28* is non-negotiable, it is a key tool for invasion, and the benefit of cuticle degradation outweighs the cost of alerting the host immune system. Second, the hypervirulence of the KO-*SP28* mutant precisely reveals the potency of the host’s immune checkpoint. The fact that deleting *SP28* increases virulence demonstrates that the IML1-mediated immune response is a more formidable barrier to infection than the cuticle itself. The host has evolutionarily “weaponized” the fungus’s indispensable invasion tool (SP28 activity) as the most reliable signal of attack.

Regarding non-pathogens, their SP28 homologs have functionally diverged because they face no selective pressure to degrade insect cuticle. Without this constraint, their proteases have lost the specific activity required to cleave locust IML1. This functional drift explains why they lack the “active” key to the host’s surveillance “lock”. We have elaborated on this evolutionary framework in the revised manuscript, both in the Results (Lines 273-281) and in the Discussion, to provide a coherent explanation for the conservation and divergence of the SP28-IML1 system.

In addition:

1. What is the time point for the data in Fig. 1?

**Response**: Thank the reviewer for pointing out this omission. The time point for all immunological assays presented in Fig. 1 is 4 hours post-inoculation. We have now added this information to the legend for Fig. 1.

2. IML1 appears to have reasonable binding to glucans and lower binding to chitosan, but chitin (hydroxymethylchitin is more soluble) was not tested. What about response to glucans which are prevalent on fungal surfaces?

**Response**: We thank the reviewer for raising these crucial points regarding ligand specificity. In response, we have performed binding assays with chitin, revealing that IML1's affinity for chitin (Kd = 80.30 μg/mL) is an order of magnitude lower than for mannan (Kd = 2.24 μg/mL) or glucan (Kd = 7.40 μg/mL).

To functionally validate these interactions *in vivo*, we measured *IML1* transcriptional induction in hemocytes 4 hours post inoculation of *M. acridum*, mannan elicited the strongest upregulation (6.08-fold), followed by glucan (3.56-fold), whereas chitin induced no significant change (1.1-fold). While these data confirm that IML1 can indeed function as a glucan receptor, consistent with reports for the homologous CTL10 in *Bombyx mori* (Zhang, S. et al., 2021), the primacy of mannan in our model is dictated by the specific architecture of the fungal cell wall during the initial infection phase. Since our study focuses on the surveillance of intact spores prior to germination, the outermost mannan layer represents the accessible PAMP interface, effectively shielding the inner glucan and chitin layers (Gow, N.A.R., et al., 2017; Zhang, Y. et al., 2019). Thus, both biochemical affinity and spatial accessibility establish mannan as the dominant physiological trigger for this surface-level immune checkpoint.

3. What is the “stain” in Fig. 1D and why does A. alternata show no staining.

Binding of the recombinant IML1 appears to be lower to A. flavus (Fig. 2). Can the signal be measured to provide a more quantified analysis?

**Response**: We appreciate these questions, which prompted us to clarify our staining methods and provide quantitative validation. We have specified in the revised legend that surface mannans were visualized using FITC-labeled Concanavalin A (ConA) and have provided an optimized image in Fig. 2d that clearly displays mannan on *A. alternata*. Furthermore, addressing the request for quantification, we analyzed rIML1 binding fluorescence using ImageJ (new Fig. 2g). This analysis revealed no statistically significant difference in binding intensity between *M. acridum* and *A. flavus*, confirming that IML1 efficiently recognizes both pathogenic and non-pathogenic spores. Accordingly, we have updated the result (Lines 141-143).

4. The background control in Fig 3B is very high (compare to the background/signal in Fig. S4B). More importantly, shouldn't the samples be examined as a cross-section as given in panel A? We should see a similar pattern between the mRNA and antibody? How is IML1 anchored to the cuticle, it appears to be a soluble protein?

**Response**: We thank the reviewer for these insightful observations, which prompted us to significantly improve our imaging strategy and mechanistic explanation. Regarding the visualization of IML1 distribution: We agree that the previous immunogold TEM images, while showing local detail, were limited in their ability to display the overall tissue context. To address this, we have replaced the original panels with high-resolution immunofluorescence (IF) microscopy of cross-sectioned cuticles using a specific anti-IML1 antibody (revised Fig. 3b and Supplementary Fig. 4b). These new cross-sectional images clearly delineate the distribution of IML1 from the epidermal cells through the procuticle to the epicuticle. Importantly, this protein localization pattern aligns perfectly with our mRNA *in situ* hybridization (FISH) results (Fig. 3a) and qPCR data (Supplementary Fig. 4a), providing a consistent and

robust spatial profile.

Regarding the anchoring mechanism of the soluble IML1: This is a critical biological question. While IML1 lacks a transmembrane domain, our structural modeling reveals that IML1 possesses surface-exposed hydrophobic patches that are comparable in extent to its hydrophilic regions (Supplementary Fig. 2d). We propose that these hydrophobic patches facilitate non-covalent association with the lipid-rich epicuticle, providing a plausible mechanism for its surface localization. Furthermore, our new SunTag *in vivo* tracking experiment (Supplementary Fig. 5) provides direct evidence that IML1 is actively transported from the hemolymph to the body surface upon fungal challenge. Synthesizing these findings, we propose a dynamic replenishment model: rather than being permanently anchored, IML1 at the cuticle surface is likely maintained through continuous replenishment from the systemic pool in the hemolymph (where it is basally expressed), with its surface retention stabilized via the aforementioned hydrophobic interactions. This model ensures a rapid and sustained accumulation of IML1 around invading spores.

Consistent with this dynamic view, the insect epicuticle itself is not static but is renewed during development. Most critically, as argued in our response to Reviewer #1, when a spore attaches, it creates a localized, water-rich microenvironment at the spore-cuticle interface. Within this specific niche, IML1 would be soluble and functionally active, perfectly positioned to interact with fungal proteases like SP28. Thus, its proposed mechanism of action is fully compatible with its dynamic presence at this active interface.

5. *B. bassiana* should be included in Fig. 4A. Figure legend should include time points used for each panel.

**Response**: We thank the reviewer for these excellent suggestions to improve the completeness of our study. We have now performed the *in vitro* cleavage assay using surface protein extracts from *Beauveria bassiana* and have

included this new data in the revised Fig. 4a. The result is clear: the B. bassiana extract efficiently cleaves recombinant IML1, producing a cleavage pattern highly similar to that of *Metarhizium* species. Furthermore, as suggested, we have carefully reviewed and revised all Fig. legends throughout the manuscript to ensure that critical experimental details, such as incubation time points and sample information, are clearly and consistently stated. We appreciate the reviewer pointing out these omissions, and we are confident the added details have improved the clarity and reproducibility of our work.

6. According to Fig. 5F, the “active” Clade G SP28 includes plant pathogenic fungi. Does the SP28 from these result in IML1 activation? If so, that would make the issue of specificity more complex. Related: Panels E and F are not that informative as few organisms are labeled. It may be better to indicate which Class/Order either IML1 and/or SP8 are present. At which point do the authors consider specificity to have been affected?

**Response**: We thank the reviewer for raising these important points regarding phylogenetic interpretation and functional specificity.

In response to the comment on figure clarity, we have revised the phylogenetic trees to display all species names and annotated them with higher taxonomic ranks (Class for IML1, Phylum/Class for SP28 in Supplementary Fig. 13a, b). This provides clearer evolutionary context.

Regarding the presence of plant-pathogenic fungi within the “active” Clade G, this pattern is consistent with the known evolutionary history of the order Hypocreales, which is characterized by repeated host shifts between plants and insects (Spatafora et al., 2007). The inclusion of fungi such as Claviceps purpurea in a clade dominated by entomopathogens therefore reflects this dynamic history of host switching, rather than contradicting the specificity of the mechanism.

Most importantly, our heterologous complementation experiment provides direct functional evidence for specificity. We showed that the SP28 ortholog from the phylogenetically distant, non-entomopathogenic fungus Aspergillus flavus failed to restore immune activation or virulence in a Metarhizium Δ SP28 mutant. This result clearly demonstrates that the ability to cleave locust IML1 is not a general property of all fungal SP28 proteases, but a highly specialized trait that has evolved within entomopathogenic lineages.

While the precise functional status of SP28 in the closely related plant-pathogenic species remains a fascinating subject for future study, our current data robustly support a model in which SP28-mediated activation of the IML1 checkpoint is a specific adaptation tightly linked to insect pathogenesis.

7. Data for the SP28 complemented mutant appears to be missing to confirm the genetics.

**Response**: We appreciate the reviewer's emphasis on genetic rigor. In response, we have generated the *SP28* complemented strain (CP) and performed a comprehensive phenotypic characterization. The results confirm that reintroducing the gene fully restores wild-type phenotypes, including IML1 cleavage efficiency, host immune activation, and overall virulence, thereby verifying that the observed effects are specific to the loss of SP28. These complementation data have been integrated into the revised manuscript in Fig. 4g-i, Fig. 5a, and Supplementary Fig. 9 and 10.

8. Considering Fig. S6B, can the *P. lilacinum* and/or *B. bassiana* but not *A. flavus* and/or *A. alternata* SP28 genes complement the SP28 mutant in *M. acridum*?

**Response**: We appreciate this insightful suggestion. To test functional conservation, we generated *M. anisopliae* strains in which the native SP28 was replaced *in situ* with orthologs from the entomopathogen *Beauveria bassiana* (BbSP28) or the non-entomopathogen *Aspergillus flavus* (AfSP28).

The entomopathogen-derived BbSP28 fully restored the ability to trigger host immunity (Fig. 4g-i) and wild-type virulence (Fig. 5a). In contrast, AfSP28 failed to complement these functions, resulting in a non-immunogenic and hypervirulent phenotype identical to the KO-*SP28* mutant. This result provides definitive genetic evidence that the capacity to activate the IML1 checkpoint is a specialized trait of entomopathogenic SP28 variants. The inclusion of AfSP28 serves as a critical negative control, strengthening the *in vitro* cleavage results and providing functional support for the phylogenetic divergence observed in our analysis (Supplementary Fig. 13b).

9. Are there any other phenotypic consequences of loss of SP28 for the fungus? This would relate to why it may be maintained.

**Response**: We thank the reviewer for raising these important questions regarding the ligand specificity of IML1. Our comprehensive biochemical assays determined that recombinant IML1 (rIML1) possesses a clear hierarchy of binding affinity: it binds most strongly to mannan (Kd = 2.24 μg/mL), followed by glucan (Kd = 7.40 μg/mL), and with significantly weaker affinity to chitin (Kd = 80.30 μg/mL) (Fig. 2a-c). Functionally, topical application of purified β-glucan did induce IML1 transcription in hemocytes *in vivo*, confirming its capacity as a ligand. However, the immune activation observed specifically during the initial spore attachment phase, the focus of our study, is predominantly mediated by mannan recognition.

This specificity is best explained by the spatial and temporal context of host-pathogen interaction. The fungal spore wall is stratified, with mannan constituting the outermost layer, making it the primary and immediately accessible target during the pre-germination stage. Our functional data using mannan-blocking antibodies directly support this, as they abolished the early immune response (Supplementary Fig. 6a-d). In contrast, inner wall components like β-glucan and chitin become exposed predominantly after spore germination and penetration, which would engage different or additional

recognition pathways. Thus, while IML1 can bind glucan, our work establishes that the surveillance of intact spores at the host surface is gated primarily through its interaction with surface mannan.

10. IML1 appears to be most highly expressed/induced in hemocytes (Fig. S3C). How does this fit with the model the authors suggest that it's activity is mainly near/underneath the cuticle? Examining slightly longer time points, i.e. 12-18 h may also be informative as this would represent spore germination?

**Response**: We appreciate the reviewer's insightful observation regarding the spatial dynamics of IML1 expression. Our model integrates the high hemocytic expression of IML1 with its critical function at the cuticle through an active, inducible transport mechanism. Using the *in vivo* SunTag live-imaging system, we directly visualized IML1 secretion from the hemolymph and its progressive accumulation around fungal conidia on the cuticle surface (Supplementary Fig. 5). This provides definitive evidence that the functional pool of IML1 at the host-pathogen interface is actively recruited from the systemic circulation, a process analogous to the relocalization of other immune factors (e.g., prophenoloxidase) in insects (Ashida, M. and Brey, P.T., 1995).

To further address the reviewer's suggestion regarding longer time points, we extended our transcriptional analysis. We found that IML1 expression exhibits a biphasic pattern: after the early peak at 4 h, transcript levels decline by 6 h, followed by a second, gradual increase up to 16 h post-inoculation (Supplementary Fig. 3c). Notably, this expression dynamics aligns with the established timeline of fungal development on the locust cuticle from our previous work: conidial germination initiates around 6-8 h (Zheng, X. et al., 2020), followed by penetration and invasive growth. Thus, the first transcriptional wave corresponds to the pre-germination attachment phase, during which IML1 is mobilized to the surface to enact the protease-gated surveillance described here. The later induction likely reflects IML1's role in

the subsequent systemic immune response against established infection.

By focusing our functional analysis on the 0-4 h window, we deliberately isolated the initial, pre-invasion recognition event mediated by the IML1-mannan-SP28 checkpoint. Later time points (≥12 h) involve fungal growth, cuticle penetration, and exposure of inner cell-wall components such as β-glucans, which trigger broader, overlapping immune cascades that would obscure the specificity of the early surveillance mechanism we aimed to characterize.

11. The timing of IML1 expression (Fig. 3) seems confusing. IML1 expression is low to begin with but is induced to high levels at 4 hr

**Response**: We sincerely thank the reviewer for highlighting this important point regarding the mRNA expression pattern. Upon careful re-inspection, we agree that the initial fluorescence *in situ* hybridization (FISH) images in Fig. 3a may not have optimally represented the basal IML1 mRNA levels, potentially due to technical variability in tissue sectioning or hybridization efficiency for the time-zero sample. To resolve this unequivocally, we have repeated the entire FISH experiment with freshly prepared tissue sections. The new data, now presented in the revised Figure 3a, clearly show a low but detectable basal level of IML1 transcripts in epidermal cells at 0 h, followed by a time-dependent increase in signal intensity, which is entirely consistent with the quantitative trends from our qRT-PCR analysis (Supplementary Fig. 3c). We regret for the confusion caused by the initial images and are confident that the revised data accurately depict the transcriptional induction of IML1 upon fungal challenge.

12. Do heat killed *M. acridum* spores +/- SP8 bind to IML1 and/or activate immune responses? This would seem to be an important control.

**Response**: This is an excellent suggestion for a control experiment. We performed the experiment and found that heat-killed spores of both *M.*

*acridum* and *M. anisopliae* (wild-type and KO-*SP28* mutants) retained, and often exhibited enhanced, binding to IML1 compared to live spores (Supplementary Fig. 11a, b), likely due to heat-induced exposure of cell wall mannans. However, and crucially, these heat-killed spores completely failed to activate any of the measured host immune responses, including cuticular PO activity, defensin expression, and hemocyte activation (Fig. 4g-i). This definitive uncoupling of IML1 binding from immune activation underscores the protease-gated nature of the checkpoint: immune signaling is licensed not by mere receptor occupancy, but specifically by the proteolytic cleavage of IML1 by active fungal SP28.

13. *B. bassiana* should be included in Fig. 4A.

**Response**: We thank the reviewer for this constructive suggestion. As requested, we have now included *Beauveria bassiana* in the conidial surface protein extraction and cleavage assay. The updated Fig. 4a demonstrates that the surface protein extract from *B. bassiana* conidia, like those from *Metarhizium* species, is capable of cleaving recombinant IML1. This result further strengthens our conclusion that the proteolytic activity required to trigger the immune checkpoint is a conserved feature among entomopathogenic fungi, thereby reinforcing the broader relevance of the proposed IML1-SP28 surveillance mechanism. We have updated the Fig. and the corresponding text in the Results section (Lines 235-236) accordingly.

**References**


Spatafora, J.W. et al. (2007). Phylogenetic evidence for an animal pathogen origin of ergot and the grass endophytes. *Mol. Ecol.* 16: 1701-1711. https://doi.org/10.1111/j.1365-294X.2007.03225.x

Ashida, M. and Brey, P. T. (1995) Role of the integument in insect defense: pro-phenol oxidase cascade in the cuticular matrix. *Proc. Natl. Acad. Sci. USA* 92: 4069-4073. https://doi.org/10.1073/pnas.92.23.10698

Zheng, X.L. et al. (2020). Locust can detect beta-1, 3-glucan of the fungal pathogen before penetration and defend infection via the Toll signaling pathway. *Dev. Comp. Immunol.* 106: 103636. https://doi.org/10.1016/j.dci.2020.103636

Zhang, S. et al. (2021) CTL10 has multiple functions in the innate immune responses of

the silkworm, *Bombyx mori*. *Dev. Comp. Immunol.* 179: 104969. https://doi.org/10.1016/j.dci.2021.104309

Gow, N.A.R. et al. (2017) The fungal cell wall: structure, biosynthesis, and function. *Microbiol Spectr.* 5: FUNK-0035-2016. https://doi.org/10.1128/microbiolspec.FUNK-0035-2016

Zhang, Y. et al. (2019). O-mannosyltransferase *MaPmt2* contributes to stress tolerance, cell wall integrity and virulence in *Metarhizium acridum*. *J Invertebr Pathol.* 184: 107649. https://doi.org/10.1016/j.jip.2021.107649

**Reviewer #1 (Remarks to the Author)**:
Additional experiments and repetition of some experiments have been done in this revision. Taken together with re-writing and re-structuring, substantial improvements have been made, and the answers of concerns are generally acceptable. Remaining corrections and clarifications are still required.

**Response**: We sincerely thank the reviewer for the positive evaluation of our revised manuscript and for recognizing the substantial improvements made through the additional experiments and restructuring. We value the reviewer's continued attention to detail, particularly regarding sequence accuracy and data presentation. In this final round of revision, we have meticulously addressed all remaining corrections and clarifications, including verifying accession numbers, refining the discussion of the virulence trade-off, and correcting figure details, as described in a point-by-point manner below. Furthermore, to ensure full transparency and reproducibility, we have compiled all primer/probe sequences and gene accession numbers used in this study into Supplementary Data 1 and 2, respectively, and have cited them in the Materials and Methods.

There are issues for the reported sequences/accessions. Table S1, LOCMI10396 is not a lectin. Line 263, EFY85443 for MacSP28 but Table S3 shows that EFY87133 is for ManSP28 (sic). EVM0008168 is not accessible in public databases.

**Response**: We sincerely apologize for these oversights and thank the reviewer for the meticulous validation of our accession numbers. We have rigorously re-verified all sequences and updated the manuscript and supplementary tables accordingly: (1) The accession LOCMI10396 was indeed a typographical error; the correct accession for the locust C-type lectin (IML1) is LOCMI10363. This has been corrected in Table S1. (2) The EVM identifiers previously used corresponded to a specific genome assembly version. To ensure public accessibility, we have mapped these to the standard GenBank accession numbers. In Table S3, EVM0008168 (ManSP28) has been updated to KAK8918209.1, and EVM0010142 has been updated to KAK8922447.1. (3) To ensure consistency and avoid potential confusion, we have removed the specific accession numbers from the main text (Line 264). The correct and verified accession numbers for MacSP28 (*M. acridum*) and ManSP28 (*M. anisopliae*) are now consolidated and listed in Table S3.

The remaining controversy results are the data regarding the ManSP28/MacP28's contribution to invasion and IML1 cleavage. Fig. S9 data indicated that KO strain was impaired in penetrating locust wings (implication in degrading cuticular compounds. In contrast, however, locust survival data showed in Fig. 5 indicated that the LT50 of KO strain was reduced whereas the LT50 of OE strain was increased, i.e., the virulence of KO strain was increased but OE strain had reduced virulence. Even though the data fit with

SP28's role in cleaving IML1 to activate locust immune responses, the results did not align with its proposed "additional" involvement in cuticle penetration and host invasion.

**Response**: We thank the reviewer for highlighting this intriguing point. We respectfully submit that this result is not a contradiction, but rather reveals a critical biological trade-off where host immune surveillance acts as a stricter bottleneck to successful infection than the physical cuticle barrier. In the sterilized wing assay, which represents a purely physical and immunologically inert barrier, the ΔManSP28 mutant indeed grows and penetrates significantly slower than the WT (Fig. S9). This observation confirms SP28's physiological role in degrading the structural components of the cuticle. However, in the live host, which presents a dynamic defense system, the mutant effectively adopts a stealth strategy. By sacrificing rapid penetration, the mutant successfully bypasses the potent IML1-mediated immune checkpoint, leading to a drastically reduced host immune response (Fig. 4g-i).

To directly address how a slower-penetrating fungus ultimately causes faster host mortality, we quantified the actual fungal burden inside the host. Crucially, our data show that despite its initial mechanical disadvantage at the surface, the ΔSP28 mutant achieves a significantly higher fungal DNA load in the hemolymph compared to the WT or OE strains (Supplementary Fig. 10). This finding bridges the conceptual gap perfectly: it demonstrates that for the WT strain, the kinetic benefit of rapid penetration is severely negated by the cost of triggering immune clearance. Conversely, once the stealthy mutant eventually breaches the physical barrier, it proliferates unchecked in the hemolymph because it evaded initial detection. This vastly higher internal fungal biomass directly accounts for the accelerated host mortality (Fig. 5). This mechanism aligns with classic findings in fungal pathogenesis (Wang & St. Leger, *PNAS* 2006; Gottar, M., et al., *Cell* 2006), where immune evasion is often the dominant determinant of hypervirulence. We have expanded the Discussion to fully clarify this hierarchy of defense and the resulting evolutionary trap.

The proposed "lock-and-key" model is acceptable but it is confusing regarding the discussion/suggestion (lines 430-444), which argued that "the primary role of SP28 is not to engage the host immune system, but to perform a fundamental physiological function for the fungus." or "host immunity activation is not SP28's primary function, but rather an unavoidable consequence of its essential activity during infection. "

**Response**: We agree that our previous phrasing was confusing, and we have completely revised this section in the Discussion to remove the ambiguous terminology and clarify the evolutionary context of our lock-and-key model. Our original intention was to highlight the evolutionary dilemma the pathogen faces. We have now clarified this by framing it as an evolutionary trade-off.

Mechanistically, because SP28 is indispensable for breaching the cuticle, its retention is non-negotiable for the fungus. The host has evolutionarily co-opted this essential pathogenic activity as a reliable signature of attack. Therefore, in our revised text, we explicitly state that SP28 evolved as an invasion tool (the key), but inadvertently became the precise target of the host's IML1 receptor (the lock). This dynamic perfectly explains why the pathogen cannot simply discard the key to evade immunity, doing so would strip it of its ability to invade, forcing it to retain a factor that betrays its presence. We believe this updated narrative resolves the previous confusion and clearly articulates how reciprocal selection drives both the retention of SP28 and the strict host specificity observed in our study.

Line 171, "IML1 levels significantly increased in the integument" (Fig. S3d). Actually, band intensity has not been quantified, and it looks that the increase of band intensity is marginal.

**Response**: We appreciate the reviewer's careful observation regarding the Western blot data. We agree that the increase in the integument is less dramatic compared to that in the hemolymph. Accordingly, we have revised the text (Line 171-173) to more accurately describe the results without implying statistical significance. We now state that IML1 levels showed a "moderate increase" in the integument, distinguishing it from the substantial upregulation observed in hemocytes and hemolymph.

Lines 178-179, Fig. S4b is not a TEM analysis.

**Response**: We apologize for this oversight in the figure citation. We have corrected the text to accurately describe Figure S4b as "IF analysis" rather than "Transmission electron microscopy (TEM)".

Fig. S6: x-axis labelings are missed in different panels.

**Response**: We thank the reviewer for pointing this out. In the previous version, we omitted the labels for panels (b-d) to minimize visual redundancy, as they shared the same treatment groups as panel (a). However, we agree that explicit labeling is better for independent readability. We have revised Fig. S6 to include specific x-axis labels for every panel to avoid any potential confusion.

References

Gottar, M. et al. (2006) Dual detection of fungal infections in Drosophila via recognition of glucans and sensing of virulence factors. *Cell* 127, 1425-1437.

Wang, C. S. & St. Leger, R. J. (2006) A collagenous protective coat enables Metarhizium anisopliae to evade insect immune responses. *P Natl Acad Sci USA* 103, 6647-6652.

**Reviewer #2 (Remarks to the Author)**:

The authors have done an excellent job at addressing the previous concerns and have supplemented the work with significant new data.

**Response**: We sincerely thank the reviewer for the highly positive evaluation of our revised manuscript. We are pleased that the additional experiments and structural changes have satisfactorily addressed your concerns and strengthened the conclusions of our study.

The only minor item concerns the title which is a bit difficult to understand. Would suggest altering to:

1. Insect hosts recognize a fungal pathogen derived protease to activate immune defenses
Or
2. Immune surveillance on the insect body surface recognizes a pathogen-derived fungal protease to activate defenses

**Response**: We appreciate the thoughtful suggestion to improve the accessibility and clarity of our title. We fully agree that the second option offers a more precise description of our findings by highlighting the specific location of the interaction. Accordingly, we have adopted the second suggestion and changed the title to: "**Immune surveillance on the insect body surface recognizes a pathogen-derived fungal protease to activate defenses**."
